# Aggregation operators based on Einstein averaging under q-spherical fuzzy rough sets and their applications in navigation systems for automatic cars

**DOI:** 10.1016/j.heliyon.2024.e34698

**Published:** 2024-07-20

**Authors:** Ahmad Bin Azim, Asad Ali, Abdul Samad Khan, Fuad A. Awwad, Sumbal Ali, Emad A.A. Ismail

**Affiliations:** a Department of Mathematics and Statistics, Hazara University Mansehra, 21300, Khyber Pakhtunkhwa, Pakistan; b Research Center for Computational Science, School of Mathematics and Statistics, Northwestern Polytechnical University, Xi'an, 710129, China; c Department of Quantitative Analysis, College of Business Administration, King Saud University, P.O. Box 71115, Riyadh, 11587, Saudi Arabia

**Keywords:** q-Spherical fuzzy rough sets, Aggregation operators, Multiple-criteria decision-making

## Abstract

This study introduces innovative operational laws, Einstein operations, and novel aggregation algorithms tailored for handling q-spherical fuzzy rough data. The research article presents three newly designed arithmetic averaging operators: q-spherical fuzzy rough Einstein weighted averaging, q-spherical fuzzy rough Einstein ordered weighted averaging, and q-spherical fuzzy rough Einstein hybrid weighted averaging. These operators are meticulously crafted to enhance precision and accuracy in arithmetic averaging. By thoroughly examining their characteristics and interrelations with existing aggregate operators, the article uncovers their distinct advantages and innovative contributions to the field. Furthermore, the study illustrates the actual implementation of these newly constructed operators in a variety of attribute decision-making scenarios employing q-SFR data, yielding useful insights. Our suite of decision-making tools, including these operators, is specifically designed to address complex and uncertain data. To validate our approach, this study offers a numerical example showcasing the real-world applicability of the proposed operators. The results not only corroborate the efficacy of the proposed method but also underscore its potential significance in practical decision-making processes dealing with intricate and ambiguous data. Additionally, comparative and sensitivity analyses are presented to assess the effectiveness and robustness of our proposed work relative to other approaches. The acquired knowledge enriches the current understanding and opens new avenues for future research.

## 1 Introduction

Zadeh [1] introduced the concept of fuzzy sets, which inspired subsequent extensions such as interval-valued fuzzy sets (IVFSs) [2], Atanassov's intuitionistic fuzzy sets (IFSs) [3], and interval-valued intuitionistic fuzzy sets (IVIFSs) [4] as alternative structures within the fuzzy set framework. However, Zadeh's study [5] and other studies [6,7] have demonstrated that Atanassov's intuitionistic fuzzy sets (IFS) and IVFSs are mathematically equal. IFS is closely linked to the representation of intuitionistic fuzzy information, necessitating a thorough understanding of both intuitive fuzzy sets and aggregation methods [8]. The concept of IFSs has evolved, allowing non-membership and membership functions to take interval values, leading to the development of interval-valued intuitionistic fuzzy sets (IVIFSs) [9]. IVIFS offers a more complicated representation of uncertainty and imprecision in fuzzy set theory. The invention of intuitionistic fuzzy numbers (IFNs) was a significant step towards determining decision outcomes. To elaborate, Xu [10] presented other aggregation operators, including IFWA, IFOWA, and IFHA, and detailed their unique properties. Notably, Xu and Yager [11] introduced three new geometric aggregation operators for IFNs: IFWG, IFOGA, and IFHG. The application of these mathematical principles to a wide range of topics and situations is intriguing. Zeng and Su [12] examined the development of an intuitionistic fuzzy ordered weighted distance (IFOWD) operator and its use in group decision-making for system selection. Their finding throws light on this vital location, paving the way for future discoveries. The IFOWD operator shows fascinating applications throughout a wide spectrum. When decision-makers express preference values like $\zeta_{A}=0.6\;\text{and}$ $\xi_{A}=0.7$, the total of both values exceeds 1. This violates the criterion of an intuitionistic fuzzy set. Yager's Pythagorean fuzzy sets [13] solve these problems by ensuring that $\left(\;{\left(\zeta_{A}\right)}^{2}+{\left(\xi_{A}\right)}^{2}\right)\leq 1$. Pythagorean fuzzy sets are recognized to manage uncertainty better than intuitionistic fuzzy sets (IFSs), making Pythagorean fuzzy set theory a more popular and exciting research topic. Yager and Abbasov [14] developed many aggregation approaches to address Multiple Criteria Decision Making (MCDM) issues in the Pythagorean fuzzy framework. Neutronosophic cubic sets [15] are derived from the neutrosophic set [16], which represents an astounding step forward beyond conventional fuzzy sets. According to the existing literature, major research efforts have been focused on the study of neutrosophic sets (NSs), neutrosophic cubic sets (NCSs), and the accompanying aggregation operators. Alia et al. [17] investigate the notion of NCSs and their use in pattern recognition. Furthermore, Je [18] developed operations and aggregation methods specifically for NCSs. Ajay et al. [19] used geometric Bonferroni mean operators for multicriteria decision-making (MCDM) using neutrosophic cubic sets (NCSs). Coung et al. [20] introduced the concept of picture fuzzy sets, with the restriction that the sum of all memberships lies inside the interval [0,1]. In more recent work, Atta et al. [21] used the notion of neutrosophic sets (NSs) in an upgraded picture steganography system that is based on modification direction. Gundogdu et al. [22] developed the notion of spherical fuzzy sets (SFS) and its associated theory, describing it as a unique extension of fuzzy set theory. This paradigm is differentiated by its triple membership structure, which contains membership, non-membership, and hesitation functions. They look at the positive, neutral, and negative membership functions with total squares equal to or less than one. In dealing with uncertainty, imprecision, and vagueness, the SFS model surpasses Pythagorean fuzzy sets. A thorough examination of the most recent literature demonstrates a growing preference for investigations into SFS. Ashraf et al. [23] developed a set of aggregation strategies, particularly for a spherical fuzzy framework. Ashraf et al. [24] took a unique approach by providing a grey technique (GRA) based on the groundbreaking notion of spherical linguistic fuzzy Choquet integrals. Furthermore, Jin et al. [25] developed and utilized logarithmic operators designed for spherical fuzzy sets (SFSs) in decision support systems. Rafiq et al. [26] introduced a cosine similarity measure tailored particularly to the SFS model, intending to improve decision-making in scenarios including ambiguous and imprecise data. Furthermore, Ashraf et al. [27] proposed a group decision-making technique customized for the spherical fuzzy environment and used it to solve challenges in multi-criteria group decision-making (MCGM). Gundogdu et al. [28] updated the well-known VIKOR technique to include the spherical fuzzy set (SFS) model and used it in a Multi-Criteria Decision Making (MCDM) context within a spherical fuzzy setting. Acharjya and Rathi [29] proposed an integrated decision-making approach that combines fuzzy rough sets and genetic algorithm models. They evaluated its effectiveness in a relevant MCDM scenario about smart agriculture. Sharaff et al. [30,31] investigated a fuzzy-based technique for text summarization extraction and proposed a document categorization strategy based on a fuzzy clustering algorithm. Gou et al. [32] devised exponential operating rules for interval fuzzy sets (IFSs) and introduced novel aggregation operators within the IFS framework. Recent developments in decision-making methodologies have utilized diverse fuzzy set theories and aggregation operators to address intricate problems in various fields. For instance, Seikh and Mandal [33] developed interval-valued Fermatean fuzzy Dombi aggregation operators and applied the SWARA-based PROMETHEE II method for effective biomedical waste management. In another study, Seikh and Chatterjee [34] utilized the SWARA-ARAS method in a confidence level-based interval-valued Fermatean fuzzy environment to determine the best renewable energy sources in India. Mahmood et al. [35] proposed a decision-making and medical diagnosis method using spherical fuzzy sets. In contrast, Wang [36] employed T-spherical fuzzy rough interactive power Heronian mean aggregation operators for multiple attribute group decision-making. Furthermore, Wang et al. [37] created a new CODAS approach using the Heronian Minkowski distance operator for T-spherical fuzzy group decision-making, while Ali et al. [38] introduced Heronian mean operators for multi-attribute decision-making using T-spherical fuzzy information. Using bipolar complex fuzzy soft information, Jaleel [39] extended the WASPAS approach to agricultural robotics systems based on Dombi aggregation operators. For MADM problems, Ali et al. [40] investigated averaging aggregation operators in the context of q-rung orthopair image fuzzy soft sets. Additionally, Azim et al. [41] applied sine trigonometric q-SFR aggregation operators for group decision-making in digital transformation [42], evaluated indoor positioning systems using a q-SFR TOPSIS analysis [43], and prioritized Industry 4.0 projects using this method.

### 1.1 Logics to define Einstein aggregation operators and their advantages

Einstein operations, a subset of fuzzy logic operators, are used to create aggregation operators. These operators are used to aggregate multiple fuzzy sets or fuzzy values into a single fuzzy set or value. The logic behind Einstein's aggregation is derived from Einstein's addition and multiplication operations, which provide a way to combine values while considering their inherent fuzziness. Below are the advantages of Einstein aggregation operators.

1. Einstein aggregation operators handle the fuzziness in the input values more naturally compared to traditional aggregation operators. This results in a more accurate representation of the aggregated value.
2. These operators maintain consistency within the framework of fuzzy logic, ensuring that the aggregated results align with the principles of fuzzy set theory.
3. Einstein aggregation operators are effective in dealing with extreme values and outliers, providing a more balanced aggregation that is not unduly influenced by such values.
4. These operators can be applied in various fields where fuzzy logic is used, such as decision-making, pattern recognition, and control systems, enhancing the flexibility and applicability of fuzzy models.
5. The results obtained through Einstein aggregation are often easier to interpret in the context of fuzzy logic, aiding in better decision-making and analysis.

Einstein aggregation operators are introduced to leverage the unique properties of Einstein operations in handling fuzzy data. The Einstein operations, derived from the Einstein sum and product, are known for their ability to maintain boundedness and smoothness, which are crucial for dealing with complex and uncertain data. These properties help in reducing the overestimation and underestimation issues that can arise with traditional arithmetic operations. Additionally, Einstein operations provide a more flexible and generalized framework for combining fuzzy data, allowing for better accommodation of the inherent uncertainties and interdependencies among attributes in decision-making scenarios. The Einstein aggregation operators, including the q-spherical fuzzy rough Einstein weighted averaging, ordered weighted averaging, and hybrid weighted averaging operators, are designed to enhance the precision and accuracy of the arithmetic averaging process. This is achieved by leveraging the inherent properties of Einstein operations, which handle fuzzy and rough data more effectively compared to traditional methods. Unlike the Dombi aggregation operators used in Mandal and Seikh's interval-valued spherical fuzzy MABAC method [44], Einstein operators offer superior handling of uncertainties by providing a more robust and flexible aggregation framework. This is particularly beneficial in scenarios where data uncertainty and fuzziness are prominent. The q-rung orthopair fuzzy Archimedean aggregation operators introduced by Seikh and Mandal [45] focus on a specific type of fuzzy environment. In contrast, Einstein aggregation operators are more adaptable to various fuzzy environments, including q-spherical fuzzy rough data, offering broader applicability in different decision-making contexts. The newly designed Einstein aggregation operators provide clearer insights into the interrelations and characteristics of aggregated data. This contrasts with the integrated weighted distance-based approximation method for interval-valued spherical fuzzy MAGDM presented by Mandal and Seikh [46], which, while effective, may not offer the same level of detail in understanding data interdependencies. The practical application of Einstein aggregation operators in multiple attribute decision-making scenarios demonstrates their effectiveness in real-world settings. The comparative and sensitivity analyses provided in the study further validate the robustness and efficacy of these operators, showing potential advantages over the methods discussed in Refs. [[44], [45], [46]]. The Einstein aggregation operators introduce significant improvements in precision, accuracy, and handling of uncertainty, making them advantageous over the Dombi aggregation operators, q-rung orthopair fuzzy Archimedean aggregation operators, and the integrated weighted distance-based approximation method discussed in the cited references. These enhancements make Einstein aggregation operators a valuable addition to the toolkit for addressing complex and uncertain data in decision-making processes. Kahraman et al. [47] proposed the novel notion of a q-q-SFS, which has proven extremely useful in assisting informed decision-making procedures. This concept is often regarded as an extension of the standard “q-spherical fuzzy set,” with each component falling within the q-SFS framework. The components $\zeta_{A}$, $\eta_{A}$, and $\xi_{A}$ are classified as positive, neutral, or negative, respectively. It is essential to adhere to the constraint ${\left(\zeta_{A}\right)}^{q}+{\left(\eta_{A}\right)}^{q}+{\left(\;\xi_{A}\right)}^{q}\leq 1$. This ensures that the total q-powers of $\zeta_{A}$, $\eta_{A}$, and $\xi_{A}$ do not exceed one, allowing the q-SFS to accommodate a wide range of responses, including positive and negative assessments, uncertainty, and even abstention from answering. By incorporating the parameter q, the q-SFS provides decision-makers with greater flexibility to express their preferences for membership, non-membership, and the degree of ambiguity in their judgments. This enhances decision-making by offering a more nuanced approach to handling complex and ambiguous information, thereby increasing the practical value of q-SFS in various application areas.

Rough set theory, introduced by Pawlak [48,49] in the early 1980s, is a mathematical approach designed to handle vagueness and uncertainty in data analysis, especially when information is imprecise or incomplete. At its core, rough set theory revolves around the concept of an information system, which is essentially a table comprising objects and their attributes. Objects are considered indiscernible if they cannot be distinguished based on the available attributes, leading to the formation of equivalence classes. The theory defines two key approximations: the lower approximation, which includes all objects that belong to a target set, and the upper approximation, which includes all objects that possibly belong to the target set. The boundary region, representing the difference between these approximations, signifies the uncertainty in classification. Rough set theory is also concerned with identifying reducts, which are minimal subsets of attributes that retain the classification capability of the entire attribute set, and the core, which is the intersection of all reducts, representing the most essential attributes. These concepts are particularly useful in various applications, such as feature selection, where the theory helps identify the most relevant attributes in a dataset, reducing dimensionality and improving machine learning efficiency. In data mining and knowledge discovery, rough set theory aids in uncovering patterns and relationships within data, providing valuable insights. It is also beneficial in decision support systems by offering a framework to manage imprecise information for making informed decisions, and in classification and clustering tasks, it helps group objects based on their attributes even amidst uncertainty. For instance, in a medical diagnosis system, rough set theory can pinpoint the most relevant symptoms and test results, classify patients into diagnostic categories, and generate diagnostic rules from the data. The primary advantages of rough set theory include its independence from preliminary information such as probability distributions or membership functions, its effectiveness in handling uncertainty, and its straightforward, intuitive concepts. Overall, rough set theory is a powerful tool for dealing with imprecise and uncertain information, making it invaluable in various domains such as data analysis, machine learning, and decision-making. Several researchers [50,51] have significantly contributed to the development and application of rough set theory since its inception by Zdzisław Pawlak. Their work spans various domains, including data mining, machine learning, and decision support systems. These researchers have explored feature selection, rule generation, and the handling of uncertainty in data. Their contributions have enhanced the theory's robustness and expanded its practical applications. As a result, rough set theory continues to evolve and find new uses in diverse fields. The q-SFS is acknowledged as the most effective way of dealing with uncertainty and ambiguity. This advanced mathematical framework improves the capacity to tackle these difficulties more effectively than traditional fuzzy sets. The q-SFS, which includes an extra parameter, gives better flexibility and accuracy in expressing complicated decision-making scenarios. This method is especially useful when data is confusing or partial, providing a reliable result. As a result, it has earned a reputation for being exceptionally adept in solving complicated issues in a variety of sectors. The integration of fuzzy logic with rough set theory has been significantly advanced by the theories of IFRS [52], PyFRS [53], and q-ROFRS [54,55]. By extending the traditional rough set structure, these sophisticated models help data analysts more effectively handle ambiguity and imprecision. By combining intuitionistic fuzzy sets with rough sets, IFRS enhances its capacity to manage ambiguity and hesitation. PyFRS allows for a more comprehensive depiction of uncertainty by incorporating Pythagorean fuzzy sets. Similarly, to provide even more flexibility and precision, q-ROFRS makes use of q-rung orthopair fuzzy sets. These developments highlight how rough set theory is always changing and how it can be applied to intricate decision-making procedures. The examination of voting may be conducted using a theoretical framework known as the PFRS. This approach successfully integrates the ideas of picture fuzzy sets with rough set theory, resulting in a strong technique for dealing with the inherent uncertainty and ambiguity in voting scenarios. Despite having lower and upper approximations expressed as $\left(\underline{\zeta_{A}}+\underline{\eta_{A}}+\underline{\xi_{A}}\;\right)\in \left[0,1\right]$ and ($\bar{\zeta_{A}}+\bar{\eta_{A}}+\bar{\xi_{A}}\;)\in \left[0,1\right]$, there are inherent limitations. These limitations arise from the necessity that the combined values of the lower and upper approximations must fall within the [0, 1] range. Immediate and urgent action is needed to fully resolve this issue and ensure the framework's effectiveness and accuracy. However, when decision-makers are presented with information in the form of SFRS, which includes both lower and upper approximations, such as {(0.7,0.8,0.9),(0.9,0.8,0.7)}, the combined values of these approximations frequently exceed the [0, 1] interval. This disparity poses a serious problem because it might cause errors and inconsistencies while making decisions. To solve this issue, a detailed investigation of the theoretical underpinnings is required, as well as the development of improved methodologies to assure the validity and dependability of the approximations within the prescribed period. This project is critical for successfully implementing the PFRS framework in real-world voting and decision-making contexts. The numbers (0⩽̸0.7^2^+0.8^2^+0.9^2^⩽̸1) and (0⩽̸0.9^2^+0.8^2^+0.7^2^⩽̸1) are not appropriate for the SFRS framework, indicating a restriction in its use. In their 2023 research paper, Azim et al. [56] presented the notion of q-SFRS as a solution to this problem. Information with lower and upper approximations can be represented using the q-SFRS framework as follows: {(0.7,0.8,0.9),(0.9,0.8,0.7)}. This ensures that the total values of these approximations stay within the interval [0,1]. This change overcomes the prior limits, increasing the SFRS framework's applicability and accuracy in decision-making processes. This fuzzy set combines the strengths of rough sets and q-SFS. Our study presents a realistic decision-making framework based on q-SFRS, considerably adding to the field's current body of knowledge. The q-SFRS framework employs three distinct parameters for lower and upper approximations, enhancing its flexibility and accuracy. The primary goal of this project is to advance future research by developing new aggregation operators and defuzzification procedures.A detailed examination demonstrates that q-SFRSs hold significant potential as a new concept, opening up several options for future research and practical applications. This breakthrough shows that q-SFRSs might play a critical role in addressing difficult decision-making challenges, enabling the development of more sophisticated and effective solutions across a variety of disciplines.

### 1.2 Research questions behind the combination of q-spherical fuzzy sets and rough sets

The combination of q-SFSs with rough sets may introduce additional complexity, but this complexity is justified by the enhanced ability to model real-world problems involving intricate uncertainties and imprecisions. This relevance is particularly evident in fields requiring high precision and reliability in decision-making under uncertainty. Therefore, the practical necessity of this model lies in its superior capability to handle complex, uncertain, and imprecise data, making it a valuable tool despite its complexity. Traditionally, fuzzy sets allow for partial membership, which is useful for representing uncertainty in a single dimension. The extended q-SFSs introduce a parameter q, which can model more complex uncertainties by incorporating higher-order hesitancy and ambiguity in membership degrees. This makes them more versatile in real-world applications where uncertainty is not merely binary or linear. On the other hand, rough sets are adept at dealing with vagueness by approximating sets with a pair of lower and upper bounds, which is useful when precise boundary information is unavailable. Combining q-SFSs with rough sets leverages the strengths of both approaches. The q-SFS provides a nuanced representation of membership degrees, while the rough set theory deals effectively with boundary vagueness. This hybrid model can thus capture more complex patterns and relationships in data that are not possible with either model alone. For instance, in decision-making systems where data is both ambiguous (fuzzy) and incomplete (rough), this combined approach ensures more accurate and reliable outcomes. Autonomous vehicles rely heavily on sensor data (such as LIDAR, radar, and cameras) to navigate. This data often contains uncertainties and imprecisions due to various factors like weather conditions, lighting, and occlusions. The q-spherical fuzzy sets can model the ambiguous nature of sensor readings more effectively, while rough sets can handle incomplete information, providing a more robust interpretation of the environment. Navigation decisions in autonomous vehicles require precise handling of dynamic and uncertain environments. The combination of q-spherical fuzzy sets and rough sets allows for better modeling of uncertainties related to obstacles, traffic conditions, and road layouts, leading to safer and more reliable decision-making processes. Autonomous vehicles need to continuously plan and re-plan their paths to avoid obstacles and navigate efficiently. The hybrid model enhances the vehicle's ability to generate optimal paths by accurately assessing the uncertainties in the environment and the vehicle's position, ensuring smooth and safe navigation even in complex and unpredictable scenarios. While the combination does increase the mathematical complexity, the practical benefits often justify this complexity. The nuanced modeling of uncertainty leads to more accurate and reliable decision-making, which is crucial in critical applications like autonomous driving. The hybrid model allows for a more comprehensive analysis of data, capturing subtleties that might be missed with simpler models. Such models can adapt to a wide range of applications, providing flexibility in modeling different types of uncertainties. The combination of q-SFSs with rough sets may introduce additional complexity, but this complexity is justified by the enhanced ability to model real-world problems involving intricate uncertainties and imprecisions. This relevance is particularly evident in autonomous navigation systems, where precise interpretation of sensor data, robust decision-making, and reliable path planning are crucial for safety and efficiency. Therefore, the practical necessity of this model lies in its superior capability to handle complex, uncertain, and imprecise data, making it a valuable tool despite its complexity.

### 1.3 Gap before establishing the proposed operators

The preceding gap in the domain of q-SFR sets may be briefly expressed as follows:

Traditional fuzzy sets helped control uncertainty, but they struggled to absorb complicated information and effectively express decision-makers' preferences. This constraint created a gap in decision-making processes, especially when dealing with complex and unexpected data that required more efficient treatment. The introduction of q-SFR sets addressed this gap by improving the ability of conventional fuzzy sets to handle complicated information. However, a critical necessity remained for sophisticated aggregation operators capable of negotiating the intricacies of q-SFR sets while accurately conveying decision-makers' preferences and uncertainties. Einstein's operations and operators, such as q-SFREWA, q-SFREOWA, and q-SFREHWA, were designed to fill the current gap. These operators combine the advantages of q-SFR sets with Einstein aggregation techniques, allowing for more accurate and adaptive decision-making in complex and unpredictable circumstances. They provide powerful and comprehensive ways of gathering information and accurately reflecting decision-makers' preferences. In essence, before the advent of Einstein's operations and operators, there was a lack of sophisticated aggregation approaches capable of processing complicated information and properly representing decision-makers' preferences. Einstein's operators effectively bridge this gap by incorporating powerful and thorough aggregation methods into decision-making procedures.

### 1.4 Motivations for the proposed operators

The motivations behind the introduction of q-SFRSs and their associated aggregation operators, such as q-SFREWA, q-SFREOWA, and q-SFREHWA, can be articulated as follows.

1. q-spherical fuzzy rough sets: These sets are designed to handle and represent complex and ambiguous information more effectively than traditional fuzzy sets. They offer a versatile and precise framework for decision-making in unpredictable and complex scenarios. This enhanced flexibility allows decision-makers to navigate uncertainty with greater accuracy and adaptability, making it invaluable across various contexts and applications
2. q-spherical fuzzy rough Einstein weighted averaging: This operator calculates a weighted average by integrating the benefits of q-SFRSs with Einstein aggregation. The aim is to enhance the handling of complex and ambiguous data, leading to more accurate and adaptable decision-making.
3. q-spherical fuzzy rough Einstein ordered weighted averaging: By applying the concept of ordered weighted averages to q-SFRSs, this operator enables decision-makers to account for the importance of criteria and their levels of optimism or pessimism. The goal is to capture decision-makers' preferences and uncertainties comprehensively.
4. q-spherical fuzzy rough Einstein weighted averaging This hybrid operator combines both weighted and hybrid averages, considering the weights and hybrid distances between q-spherical fuzzy rough sets. The aim is to offer a robust and precise aggregation method that reflects the influence of both weights and hybrid distances accurately.

Integrating q-SFR sets into various decision support systems has proven to significantly enhance the robustness and efficiency of these systems. The proposed structure fulfills the need for more advanced aggregation operators capable of handling the complexities of q-SFR sets while accurately reflecting decision-makers' preferences and uncertainties.

### 1.5 Significance

1. The proposed study on q-spherical fuzzy rough sets (q-SFRSs) and their associated Einstein aggregation operators is significant due to its innovative approach to handling complex, ambiguous, and uncertain information. This study addresses several critical gaps in the current state of fuzzy set theory and rough set theory, providing a comprehensive framework that enhances decision-making processes across various fields. Below are the key aspects highlighting the significance of this research:
2. Traditional fuzzy sets and rough sets are limited in their ability to fully capture the nuances of complex uncertainty. q-SFRSs introduce an additional parameter, q, which allows for a more nuanced representation of membership, non-membership, and hesitancy degrees, thus offering a richer framework for modeling uncertainty.
3. By combining the strengths of fuzzy sets and rough sets, q-SFRSs improve the accuracy of decision-making processes. This hybrid approach provides a more detailed and accurate representation of data, leading to better-informed decisions, especially in environments characterized by high uncertainty and complexity.
4. The introduction of Einstein aggregation operators, such as q-SFREWA, q-SFREOWA, and q-SFREHWA, provides sophisticated tools for aggregating q-SFRNs. These operators leverage the unique properties of Einstein operations to offer more precise and reliable aggregation methods, addressing the limitations of traditional aggregation techniques.
5. The proposed framework applies to various domains, including autonomous systems, medical diagnosis, and multi-attribute decision-making (MADM). The ability to handle complex and uncertain data makes q-SFRSs particularly valuable in fields requiring high precision and reliability.

This work lays the groundwork for further investigations into the fields of rough set theory and fuzzy set theory. It opens doors for additional research and development in linked sectors by presenting cutting-edge ideas and techniques.

### 1.6 Objectives

The main objectives of the study are outlined as follows.

1. To formally define and develop the mathematical framework for q-SFRSs. This involves establishing the basic properties, operational laws, and algebraic structures of q-SFRSs, ensuring they effectively combine the features of both fuzzy sets and rough sets.
2. To design and define new Einstein aggregation operators specifically tailored for q-SFRNs. This includes developing operators such as q-SFREWA (q-spherical fuzzy rough Einstein weighted averaging), q-SFREOWA (q-spherical fuzzy rough Einstein ordered weighted averaging), and q-SFREHWA (q-spherical fuzzy rough Einstein hybrid weighted averaging). These operators will leverage the properties of Einstein operations to handle the complexities of q-SFRSs.
3. To conduct a comparative analysis of the proposed Einstein aggregation operators with existing methods. This involves evaluating the performance of the new operators against traditional aggregation techniques and other advanced methods like Dombi and Archimedean aggregation operators. Metrics for comparison will include accuracy, robustness, and ability to handle extreme values and outliers.
4. There will be case studies in domains including autonomous car navigation, medical diagnosis, and multi-attribute group decision-making (MAGDM) to illustrate the practical usefulness of q-SFRSs and associated aggregation operators in real-world decision-making scenarios. These examples will serve as proof of the suggested framework's efficacy.
5. To perform sensitivity and robustness analysis on the proposed methods. This involves assessing how changes in the input data and parameters affect the performance of the q-SFRS-based decision-making framework. The goal is to ensure that the proposed methods are reliable and robust under varying conditions.

The work seeks to make a substantial addition to the domains of fuzzy set theory, rough set theory, and uncertainty-based decision-making. The suggested q-SFRSs and Einstein aggregation operators offer enhanced tools for modeling and handling complicated, ambiguous, and uncertain data, opening the way for more accurate and trustworthy decision-making processes.

**Fig. 1:**
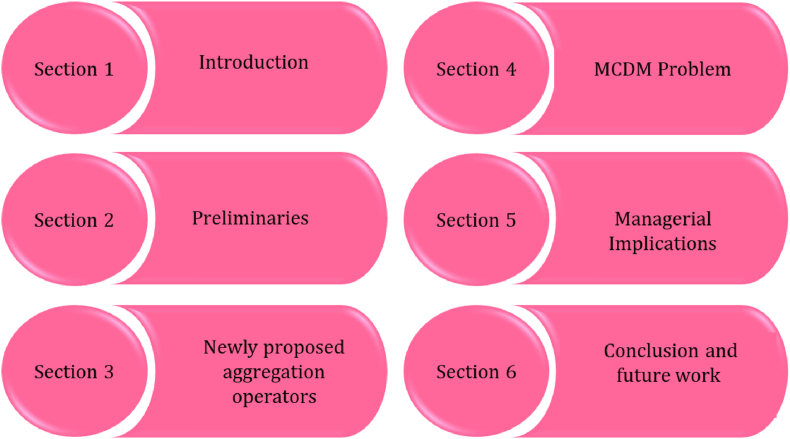
Structure of the research article.

The paper is structured as follows: Section 2 provides a comprehensive review of several topics, including PFS, SFS, q- SFS, RS, and q- SFRS, laying the foundation for subsequent sections. Section 3 discusses the “main results (aggregation operators based on q-SFRS)," exploring three distinct aggregation operators based on Einstein's operations. In Section 4, the “multi-criteria group decision-making approach” is introduced, focusing on the development of a multi-criteria group decision-making framework. Section 5 examines the managerial implications of the proposed q-SFRSs and Einstein aggregation operators for decision-making processes, including comparative and sensitivity analyses to highlight strengths and weaknesses. Section 6, titled “Conclusion and Future Directions,” summarizes the key findings, underscores the study's significance, and proposes numerous avenues for future research. Fig. 1 illustrates the overall structure of the article.

## 2 Preliminaries

In this section, we will explore a diverse array of mathematical concepts, starting with a comprehensive review of PFS, SFS, q-SFS, and RS. Each of these theories plays a crucial role in addressing different facets of uncertainty and imprecision in data analysis and decision-making.

Definition 1 [20] Let X be a non-empty set. A picture fuzzy set A in X is defined by a membership function $\zeta_{A}$, a neutral membership function $\eta_{A}$, and a non-membership function $\xi_{A}$. The mathematical characterization of a PFS is as follows:

$$A=\{\left\langle x,\zeta_{A}\left(x\right),\eta_{A}\left(x\right),\xi_{A}\left(x\right)\right\rangle :x∊X\}$$

Where:

$\zeta_{A}$: X → [0, 1] represents the degree of membership of each element in X to the set A.

$\eta_{A}$: X → [0, 1] represents the degree of neutral membership of each element in X to the set A.

$\xi_{A}$: X → [0, 1] represents the degree of non-membership of each element in X to the set A.

These functions must satisfy the following condition for every element x ∈ X:

$$0\leq ∙\zeta_{A}\left(x\right)+\eta_{A}\left(x\right)+\xi_{A}\left(x\right)\leq 1\text{.}$$

Definition 2 [22] Consider a non-empty set X. A spherical fuzzy set A in X is characterized by a membership function $\zeta_{A}$, a neutral membership function $\eta_{A}$, and a non-membership function $\xi_{A}$. The mathematical formulation of a spherical fuzzy set is provided below:

$$A=\{\left\langle x,\zeta_{A}\left(x\right),\eta_{A}\left(x\right),\xi_{A}\left(x\right)\right\rangle :x∊X\}$$

Where:

$\zeta_{A}$: X → [0, 1] represents the degree of membership of each element in X to the set A.

$\eta_{A}$: X → [0, 1] represents the degree of neutral membership of each element in X to the set A.

$\xi_{A}$: X → [0, 1] represents the degree of non-membership of each element in X to the set A.

These functions must satisfy the following condition for every element x ∈ X:

$${0\leq \left(\zeta_{A}\left(x\right)\right)}^{2}+{\left(\eta_{A}\left(x\right)\right)}^{2}+{\left(\xi_{A}\left(x\right)\right)}^{2}\leq 1\text{.}$$

Definition 3 [47] Let X be a non-empty set. A q-SFS A in X is characterized by the membership function $\zeta_{A}$, the neutral membership function $\eta_{A}$, and the non-membership function $\xi_{A}$. The mathematical formulation of a q-SFS is provided as follows:

$$A=\{\left\langle x,\zeta_{A}\left(x\right),\eta_{A}\left(x\right),\xi_{A}\left(x\right)\right\rangle :x∊X\}$$

Where:

$\zeta_{A}$: X → [0, 1] represents the degree of membership of each element in X to the set A.

$\eta_{A}$: X → [0, 1] represents the degree of neutral membership of each element in X to the set A.

$\xi_{A}$: X → [0, 1] represents the degree of non-membership of each element in X to the set A.

**Fig. 2:**
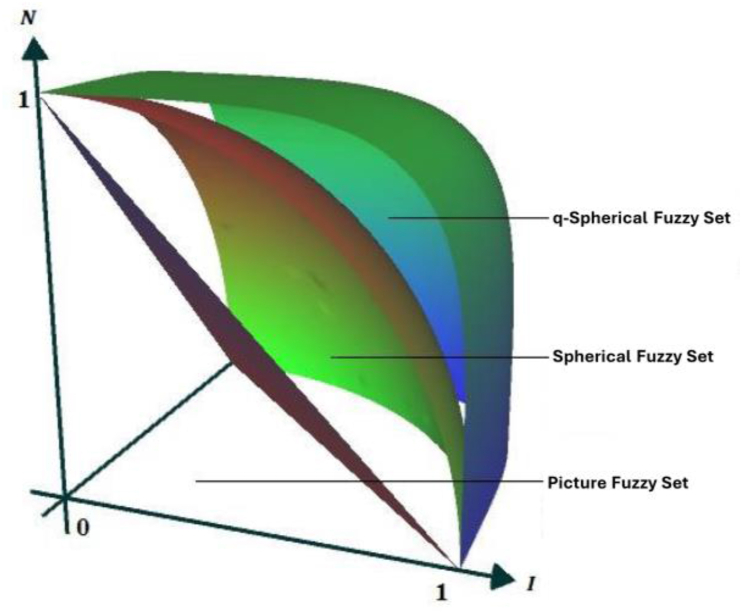
Visual comparison between the picture fuzzy set, spherical fuzzy set, and q-spherical fuzzy set in three dimensions.

The following requirement must be met by these functions for any element x in X: ${0\leq \left(\zeta_{A}\left(x\right)\right)}^{q}+{\left(\eta_{A}\left(x\right)\right)}^{q}+{\left(\xi_{A}\left(x\right)\right)}^{q}\leq 1\;\text{for}\;\text{all}\;q\geq 1\text{.}$ The graphical depiction of PFS, SFS, and q-SFS differences in three-dimensional space is shown in Fig. 2.

Definition 4 [56] Let $G_{1}$ and $G_{2}$ are non-empty sets. Typically, $G_{1}$ is the universe of discourse, and $G_{2}$ is a set of attributes or conditions. Let R ⊆ $G_{1}\times G_{2}$ be a binary relation between elements of $G_{1}$ and $G_{2}\text{.}$ The lower and upper set approximations for a subset X ⊆ $G_{1}$ and ⊆ $G_{2}$ are as follows:

$$\left(\begin{array}{c} \underline{R}\left(A\right)=\left\{x∊G_{1}:{\left[x\right]}_{A}\subseteq G\right\}\\ \bar{R}\left(A\right)=\left\{x∊G_{1}:{\left[x\right]}_{A}⋂G\neq \phi \right\} \end{array}\right)$$

Here, ${\left[x\right]}_{A}$ represents the idea of indiscernibility within the context of these subsets.

The pair $\left(\underline{R}\left(A\right),\bar{R}\left(A\right)\right)$ is sometimes referred to as a rough set.

Definition 5 [56] A q-SF relation R is a q-SF subset of $G_{1}\times G_{2}$ and is defined as follows:

$$R=\left\{\left\langle \left(p,x\right):\zeta_{R}\left(p,x\right),{\;\eta }_{R}\left(p,x\right),{\;\xi }_{R}\left(p,x\right)\right\rangle :\left({{\left(\zeta_{R}\left(p,x\right)\right)}^{q}+\left({\;\eta }_{R}\left(p,x\right)\right)}^{q}+{\left({\;\xi }_{R}\left(p,x\right)\right)}^{q}\right)\leq 1:\forall \;p\in G_{1},x\in G_{2}\right\}\text{,}$$

where $\zeta_{R}:X\to \left[0,1\right]$, ${\;\eta }_{R}:X\to \left[0,1\right]\;\text{and}\;\xi_{R}:X\to \left[0,1\right]$.

Definition 6 [56] Consider the universal set $G_{1}$ and the set of attributes $G_{2}$. Let R be a q-SF relation from $G_{1}$ to $G_{2}$. The q-SF approximation space is defined as the triplet $\left(G_{1},G_{2},R\right)$. The lower and upper approximation spaces of a given element p with respect to the approximation space $\left(G_{1},G_{2},R\right)$ are defined and elaborated as follows:

$$A=\left(\underline{A},\bar{A}\right)=\left\{p,\left(\begin{array}{c} {\underline{\zeta }}_{A}\left(p\right),{\underline{\zeta }}_{A}\left(p\right),{\underline{\zeta }}_{A}\left(p\right),\\ {\bar{\zeta }}_{A}\left(p\right),{\bar{\zeta }}_{A}\left(p\right),{\bar{\zeta }}_{A}\left(p\right) \end{array}\right):p\in G_{1}\right\}$$

Where,

$${\underline{\zeta }}_{A}\left(p\right)={⋀}_{x\in G_{2}}\left\{\zeta_{R}\left(p,x\right)⋀\zeta_{A}\left(x\right)\right\}\text{,}$$

$${\underline{\eta }}_{A}\left(p\right)={⋁}_{x\in G_{2}}\left\{\eta_{R}\left(p,x\right)⋁\eta_{A}\left(x\right)\right\}\text{,}$$

$${\underline{\xi }}_{A}\left(p\right)={⋁}_{x\in G_{2}}\left\{\xi_{R}\left(p,x\right)⋁\xi_{A}\left(x\right)\right\}\text{,}$$

$${\bar{\zeta }}_{A}\left(p\right)={⋁}_{x\in G_{2}}\left\{\zeta_{R}\left(p,x\right)⋁\zeta_{A}\left(x\right)\right\}\text{,}$$

$${\bar{\eta }}_{A}\left(p\right)={⋀}_{x\in U_{2}}\left\{\eta_{R}\left(p,x\right)⋀\eta_{A}\left(x\right)\right\}\text{,}$$

$${\bar{\xi }}_{A}\left(p\right)={⋀}_{x\in G_{2}}\left\{\xi_{R}\left(p,x\right)⋀\xi_{A}\left(x\right)\right\}\text{,}$$

with the condition that $\left(0\leq {{\underline{\zeta }}_{A}}^{q}\left(p\right)+{{\underline{\eta }}_{A}}^{q}\left(p\right)+{{\underline{\xi }}_{A}}^{q}\left(p\right)\leq 1\right)$ and $\left(0\leq {{\bar{\zeta }}_{A}}^{q}\left(p\right)+{{\bar{\eta }}_{A}}^{q}\left(p\right)+{{\bar{\xi }}_{A}}^{q}\left(p\right)\leq 1\right)$.

**Fig. 3:**
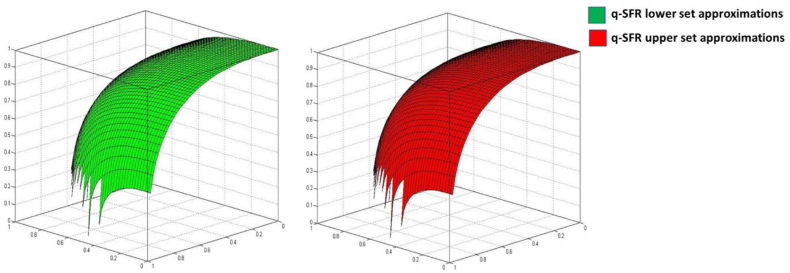
Three-dimensional graphical representation of a q-SFRS.

A q-SFRS is characterized by a pair of q-SFSs with distinct lower and upper set approximations. This concept is denoted as $A=\left(\underline{A},\bar{A}\right)$ and referred to as a q-SFR number. The collection of all q-SFR numbers is represented as $A_{i}$. A q-SFRS comprises a pair of q-SFSs where $\underline{A}$ differs from $\bar{A}$. Fig. 3 illustrates the three-dimensional graphical representation of a q-SFRS.

Definition 7 [56] Consider $A=\left(\underline{\zeta },\underline{\eta },\underline{\xi },\bar{\zeta },\bar{\eta },\bar{\xi }\right)$, $A_{1}=\left({\underline{\zeta }}_{1},{\underline{\eta }}_{1},{\underline{\xi }}_{1},{\bar{\zeta }}_{1},{\bar{\eta }}_{1},{\bar{\xi }}_{1}\right)$ and $A_{2}=\left({\underline{\zeta }}_{2},{\underline{\eta }}_{2},{\underline{\xi }}_{2},{\bar{\zeta }}_{2},{\bar{\eta }}_{2},{\bar{\xi }}_{2}\right)$ be any three q-SFRNs, and ω>0, then,

1. $A_{1}⊕A_{2}=\left\langle \begin{array}{l} \sqrt[{q}]{{\underline{\zeta }}_{1}^{q}+{\underline{\zeta }}_{2}^{q}-{\underline{\zeta }}_{1}^{q}{\underline{\zeta }}_{2}^{q}},{\;\underline{\eta }}_{1}^{q}{\underline{\eta }}_{2}^{q},\sqrt[{q}]{\left(1-{\underline{\zeta }}_{2}^{q}{\underline{\xi }}_{1}^{q}+1-{\underline{\zeta }}_{1}^{q}{\underline{\xi }}_{2}^{q}\right)-{\underline{\xi }}_{1}^{q}{\underline{\xi }}_{2}^{q}},\\ \sqrt[{q}]{{\bar{\zeta }}_{1}^{q}+{\bar{\zeta }}_{2}^{q}-{\bar{\zeta }}_{1}^{q}{\bar{\zeta }}_{2}^{q}},{\;\bar{\eta }}_{1}^{q}{\bar{\eta }}_{2}^{q},\sqrt[{q}]{\left(1-{\bar{\zeta }}_{2}^{q}{\bar{\xi }}_{1}^{q}+1-{\bar{\zeta }}_{1}^{q}{\bar{\xi }}_{2}^{q}\right)-{\bar{\xi }}_{1}^{q}{\bar{\xi }}_{2}^{q}} \end{array}\right\rangle$,
2. $A_{1}⊗A_{2}=\left\langle \begin{array}{l} {\;\underline{\zeta }}_{1}^{q}{\underline{\zeta }}_{2}^{q},\sqrt[{q}]{{\underline{\eta }}_{1}^{q}+{\underline{\eta }}_{2}^{q}-{\underline{\eta }}_{1}^{q}{\underline{\eta }}_{2}^{q}},\sqrt[{q}]{\left(1-{\underline{\eta }}_{2}^{q}{\underline{\xi }}_{1}^{q}+1-{\underline{\eta }}_{1}^{q}{\underline{\xi }}_{2}^{q}\right)-{\underline{\xi }}_{1}^{q}{\underline{\xi }}_{2}^{q}},\\ {\;\bar{\zeta }}_{1}^{q}{\bar{\zeta }}_{2}^{q},\sqrt[{q}]{{\bar{\eta }}_{1}^{q}+{\bar{\eta }}_{2}^{q}-{\bar{\eta }}_{1}^{q}{\bar{\eta }}_{2}^{q}},\sqrt[{q}]{\left(1-{\bar{\eta }}_{2}^{q}{\bar{\xi }}_{1}^{q}+1-{\bar{\eta }}_{1}^{q}{\bar{\xi }}_{2}^{q}\right)-{\bar{\xi }}_{1}^{q}{\bar{\xi }}_{2}^{q}} \end{array}\right\rangle$.
3. $A^{\omega }=\left\langle \begin{array}{l} {\underline{\zeta }}^{\omega },\sqrt[{q}]{{1-\left(1-{\underline{\eta }}^{q}\right)}^{\omega }},\sqrt[{q}]{{\left(1-{\underline{\eta }}^{q}\right)}^{\omega }-{\left(1-{\underline{\eta }}^{q}-{\underline{\xi }}^{q}\right)}^{\omega }},\\ {\bar{\zeta }}^{\omega },\sqrt[{q}]{{1-\left(1-{\bar{\eta }}^{q}\right)}^{\omega }},\sqrt[{q}]{{\left(1-{\bar{\eta }}^{q}\right)}^{\omega }-{\left(1-{\bar{\eta }}^{q}-{\bar{\xi }}^{q}\right)}^{\omega }} \end{array}\right\rangle$,
4. $\omega A=\left\langle \begin{array}{l} \sqrt[{q}]{{1-\left(1-{\underline{\zeta }}^{q}\right)}^{\omega }},{\underline{\eta }}^{\omega },\sqrt[{q}]{{\left(1-{\underline{\zeta }}^{q}-{\underline{\xi }}^{q}\right)}^{\omega }},\\ \sqrt[{q}]{{1-\left(1-{\bar{\zeta }}^{q}\right)}^{\omega }},{\bar{\eta }}^{\omega },\sqrt[{q}]{{\left(1-{\bar{\zeta }}^{q}-{\bar{\xi }}^{q}\right)}^{\omega }} \end{array}\right\rangle$,
5. $A_{1}=A_{2}$ if and only if ${\underline{\zeta }}_{1}={\underline{\zeta }}_{2},{\underline{\eta }}_{1}={\underline{\eta }}_{2}$ ${\underline{\xi }}_{1}={\underline{\xi }}_{2}$ and ${\bar{\zeta }}_{1}={\bar{\zeta }}_{2},{\bar{\eta }}_{1}={\bar{\eta }}_{2}$ ${\bar{\xi }}_{1}={\bar{\xi }}_{2}$.

Definition 8 [56] Let $A=\left(\underline{\zeta },\underline{\eta },\underline{\xi },\bar{\zeta },\bar{\eta },\bar{\xi }\right)$ denote a q-SFRN. A specific formula is employed to compute the score value Sco(A) for A. This score value numerically represents the q-SFRN, facilitating comparison and analysis. The formula for calculating the score value is provided below:

$$Sco\left(A\right)=\frac{{2+\left(\underline{\zeta }\right)}^{q}+{\left(\bar{\zeta }\right)}^{q}-{\left(\underline{\eta }\right)}^{q}-{\left(\bar{\eta }\right)}^{q}-{\left(\underline{\xi }\right)}^{q}-{\left(\bar{\xi }\right)}^{q}}{3}$$

Where 0≤Sco(A)≤1. This formula allows for the determination of the score value of the q-SFRN.

Definition 9 [56] Let $A=\left(\underline{\zeta },\underline{\eta },\underline{\xi },\bar{\zeta },\bar{\eta },\bar{\xi }\right)$) denote a q-SFRN. A specific formula is employed to determine the accuracy of A, denoted as Acc(A). To calculate the accuracy of A, use the formula provided below:

$$Acc\left(A\right)=\frac{{\left(\underline{\zeta }\right)}^{q}+{\left(\underline{\zeta }\right)}^{q}-{\left(\underline{\xi }\right)}^{q}-{\left(\bar{\xi }\right)}^{q}}{2}$$

Where −1≤Acc(A)≤1. This formula is used to calculate the accuracy value of the q-SFRN.

Definition 10 [56] Let $A_{1}=\left({\underline{\zeta }}_{1},{\underline{\eta }}_{1},{\underline{\xi }}_{1},{\bar{\zeta }}_{1},{\bar{\eta }}_{1},{\bar{\xi }}_{1}\right)$ and $A_{2}=\left({\underline{\zeta }}_{2},{\underline{\eta }}_{2},{\underline{\xi }}_{2},{\bar{\zeta }}_{2},{\bar{\eta }}_{2},{\bar{\xi }}_{2}\right)$ are two q-SFRNs, then

1. If $Sco\left(A_{1}\right)<Sco\left(A_{2}\right)$ then $A_{1}<A_{2}$,
2. If $Sco\left(A_{1}\right)>Sco\left(A_{2}\right)$ then $A_{1}>A_{2}$,
3. If $Sco\left(A_{1}\right)=Sco\left(A_{2}\right)$ then

• If $Acc\left(A_{1}\right)<Acc\left(A_{2}\right)$ then $A_{1}>A_{2}$,
• If $Acc\left(A_{1}\right)>Acc\left(A_{2}\right)$ then $A_{1}>A_{2}$,
• If $Acc\left(A_{1}\right)=Acc\left(A_{2}\right)$ then $A_{1}=A_{2}$.

Definition 11 [56] Consider $A=\left(\underline{\zeta },\underline{\eta },\underline{\xi },\bar{\zeta },\bar{\eta },\bar{\xi }\right)$, $A_{1}=\left({\underline{\zeta }}_{1},{\underline{\eta }}_{1},{\underline{\xi }}_{1},{\bar{\zeta }}_{1},{\bar{\eta }}_{1},{\bar{\xi }}_{1}\right)$ and $A_{2}=\left({\underline{\zeta }}_{2},{\underline{\eta }}_{2},{\underline{\xi }}_{2},{\bar{\zeta }}_{2},{\bar{\eta }}_{2},{\bar{\xi }}_{2}\right)$ as any three q-SFRNs. Let ω, $\omega_{1}$ and $\omega_{2}$ be any positive integers. Then the following properties hold:

1. $A_{1}⊕A_{2}=A_{2}⊕A_{1}$,
2. $A_{1}⊗A_{2}=A_{2}⊗A_{1}$.
3. $\omega \left(A_{1}⊕A_{2}\right)=\omega A_{1}⊕\omega A_{2}$,
4. $\omega_{1}A⊕\omega_{2}A=\left(\omega_{1}+\omega_{2}\right)A$,
5. ${\left(A_{1}⊗A_{2}\right)}^{\omega }=A_{1}^{\omega }⊗A_{2}^{\omega }$,
6. $A^{\omega_{1}}⊗A^{\omega_{2}}=A^{\omega_{1}+\omega_{2}}$.

Definition 12 Using the t-norm Ҭ, t-conorm S, Einstein's operations are as follows:

$${Ҭ}_{Ḛ}\left(X,Y\right)=\frac{X+Y}{1+\left(1-X\right)\left(1-Y\right)}$$

$$S_{Ḛ}\left(X,Y\right)=\frac{X+Y}{1+XY}$$

## 3 Proposed operational laws for q-SFRSs

In this section, we develop a comprehensive set of operational laws within the framework of q-SFRSs. This involves defining and exploring various mathematical properties and operations that apply to these sets. Using these established operational laws, we provide a diversified collection of aggregation operators designed specifically for the integration of q-SFR information. This technique greatly increases the flexibility and accuracy of aggregation procedures within the stated framework, resulting in higher decision-making efficacy in complex settings. The development of these operational laws and aggregation operators significantly enhances the capacity to process and analyze q-SFR information. By providing a robust framework for the integration and manipulation of such information, these tools improve the flexibility and accuracy of aggregation procedures. Consequently, they offer greater decision-making efficacy in complex settings, making them valuable for applications in various fields requiring nuanced handling of uncertainty and vagueness.

### 3.1 Operational laws

Definition 13 Consider $A=\left(\underline{\zeta },\underline{\eta },\underline{\xi },\bar{\zeta },\bar{\eta },\bar{\xi }\right)$, $A_{1}=\left({\underline{\zeta }}_{1},{\underline{\eta }}_{1},{\underline{\xi }}_{1},{\bar{\zeta }}_{1},{\bar{\eta }}_{1},{\bar{\xi }}_{1}\right)$ and $A_{2}=\left({\underline{\zeta }}_{2},{\underline{\eta }}_{2},{\underline{\xi }}_{2},{\bar{\zeta }}_{2},{\bar{\eta }}_{2},{\bar{\xi }}_{2}\right)$ as any three q-SFRNs, where ω>0, the essential Einstein's operations for q-SFRNs based on t-norm and t-conorm are presented as follows:

(i). $A_{1}{⊕}_{Ḛ}A_{2}=\left[\left\langle \sqrt[{q}]{\frac{{\underline{\zeta }}_{1}^{q}+{\underline{\zeta }}_{2}^{q}}{1+{\underline{\zeta }}_{1}^{q}.{\underline{\zeta }}_{2}^{q}}},\sqrt[{q}]{\frac{{\underline{\eta }}_{1}^{q}.{\underline{\eta }}_{1}^{q}}{1+\left(1-{\underline{\eta }}_{1}^{q}\right).\left(1-{\underline{\eta }}_{2}^{q}\right)}},\sqrt[{q}]{\frac{{\underline{\xi }}_{1}^{q}.{\underline{\xi }}_{2}^{q}}{1+\left(1-{\underline{\xi }}_{1}^{q}\right).\left(1-{\underline{\xi }}_{2}^{q}\right)}},\sqrt[{q}]{\frac{{\bar{\zeta }}_{1}^{q}+{\bar{\zeta }}_{2}^{q}}{1+{\bar{\zeta }}_{1}^{q}.{\bar{\zeta }}_{2}^{q}}},\sqrt[{q}]{\frac{{\bar{\eta }}_{1}^{q}.{\bar{\eta }}_{2}^{q}}{1+\left(1-{\bar{\eta }}_{1}^{q}\right).\left(1-{\bar{\eta }}_{2}^{q}\right)}},\sqrt[{q}]{\frac{{\bar{\xi }}_{1}^{q}.{\bar{\xi }}_{2}^{q}}{1+\left(1-{\bar{\xi }}_{1}^{q}\right).\left(1-{\bar{\xi }}_{2}^{q}\right)}}\right\rangle \right]$.
(ii). $A_{1}{⊗}_{Ḛ}A_{2}=\left[\left\langle \sqrt[{q}]{\frac{{\underline{\zeta }}_{1}^{q}.{\underline{\zeta }}_{2}^{q}}{1+\left(1-{\underline{\zeta }}_{1}^{q}\right).\left(1-{\underline{\zeta }}_{2}^{q}\right)}},\sqrt[{q}]{\frac{{\underline{\eta }}_{1}^{q}+{\underline{\eta }}_{2}^{q}}{1+{\underline{\eta }}_{1}^{q}.{\underline{\eta }}_{2}^{q}}},\sqrt[{q}]{\frac{{\underline{\xi }}_{1}^{q}+{\underline{\xi }}_{2}^{q}}{1+{\underline{\xi }}_{1}^{q}.{\underline{\xi }}_{2}^{q}}},\sqrt[{q}]{\frac{{\bar{\zeta }}_{1}^{q}.{\bar{\zeta }}_{2}^{q}}{1+\left(1-{\bar{\zeta }}_{1}^{q}\right).\left(1-{\bar{\zeta }}_{2}^{q}\right)}},\sqrt[{q}]{\frac{{\bar{\eta }}_{1}^{q}+{\bar{\eta }}_{2}^{q}}{1+{\bar{\eta }}_{1}^{q}.{\bar{\eta }}_{2}^{q}}},\sqrt[{q}]{\frac{{\bar{\xi }}_{1}^{q}+{\bar{\xi }}_{2}^{q}}{1+{\bar{\xi }}_{1}^{q}.{\bar{\xi }}_{2}^{q}}}\right\rangle \right]$.
(iii). $\omega_{Ḛ}A=\left[\left\langle \sqrt[{q}]{\frac{{\left(1+{\underline{\zeta }}^{q}\right)}^{\omega }-{\left(1-\underline{{\underline{\zeta }}^{q}}\right)}^{\omega }}{{\left(1+{\underline{\zeta }}^{q}\right)}^{\omega }+{\left(1-{\underline{\zeta }}^{q}\right)}^{\omega }}},\sqrt[{q}]{\frac{2{\left({\underline{\eta }}^{q}\right)}^{\omega }}{{\left(2-{\underline{\eta }}^{q}\right)}^{\omega }+{\left({\underline{\eta }}^{q}\right)}^{\omega }}},\sqrt[{q}]{\frac{2{\left({\underline{\xi }}^{q}\right)}^{\omega }}{{\left(2-{\underline{\xi }}^{q}\right)}^{\omega }+{\left({\underline{\xi }}^{q}\right)}^{\omega }}},\sqrt[{q}]{\frac{{\left(1+{\bar{\zeta }}^{q}\right)}^{\omega }-{\left(1-{\bar{\zeta }}^{q}\right)}^{\omega }}{{\left(1+{\bar{\zeta }}^{q}\right)}^{\omega }+{\left(1-{\bar{\zeta }}^{q}\right)}^{\omega }}},\sqrt[{q}]{\frac{2{\left({\bar{\eta }}^{q}\right)}^{\omega }}{{\left(2-{\bar{\eta }}^{q}\right)}^{\omega }+{\left({\bar{\eta }}^{q}\right)}^{\omega }}},\sqrt[{q}]{\frac{2{\left({\bar{\xi }}^{q}\right)}^{\omega }}{{\left(2-{\bar{\xi }}^{q}\right)}^{\omega }+{\left({\bar{\xi }}^{q}\right)}^{\omega }}}\right\rangle \right]$.
(iv) ${\;A}^{{Ḛ}^{\omega }}=\left[\left\langle \sqrt[{q}]{\frac{2{\left({\underline{\zeta }}^{q}\right)}^{\omega }}{{\left(2-{\underline{\zeta }}^{q}\right)}^{\omega }+{\left({\underline{\zeta }}^{q}\right)}^{\omega }}},\sqrt[{q}]{\frac{{\left(1+{\underline{\eta }}^{q}\right)}^{\omega }-{\left(1-{\underline{\eta }}^{q}\right)}^{\omega }}{{\left(1+{\underline{\eta }}^{q}\right)}^{\omega }+{\left(1-{\underline{\eta }}^{q}\right)}^{\omega }}},\sqrt[{q}]{\frac{{\left(1+{\underline{\xi }}^{q}\right)}^{\omega }-{\left(1-{\underline{\xi }}^{q}\right)}^{\omega }}{{\left(1+{\underline{\xi }}^{q}\right)}^{\omega }+{\left(1-{\underline{\xi }}^{q}\right)}^{\omega }}},\sqrt[{q}]{\frac{2{\left({\bar{\zeta }}^{q}\right)}^{\omega }}{{\left(2-{\bar{\zeta }}^{q}\right)}^{\omega }+{\left({\bar{\zeta }}^{q}\right)}^{\omega }}},\sqrt[{q}]{\frac{{\left(1+{\bar{\eta }}^{q}\right)}^{\omega }-{\left(1-{\bar{\eta }}^{q}\right)}^{\omega }}{{\left(1+{\bar{\eta }}^{q}\right)}^{\omega }+{\left(1-{\bar{\eta }}^{q}\right)}^{\omega }}},\sqrt[{q}]{\frac{{\left(1+{\bar{\xi }}^{q}\right)}^{\omega }-{\left(1-{\bar{\xi }}^{q}\right)}^{\omega }}{{\left(1+{\bar{\xi }}^{q}\right)}^{\omega }+{\left(1-{\bar{\xi }}^{q}\right)}^{\omega }}}\right\rangle \right]$.

Example 1 Let $A_{1}=\left(0.4,0.1,0.3,0.2,0.4,0.3\right)$ , $A_{2}=\left(0.5,0.9,0.5,0.8,0.6,0.2\right)$ and A=(0.4,0.6,0.7,0.5,0.2,0.8) be any three q-SFRNs. if ω=0.5andq=3 then the operational laws defined in Definition 13 can be calculated as:

$$A_{1}{⊕}_{Ḛ}A_{2}=\left[\left\langle \sqrt[{q}]{\frac{{\underline{\zeta }}_{1}^{q}+{\underline{\zeta }}_{2}^{q}}{1+{\underline{\zeta }}_{1}^{q}.{{\underline{\zeta }}_{2}^{q}}^{\_}}},\sqrt[{q}]{\frac{{\underline{\eta }}_{1}^{q}.{\underline{\eta }}_{2}^{q}}{1+\left(1-{\underline{\eta }}_{1}^{q}\right).\left(1-{\underline{\eta }}_{2}^{q}\right)}},\sqrt[{q}]{\frac{{\underline{\xi }}_{1}^{q}.{\underline{\xi }}_{2}^{q}}{1+\left(1-{\underline{\xi }}_{1}^{q}\right).\left(1-{\underline{\xi }}_{2}^{q}\right)}},\sqrt[{q}]{\frac{{\bar{\zeta }}_{1}^{q}+{\bar{\zeta }}_{2}^{q}}{1+{\bar{\zeta }}_{1}^{q}.{\bar{\zeta }}_{2}^{q}}},\sqrt[{q}]{\frac{{\bar{\eta }}_{1}^{q}.{\bar{\eta }}_{2}^{q}}{1+\left(1-{\bar{\eta }}_{1}^{q}\right).\left(1-{\bar{\eta }}_{2}^{q}\right)}},\sqrt[{q}]{\frac{{\bar{\xi }}_{1}^{q}.{\bar{\xi }}_{2}^{q}}{1+\left(1-{\bar{\xi }}_{1}^{q}\right).\left(1-{\bar{\xi }}_{2}^{q}\right)}}\right\rangle \right]$$

=(0.5729,0.0831,0.1222,0.8031,0.1998,0.0479)

$$A_{1}{⊗}_{Ḛ}A_{2}=\left[\left\langle \sqrt[{q}]{\frac{{\underline{\zeta }}_{1}^{q}.{\underline{\zeta }}_{2}^{q}}{1+\left(1-{\underline{\zeta }}_{1}^{q}\right).\left(1-{\underline{\zeta }}_{2}^{q}\right)}},\sqrt[{q}]{\frac{{\underline{\eta }}_{1}^{q}+{\underline{\eta }}_{2}^{q}}{1+{\underline{\eta }}_{1}^{q}.{\underline{\eta }}_{2}^{q}}},\sqrt[{q}]{\frac{{\underline{\xi }}_{1}^{q}+{\underline{\xi }}_{2}^{q}}{1+{\underline{\xi }}_{1}^{q}.{\underline{\xi }}_{2}^{q}}},\sqrt[{q}]{\frac{{\bar{\zeta }}_{1}^{q}.{\bar{\zeta }}_{2}^{q}}{1+\left(1-{\bar{\zeta }}_{1}^{q}\right).\left(1-{\bar{\zeta }}_{2}^{q}\right)}},\sqrt[{q}]{\frac{{\bar{\eta }}_{1}^{q}+{\bar{\eta }}_{2}^{q}}{1+{\bar{\eta }}_{1}^{q}.{\bar{\eta }}_{2}^{q}}},\sqrt[{q}]{\frac{{\bar{\xi }}_{1}^{q}+{\bar{\xi }}_{2}^{q}}{1+{\bar{\xi }}_{1}^{q}.{\bar{\xi }}_{2}^{q}}}\right\rangle \right]$$

=(0.1638,0.9002,0.5332,0.3553,0.9710,0.5104)

$$\omega_{Ḛ}A=\left[\left\langle \sqrt[{q}]{\frac{{\left(1+{\underline{\zeta }}^{q}\right)}^{\omega }-{\left(1-{\underline{\zeta }}^{q}\right)}^{\omega }}{{\left(1+{\underline{\zeta }}^{q}\right)}^{\omega }+{\left(1-{\underline{\zeta }}^{q}\right)}^{\omega }}},\sqrt[{q}]{\frac{2{\left({\underline{\eta }}^{q}\right)}^{\omega }}{{\left(2-{\underline{\eta }}^{q}\right)}^{\omega }+{\left({\underline{\eta }}^{q}\right)}^{\omega }}},\sqrt[{q}]{\frac{2{\left({\underline{\xi }}^{q}\right)}^{\omega }}{{\left(2-{\underline{\xi }}^{q}\right)}^{\omega }+{\left({\underline{\xi }}^{q}\right)}^{\omega }}},\sqrt[{q}]{\frac{{\left(1+{\bar{\zeta }}^{q}\right)}^{\omega }-{\left(1-{\bar{\zeta }}^{q}\right)}^{\omega }}{{\left(1+{\bar{\zeta }}^{q}\right)}^{\omega }+{\left(1-{\bar{\zeta }}^{q}\right)}^{\omega }}},\sqrt[{q}]{\frac{2{\left({\bar{\eta }}^{q}\right)}^{\omega }}{{\left(2-{\bar{\eta }}^{q}\right)}^{\omega }+{\left({\bar{\eta }}^{q}\right)}^{\omega }}},\sqrt[{q}]{\frac{2{\left({\bar{\xi }}^{q}\right)}^{\omega }}{{\left(2-{\bar{\xi }}^{q}\right)}^{\omega }+{\left({\bar{\xi }}^{q}\right)}^{\omega }}}\right\rangle \right]$$

=(0.3176,0.8022,0.8552,0.3974,0.4921,0.9043)

$${\;A}^{{Ḛ}^{\omega }}=\left[\left\langle \sqrt[{q}]{\frac{2{\left({\underline{\zeta }}^{q}\right)}^{\omega }}{{\left(2-{\underline{\zeta }}^{q}\right)}^{\omega }+{\left({\underline{\zeta }}^{q}\right)}^{\omega }}},\sqrt[{q}]{\frac{{\left(1+{\underline{\eta }}^{q}\right)}^{\omega }-{\left(1-{\underline{\eta }}^{q}\right)}^{\omega }}{{\left(1+{\underline{\eta }}^{q}\right)}^{\omega }+{\left(1-{\underline{\eta }}^{q}\right)}^{\omega }}},\sqrt[{q}]{\frac{{\left(1+{\underline{\xi }}^{q}\right)}^{\omega }-{\left(1-{\underline{\xi }}^{q}\right)}^{\omega }}{{\left(1+{\underline{\xi }}^{q}\right)}^{\omega }+{\left(1-{\underline{\xi }}^{q}\right)}^{\omega }}},\frac{\sqrt[{q}]{2}{\left(\bar{\zeta }\right)}^{\omega }}{\sqrt[{q}]{{\left(2-\bar{\zeta }\right)}^{\omega }+{\left(\bar{\zeta }\right)}^{\omega }}},\sqrt[{q}]{\frac{{\left(1+{\bar{\eta }}^{q}\right)}^{\omega }-{\left(1-{\bar{\eta }}^{q}\right)}^{\omega }}{{\left(1+{\bar{\eta }}^{q}\right)}^{\omega }+{\left(1-{\bar{\eta }}^{q}\right)}^{\omega }}},\sqrt[{q}]{\frac{{\left(1+{\bar{\xi }}^{q}\right)}^{\omega }-{\left(1-{\bar{\xi }}^{q}\right)}^{\omega }}{{\left(1+{\bar{\xi }}^{q}\right)}^{\omega }+{\left(1-{\bar{\xi }}^{q}\right)}^{\omega }}}\right\rangle \right]$$

=(0.6751,0.4781,0.5613,0.7432,0.1587,0.6506).

### 3.2 Q-SFREWA operators

Definition 14 Assuming $A_{i}=\left({\underline{\zeta }}_{i},{\underline{\eta }}_{i},{\underline{\xi }}_{i},{\bar{\zeta }}_{i},{\bar{\eta }}_{i},{\bar{\xi }}_{i}\right)\left(i=1,2,\ldots ,n\right)$ ne a collection of q-SFRNs, the q-spherical fuzzy rough Einstein averaging operator (q-SFREWA) operator is defined as a mapping $q-SFREWA:A^{n}⟶A$ characterized by

$$q-SFREWA\left(A_{1},A_{2},\ldots ,A_{n}\right)={{⊕}_{Ḛ}}_{i=1}^{n}\left(\omega_{i}A_{i}\right)$$

$$=\left[\left\langle \begin{array}{c} \sqrt[{q}]{\frac{\prod_{i=1}^{n}\;{\left(1+{\underline{\zeta }}_{i}^{q}\right)}^{\omega_{i}}-\prod_{i=1}^{n}\;{\left(1-{\underline{\zeta }}_{i}^{q}\right)}^{\omega_{i}}}{\prod_{i=1}^{n}\;{\left(1+{\underline{\zeta }}_{i}^{q}\right)}^{\omega_{i}}+{\prod_{i=1}^{n}\;\left(1-{\underline{\zeta }}_{i}^{q}\right)}^{\omega_{i}}}},\sqrt[{q}]{\frac{2{\prod_{i=1}^{n}\;\left({\underline{\eta }}_{i}^{q}\right)}^{\omega_{i}}}{{\prod_{i=1}^{n}\;\left(2-{\underline{\eta }}_{i}^{q}\right)}^{\omega_{i}}+\prod_{i=1}^{n}\;{\left({\underline{\eta }}_{i}^{q}\right)}^{\omega_{i}}}},\sqrt[{q}]{\frac{2{\prod_{i=1}^{n}\;\left({\underline{\xi }}_{i}^{q}\right)}^{\omega_{i}}}{{\prod_{i=1}^{n}\;\left(2-{\underline{\xi }}_{i}^{q}\right)}^{\omega_{i}}+\prod_{i=1}^{n}\;{\left({\underline{\xi }}_{i}^{q}\right)}^{\omega_{i}}},}\\ \sqrt[{q}]{\frac{\prod_{i=1}^{n}\;{\left(1+{\bar{\zeta }}_{i}^{q}\right)}^{\omega_{i}}-\prod_{i=1}^{n}\;{\left(1-{\bar{\zeta }}_{i}^{q}\right)}^{\omega_{i}}}{\prod_{i=1}^{n}\;{\left(1+{\bar{\zeta }}_{i}^{q}\right)}^{\omega_{i}}+{\prod_{i=1}^{n}\;\left(1-{\bar{\zeta }}_{i}^{q}\right)}^{\omega_{i}}}},\sqrt[{q}]{\frac{2{\prod_{i=1}^{n}\;\left(-{\bar{\eta }}_{i}^{q}\right)}^{\omega_{i}}}{{\prod_{i=1}^{n}\;\left(2-{\bar{\eta }}_{i}^{q}\right)}^{\omega_{i}}+\prod_{i=1}^{n}\;{\left(-{\bar{\eta }}_{i}^{q}\right)}^{\omega_{i}}}},\sqrt[{q}]{\frac{2{\prod_{i=1}^{n}\;\left({\bar{\xi }}_{i}^{q}\right)}^{\omega_{i}}}{{\prod_{i=1}^{n}\;\left(2-{\bar{\xi }}_{i}^{q}\right)}^{\omega_{i}}+\prod_{i=1}^{n}\;{\left({\bar{\xi }}_{i}^{q}\right)}^{\omega_{i}}}} \end{array}\right\rangle \right]$$

Hence ω=${\left(\omega_{1},\omega_{2},\ldots ,\omega_{n}\right)}^{T}$ signifies the weight vector of $A_{i}=\left({\underline{\zeta }}_{i},{\underline{\eta }}_{i},{\underline{\xi }}_{i},{\bar{\zeta }}_{i},{\bar{\eta }}_{i},{\bar{\xi }}_{i}\right)\left(i=1,2,\ldots ,n\right)$ adhering the conditions $\omega_{i}>0$ and the constrain $\sum_{i=1}^{n}\omega_{i}=1$.

Theorem 1 *Assuming*$A_{i}=\left({\underline{\zeta }}_{i},{\underline{\eta }}_{i},{\underline{\xi }}_{i},{\bar{\zeta }}_{i},{\bar{\eta }}_{i},{\bar{\xi }}_{i}\right)\left(i=1,2,\ldots ,n\right)$*be a collection of*$q-SFRNs\;\text{and}\;{\left(\omega_{1},\omega_{2},\ldots ,\omega_{n}\right)}^{T}$*signifies the weight vector adhering to the condition*$\omega_{i}>0$*and the constrain*$\sum_{i=1}^{n}\omega_{i}=1\text{.}$*Then the aggregated values obtained by the q-SFREWA operator is also a q-SFRN*.

**Proof:** This proof can be easily established using mathematical induction about the natural number n.

Step 1 When n=1 the value of $\omega_{1}$ become 1 and upon evaluating the left side of Definition (14), we get:

$$q-SFREWA\left(A_{1},A_{2},\ldots ,A_{n}\right)=A_{1}=\left({\underline{\zeta }}_{1},{\underline{\eta }}_{1},{\underline{\xi }}_{1},{\bar{\zeta }}_{1},{\bar{\eta }}_{1},{\bar{\xi }}_{1}\right)\text{.}$$

Also, for the right-hand side of Definition (14), we obtain:

$$\left[\left\langle \begin{array}{c} \sqrt[{q}]{\frac{{\left(1+{\underline{\zeta }}_{1}^{q}\right)}^{\omega_{1}}-{\left(1-{\underline{\zeta }}_{1}^{q}\right)}^{\omega_{1}}}{{\left(1+{\underline{\zeta }}_{1}^{q}\right)}^{\omega_{1}}+{\left(1-{\underline{\zeta }}_{1}^{q}\right)}^{\omega_{1}}}},\sqrt[{q}]{\frac{2{\left({\underline{\eta }}_{1}^{q}\right)}^{\omega_{1}}}{{\prod_{i=1}^{n}\;\left(2-{\underline{\eta }}_{1}^{q}\right)}^{\omega_{1}}+{\left({\underline{\eta }}_{1}^{q}\right)}^{\omega_{1}}}},\sqrt[{q}]{\frac{2{\prod_{i=1}^{n}\;\left({\underline{\xi }}_{1}^{q}\right)}^{\omega_{1}}}{{\left(2-{\underline{\xi }}_{1}^{q}\right)}^{\omega_{1}}+{\left({\underline{\xi }}_{1}^{q}\right)}^{\omega_{1}}},}\\ \sqrt[{q}]{\frac{{\left(1+{\bar{\zeta }}_{1}^{q}\right)}^{\omega_{1}}-{\left(1-{\bar{\zeta }}_{1}^{q}\right)}^{\omega_{1}}}{{\left(1+{\bar{\zeta }}_{1}^{q}\right)}^{\omega_{1}}+{\left(1-{\bar{\zeta }}_{1}^{q}\right)}^{\omega_{1}}}},\sqrt[{q}]{\frac{2{\left({\bar{\eta }}_{1}^{q}\right)}^{\omega_{1}}}{{\left(2-{\bar{\eta }}_{1}^{q}\right)}^{\omega_{1}}+{\left({\bar{\eta }}_{1}^{q}\right)}^{\omega_{1}}}},\sqrt[{q}]{\frac{2{\left({\bar{\xi }}_{1}^{q}\right)}^{\omega_{1}}}{{\left(2-{\bar{\xi }}_{1}^{q}\right)}^{\omega_{1}}+{\left({\bar{\xi }}_{1}^{q}\right)}^{\omega_{1}}}} \end{array}\right\rangle \right]=\left({\underline{\zeta }}_{1},{\underline{\eta }}_{1},{\underline{\xi }}_{1},{\bar{\zeta }}_{1},{\bar{\eta }}_{1},{\bar{\xi }}_{1}\right)$$

Thus, the condition n=1 is true for Definition (14).

Step 2 Assume that Definition (14), is valid for n=k, where k is any real number, given this supposition, Definition (14), can be represented as:

$$q-SFREWA\left(A_{1},A_{2},\ldots ,A_{k}\right)={{⊕}_{Ḛ}}_{i=1}^{k}\left(\omega_{i}A_{i}\right)$$

$$=\left[\left\langle \begin{array}{c} \sqrt[{q}]{\frac{\prod_{i=1}^{k}\;{\left(1+{\underline{\zeta }}_{i}^{q}\right)}^{\omega_{i}}-\prod_{i=1}^{n}\;{\left(1-{\underline{\zeta }}_{i}^{q}\right)}^{\omega_{i}}}{\prod_{i=1}^{k}\;{\left(1+{\underline{\zeta }}_{i}^{q}\right)}^{\omega_{i}}+{\prod_{i=1}^{n}\;\left(1-{\underline{\zeta }}_{i}^{q}\right)}^{\omega_{i}}}},\sqrt[{q}]{\frac{2{\prod_{i=1}^{k}\;\left({\underline{\eta }}_{i}^{q}\right)}^{\omega_{i}}}{{\prod_{i=1}^{k}\;\left(2-{\underline{\eta }}_{i}^{q}\right)}^{\omega_{i}}+\prod_{i=1}^{k}\;{\left({\underline{\eta }}_{i}^{q}\right)}^{\omega_{i}}}},\sqrt[{q}]{\frac{2{\prod_{i=1}^{k}\;\left({\underline{\xi }}_{i}^{q}\right)}^{\omega_{i}}}{{\prod_{i=1}^{k}\;\left(2-{\underline{\xi }}_{i}^{q}\right)}^{\omega_{i}}+\prod_{i=1}^{n}\;{\left({\underline{\xi }}_{i}^{q}\right)}^{\omega_{i}}},}\\ \sqrt[{q}]{\frac{\prod_{i=1}^{k}\;{\left(1+{\bar{\zeta }}_{i}^{q}\right)}^{\omega_{i}}-\prod_{i=1}^{k}\;{\left(1-{\bar{\zeta }}_{i}^{q}\right)}^{\omega_{i}}}{\prod_{i=1}^{k}\;{\left(1+{\bar{\zeta }}_{i}^{q}\right)}^{\omega_{i}}+{\prod_{i=1}^{k}\;\left(1-{\bar{\zeta }}_{i}^{q}\right)}^{\omega_{i}}}},\sqrt[{q}]{\frac{2{\prod_{i=1}^{k}\;\left({\bar{\eta }}_{i}^{q}\right)}^{\omega_{i}}}{{\prod_{i=1}^{k}\;\left(2-{\bar{\eta }}_{i}^{q}\right)}^{\omega_{i}}+\prod_{i=1}^{n}\;{\left({\bar{\eta }}_{i}^{q}\right)}^{\omega_{i}}}},\sqrt[{q}]{\frac{2{\prod_{i=1}^{k}\;\left({\bar{\xi }}_{i}^{q}\right)}^{\omega_{i}}}{{\prod_{i=1}^{k}\;\left(2-{\bar{\xi }}_{i}^{q}\right)}^{\omega_{i}}+\prod_{i=1}^{n}\;{\left({\bar{\xi }}_{i}^{q}\right)}^{\omega_{i}}}} \end{array}\right\rangle \right]$$

Step 3 Now for n=k+1, we are examining the following equations:

$$q-SFREWA\left(A_{1},A_{2},\ldots ,A_{k},A_{k+1}\right)={{⊕}_{Ḛ}}_{i=1}^{k}\left(\omega_{i}A_{i}\right){⊕}_{Ḛ}\omega_{k+1}A_{k+1}$$

$$=q-SFREWA\left(A_{1},A_{2},\ldots ,A_{n}\right)={{⊕}_{Ḛ}}_{i=1}^{n}\left(\omega_{i}A_{i}\right)$$

$$=\left[\left\langle \begin{array}{c} \sqrt[{q}]{\frac{\prod_{i=1}^{k}\;{\left(1+{\underline{\zeta }}_{i}^{q}\right)}^{\omega_{i}}-\prod_{i=1}^{n}\;{\left(1-{\underline{\zeta }}_{i}^{q}\right)}^{\omega_{i}}}{\prod_{i=1}^{k}\;{\left(1+{\underline{\zeta }}_{i}^{q}\right)}^{\omega_{i}}+{\prod_{i=1}^{n}\;\left(1-{\underline{\zeta }}_{i}^{q}\right)}^{\omega_{i}}}},\sqrt[{q}]{\frac{2{\prod_{i=1}^{k}\;\left({\underline{\eta }}_{i}^{q}\right)}^{\omega_{i}}}{{\prod_{i=1}^{k}\;\left(2-{\underline{\eta }}_{i}^{q}\right)}^{\omega_{i}}+\prod_{i=1}^{k}\;{\left({\underline{\eta }}_{i}^{q}\right)}^{\omega_{i}}}},\sqrt[{q}]{\frac{2{\prod_{i=1}^{k}\;\left({\underline{\xi }}_{i}^{q}\right)}^{\omega_{i}}}{{\prod_{i=1}^{k}\;\left(2-{\underline{\xi }}_{i}^{q}\right)}^{\omega_{i}}+\prod_{i=1}^{n}\;{\left({\underline{\xi }}_{i}^{q}\right)}^{\omega_{i}}},}\\ \sqrt[{q}]{\frac{\prod_{i=1}^{k}\;{\left(1+{\bar{\zeta }}_{i}^{q}\right)}^{\omega_{i}}-\prod_{i=1}^{k}\;{\left(1-{\bar{\zeta }}_{i}^{q}\right)}^{\omega_{i}}}{\prod_{i=1}^{k}\;{\left(1+{\bar{\zeta }}_{i}^{q}\right)}^{\omega_{i}}+{\prod_{i=1}^{k}\;\left(1-{\bar{\zeta }}_{i}^{q}\right)}^{\omega_{i}}}},\sqrt[{q}]{\frac{2{\prod_{i=1}^{k}\;\left({\bar{\eta }}_{i}^{q}\right)}^{\omega_{i}}}{{\prod_{i=1}^{k}\;\left(2-{\bar{\eta }}_{i}^{q}\right)}^{\omega_{i}}+\prod_{i=1}^{n}\;{\left({\bar{\eta }}_{i}^{q}\right)}^{\omega_{i}}}},\sqrt[{q}]{\frac{2{\prod_{i=1}^{k}\;\left({\bar{\xi }}_{i}^{q}\right)}^{\omega_{i}}}{{\prod_{i=1}^{k}\;\left(2-{\bar{\xi }}_{i}^{q}\right)}^{\omega_{i}}+\prod_{i=1}^{n}\;{\left({\bar{\xi }}_{i}^{q}\right)}^{\omega_{i}}}} \end{array}\right\rangle \right]$$

$${⊕}_{Ḛ}\left[\left\langle \begin{array}{c} \sqrt[{q}]{\frac{{\left(1+{\underline{\zeta }}_{k+1}^{q}\right)}^{\omega_{k+1}}-{\left(1-{\underline{\zeta }}_{i}^{q}\right)}^{\omega_{k+1}}}{{\left(1+{\underline{\zeta }}_{k+1}^{q}\right)}^{\omega_{k+1}}+{\left(1-{\underline{\zeta }}_{k+1}^{q}\right)}^{\omega_{k+1}}}},\sqrt[{q}]{\frac{2{\left({\underline{\eta }}_{k+1}^{q}\right)}^{\omega_{k+1}}}{{\left(2-{\underline{\eta }}_{k+1}^{q}\right)}^{\omega_{k+1}}+{\left({\underline{\eta }}_{k+1}^{q}\right)}^{\omega_{k+1}}}},\sqrt[{q}]{\frac{2{\left({\underline{\xi }}_{k+1}^{q}\right)}^{\omega_{k+1}}}{{\left(2-{\underline{\xi }}_{k+1}^{q}\right)}^{\omega_{k+1}}+{\left({\underline{\xi }}_{k+1}^{q}\right)}^{\omega_{k+1}}}},\\ \sqrt[{q}]{\frac{{\left(1+{\bar{\zeta }}_{k+1}^{q}\right)}^{\omega_{k+1}}-{\left(1-{\bar{\zeta }}_{k+1}^{q}\right)}^{\omega_{k+1}}}{{\left(1+{\bar{\zeta }}_{k+1}^{q}\right)}^{\omega_{k+1}}+{\left(1-{\bar{\zeta }}_{k+1}^{q}\right)}^{\omega_{k+1}}}},\sqrt[{q}]{\frac{2{\left({\bar{\eta }}_{k+1}^{q}\right)}^{\omega_{k+1}}}{{\left(2-{\bar{\eta }}_{k+1}^{q}\right)}^{\omega_{k+1}}+{\left({\bar{\eta }}_{k+1}^{q}\right)}^{\omega_{k+1}}}},\sqrt[{q}]{\frac{2{\prod_{i=1}^{k}\;\left({\bar{\xi }}_{k+1}^{q}\right)}^{\omega_{k+1}}}{{\left(2-{\bar{\xi }}_{k+1}^{q}\right)}^{\omega_{k+1}}+{\left({\bar{\xi }}_{k+1}^{q}\right)}^{\omega_{k+1}}}} \end{array}\right\rangle \;\right]$$

$$=\left[\left\langle \begin{array}{c} \sqrt[{q}]{\frac{\prod_{i=1}^{k+1}\;{\left(1+{\underline{\zeta }}_{i}^{q}\right)}^{\omega_{i}}-\prod_{i=1}^{k+1}\;{\left(1-{\underline{\zeta }}_{i}^{q}\right)}^{\omega_{i}}}{\prod_{i=1}^{k+1}\;{\left(1+{\underline{\zeta }}_{i}^{q}\right)}^{\omega_{i}}+{\prod_{i=1}^{k+1}\;\left(1-{\underline{\zeta }}_{i}^{q}\right)}^{\omega_{i}}}},\sqrt[{q}]{\frac{2{\prod_{i=1}^{k+1}\;\left({\underline{\eta }}_{i}^{q}\right)}^{\omega_{i}}}{{\prod_{i=1}^{k+1}\;\left(2-{\underline{\eta }}_{i}^{q}\right)}^{\omega_{i}}+\prod_{i=1}^{k}\;{\left({\underline{\eta }}_{i}^{q}\right)}^{\omega_{i}}}},\sqrt[{q}]{\frac{2{\prod_{i=1}^{k+1}\;\left({\underline{\xi }}_{i}^{q}\right)}^{\omega_{i}}}{{\prod_{i=1}^{k+1}\;\left(2-{\underline{\xi }}_{i}^{q}\right)}^{\omega_{i}}+\prod_{i=1}^{k+1}\;{\left({\underline{\xi }}_{i}^{q}\right)}^{\omega_{i}}}},\\ \sqrt[{q}]{\frac{\prod_{i=1}^{k+1}\;{\left(1+{\bar{\zeta }}_{i}^{q}\right)}^{\omega_{i}}-\prod_{i=1}^{k+1}\;{\left(1-{\bar{\zeta }}_{i}^{q}\right)}^{\omega_{i}}}{\prod_{i=1}^{k+1}\;{\left(1+{\bar{\zeta }}_{i}^{q}\right)}^{\omega_{i}}+{\prod_{i=1}^{k+1}\;\left(1-{\bar{\zeta }}_{i}^{q}\right)}^{\omega_{i}}}},\sqrt[{q}]{\frac{2{\prod_{i=1}^{k+1}\;\left({\bar{\eta }}_{i}^{q}\right)}^{\omega_{i}}}{\sqrt[{q}]{{\prod_{i=1}^{k+1}\;\left(2-{\bar{\eta }}_{i}^{q}\right)}^{\omega_{i}}+\prod_{i=1}^{k+1}\;{\left({\bar{\eta }}_{i}^{q}\right)}^{\omega_{i}}}}},\sqrt[{q}]{\frac{2{\prod_{i=1}^{k+1}\;\left({\bar{\xi }}_{i}^{q}\right)}^{\omega_{i}}}{{\prod_{i=1}^{k+1}\;\left(2-{\bar{\xi }}_{i}^{q}\right)}^{\omega_{i}}+\prod_{i=1}^{k+1}\;{\left({\bar{\xi }}_{i}^{q}\right)}^{\omega_{i}}}} \end{array}\right\rangle \right]\text{.}$$

Hence for n=k+1 Definition (14) holds. By combining observations from steps (1), (2), and (3), this result applies to all values of n inside the natural numbers.

Example 2 Consider four q-SFRNs $A_{1}=\left(0.4,0.1,0.3,0.2,0.4,0.3\right)\text{,}$ $A_{2}=\left(0.5,0.9,0.5,0.8,0.6,0.2\right)\text{,}$ $A_{3}=\left(0.5,0.9,0.4,0.2,0.6,0.3\right)$ and $A_{4}=\left(0.2,0.5,0.5,0.8,0.9,0.2\right)\text{.}$ If ω= ${\left(0.3,0.1,0.4,0.2\right)}^{T}$ and q=3 then the q-SFREWA operator, as defined in Definition (14), can be computed as:

$$\left[\left\langle \begin{array}{c} \sqrt[{q}]{\frac{\prod_{i=1}^{4}\;{\left(1+{\underline{\zeta }}_{i}^{q}\right)}^{\omega_{i}}-\prod_{i=1}^{4}\;{\left(1-{\underline{\zeta }}_{i}^{q}\right)}^{\omega_{i}}}{\prod_{i=1}^{4}\;{\left(1+{\underline{\zeta }}_{i}^{q}\right)}^{\omega_{i}}+{\prod_{i=1}^{4}\;\left(1-{\underline{\zeta }}_{i}^{q}\right)}^{\omega_{i}}}},\sqrt[{q}]{\frac{2{\prod_{i=1}^{4}\;\left({\underline{\eta }}_{i}^{q}\right)}^{\omega_{i}}}{{\prod_{i=1}^{4}\;\left(2-{\underline{\eta }}_{i}^{q}\right)}^{\omega_{i}}+\prod_{i=1}^{4}\;{\left({\underline{\eta }}_{i}^{q}\right)}^{\omega_{i}}}},\sqrt[{q}]{\frac{2{\prod_{i=1}^{4}\;\left({\underline{\xi }}_{i}^{q}\right)}^{\omega_{i}}}{{\prod_{i=1}^{4}\;\left(2-{\underline{\xi }}_{i}^{q}\right)}^{\omega_{i}}+\prod_{i=1}^{4}\;{\left({\underline{\xi }}_{i}^{q}\right)}^{\omega_{i}}},}\\ \sqrt[{q}]{\frac{\prod_{i=1}^{4}\;{\left(1+{\bar{\zeta }}_{i}^{q}\right)}^{\omega_{i}}-\prod_{i=1}^{4}\;{\left(1-{\bar{\zeta }}_{i}^{q}\right)}^{\omega_{i}}}{\prod_{i=1}^{4}\;{\left(1+{\bar{\zeta }}_{i}^{q}\right)}^{\omega_{i}}+{\prod_{i=1}^{4}\;\left(1-{\bar{\zeta }}_{i}^{q}\right)}^{\omega_{i}}}},\sqrt[{q}]{\frac{2{\prod_{i=1}^{4}\;\left({\bar{\eta }}_{i}^{q}\right)}^{\omega_{i}}}{{\prod_{i=1}^{4}\;\left(2-{\bar{\eta }}_{i}^{q}\right)}^{\omega_{i}}+\prod_{i=1}^{4}\;{\left({\bar{\eta }}_{i}^{q}\right)}^{\omega_{i}}}},\sqrt[{q}]{\frac{2{\prod_{i=1}^{4}\;\left({\bar{\xi }}_{i}^{q}\right)}^{\omega_{i}}}{{\prod_{i=1}^{4}\;\left(2-{\bar{\xi }}_{i}^{q}\right)}^{\omega_{i}}+\prod_{i=1}^{4}\;{\left({\bar{\xi }}_{i}^{q}\right)}^{\omega_{i}}}} \end{array}\right\rangle \right]=\left(\;0.4370,0.6819,0.6287,0.5577,0.7257,0.5390\;\right)$$

Theorem 2 *Assuming*$A_{i}=\left({\underline{\zeta }}_{i},{\underline{\eta }}_{i},{\underline{\xi }}_{i},{\bar{\zeta }}_{i},{\bar{\eta }}_{i},{\bar{\xi }}_{i}\right)\left(i=1,2,\ldots ,n\right)$*be a collection of*$q-SFRNs\;\text{and}\;{\left(\omega_{1},\omega_{2},\ldots ,\omega_{n}\right)}^{T}$*signifies the weight vector adhering to the condition*$\omega_{i}>0$*and the constrain*$\sum_{i=1}^{n}\omega_{i}=1\text{.}$*We can find the q-SFREWA operator by* :

$$q-SFREWA\left(A_{1},A_{2},\ldots ,A_{n}\right)={{⊕}_{Ḛ}}_{i=1}^{n}\left(\omega_{i}A_{i}\right)$$

$$=\left[\left\langle \begin{array}{c} \sqrt[{q}]{\frac{\prod_{i=1}^{n}\;{\left(1+{\underline{\zeta }}_{i}^{q}\right)}^{\omega_{i}}-\prod_{i=1}^{n}\;{\left(1-{\underline{\zeta }}_{i}^{q}\right)}^{\omega_{i}}}{\prod_{i=1}^{n}\;{\left(1+{\underline{\zeta }}_{i}^{q}\right)}^{\omega_{i}}+{\prod_{i=1}^{n}\;\left(1-{\underline{\zeta }}_{i}^{q}\right)}^{\omega_{i}}}},\sqrt[{q}]{\frac{2{\prod_{i=1}^{n}\;\left({\underline{\eta }}_{i}^{q}\right)}^{\omega_{i}}}{{\prod_{i=1}^{n}\;\left(2-{\underline{\eta }}_{i}^{q}\right)}^{\omega_{i}}+\prod_{i=1}^{n}\;{\left({\underline{\eta }}_{i}^{q}\right)}^{\omega_{i}}}},\sqrt[{q}]{\frac{2{\prod_{i=1}^{n}\;\left({\underline{\xi }}_{i}^{q}\right)}^{\omega_{i}}}{{\prod_{i=1}^{n}\;\left(2-{\underline{\xi }}_{i}^{q}\right)}^{\omega_{i}}+\prod_{i=1}^{n}\;{\left({\underline{\xi }}_{i}^{q}\right)}^{\omega_{i}}},}\\ \sqrt[{q}]{\frac{\prod_{i=1}^{n}\;{\left(1+{\bar{\zeta }}_{i}^{q}\right)}^{\omega_{i}}-\prod_{i=1}^{n}\;{\left(1-{\bar{\zeta }}_{i}^{q}\right)}^{\omega_{i}}}{\prod_{i=1}^{n}\;{\left(1+{\bar{\zeta }}_{i}^{q}\right)}^{\omega_{i}}+{\prod_{i=1}^{n}\;\left(1-{\bar{\zeta }}_{i}^{q}\right)}^{\omega_{i}}}},\sqrt[{q}]{\frac{{\prod_{i=1}^{n}\;\left({\bar{\eta }}_{i}^{q}\right)}^{\omega_{i}}}{{\prod_{i=1}^{n}\;\left(2-{\bar{\eta }}_{i}^{q}\right)}^{\omega_{i}}+\prod_{i=1}^{n}\;{\left({\bar{\eta }}_{i}^{q}\right)}^{\omega_{i}}}},\sqrt[{q}]{\frac{{\prod_{i=1}^{n}\;\left({\bar{\xi }}_{i}^{q}\right)}^{\omega_{i}}}{{\prod_{i=1}^{n}\;\left(2-{\bar{\xi }}_{i}^{q}\right)}^{\omega_{i}}+\prod_{i=1}^{n}\;{\left({\bar{\xi }}_{i}^{q}\right)}^{\omega_{i}}}} \end{array}\right\rangle \right]$$

**Proof**: This theorem is proven by using mathematical induction.

Step 1**:** Let Theorem (2) is true for n=2.

$$q-SFREWA\left(A_{1},A_{2}\right)=\omega_{1}A_{1}{⊕}_{Ḛ}\omega_{2}A_{2}$$

$$\left(\omega_{1}A_{1}\right)=\left[\left\langle \begin{array}{c} \sqrt[{q}]{\frac{{\left(1+{\underline{\zeta }}_{1}^{q}\right)}^{\omega_{1}}-{\left(1-{\underline{\zeta }}_{1}^{q}\right)}^{\omega_{1}}}{{\left(1+{\underline{\zeta }}_{1}^{q}\right)}^{\omega_{1}}+{\left(1-{\underline{\zeta }}_{1}^{q}\right)}^{\omega_{1}}}},\sqrt[{q}]{\frac{2{\left({\underline{\eta }}_{1}^{q}\right)}^{\omega_{1}}}{{\left(2-{\underline{\eta }}_{1}^{q}\right)}^{\omega_{1}}+{\left({\underline{\eta }}_{1}^{q}\right)}^{\omega_{1}}}},\sqrt[{q}]{\frac{2{\left({\underline{\xi }}_{1}^{q}\right)}^{\omega_{1}}}{{\left(2-{\underline{\xi }}_{1}^{q}\right)}^{\omega_{1}}+{\left({\underline{\xi }}_{1}^{q}\right)}^{\omega_{1}}},}\\ \sqrt[{q}]{\frac{{\left(1+{\bar{\zeta }}_{1}^{q}\right)}^{\omega_{1}}-{\left(1-{\bar{\zeta }}_{1}^{q}\right)}^{\omega_{1}}}{{\left(1+{\bar{\zeta }}_{1}^{q}\right)}^{\omega_{1}}+{\left(1-{\bar{\zeta }}_{1}^{q}\right)}^{\omega_{1}}}},\sqrt[{q}]{\frac{2{\left({\bar{\eta }}_{1}^{q}\right)}^{\omega_{1}}}{{\left(2-{\bar{\eta }}_{1}^{q}\right)}^{\omega_{1}}+{\left({\bar{\eta }}_{1}^{q}\right)}^{\omega_{1}}}},\sqrt[{q}]{\frac{2{\left({\bar{\eta }}_{1}^{q}\right)}^{\omega_{1}}}{{\left(2-{\bar{\xi }}_{1}^{q}\right)}^{\omega_{1}}+{\left({\bar{\eta }}_{1}^{q}\right)}^{\omega_{1}}}} \end{array}\right\rangle \right]$$

$$\left(\omega_{2}A_{2}\right)=\left[\left\langle \begin{array}{c} \sqrt[{q}]{\frac{{\left(1+{\underline{\zeta }}_{2}^{q}\right)}^{\omega_{2}}-{\left(1-{\underline{\zeta }}_{2}^{q}\right)}^{\omega_{2}}}{{\left(1+{\underline{\zeta }}_{2}^{q}\right)}^{\omega_{2}}+{\left(1-{\underline{\zeta }}_{2}^{q}\right)}^{\omega_{2}}}},\sqrt[{q}]{\frac{2{\left({\underline{\eta }}_{2}^{q}\right)}^{\omega_{2}}}{{\left(2-{\underline{\eta }}_{2}^{q}\right)}^{\omega_{2}}+{\left({\underline{\eta }}_{2}^{q}\right)}^{\omega_{2}}}},\sqrt[{q}]{\frac{2{\left({\underline{\xi }}_{2}^{q}\right)}^{\omega_{2}}}{{\left(2-{\underline{\xi }}_{2}^{q}\right)}^{\omega_{2}}+{\left({\underline{\xi }}_{2}^{q}\right)}^{\omega_{2}}}},\\ \sqrt[{q}]{\frac{{\left(1+{\bar{\zeta }}_{2}^{q}\right)}^{\omega_{2}}-{\left(1-{\bar{\zeta }}_{2}^{q}\right)}^{\omega_{2}}}{{\left(1+{\bar{\zeta }}_{2}^{q}\right)}^{\omega_{2}}+{\left(1-{\bar{\zeta }}_{2}^{q}\right)}^{\omega_{2}}}},\sqrt[{q}]{\frac{2{\left({\bar{\eta }}_{2}^{q}\right)}^{\omega_{2}}}{{\left(2-{\bar{\eta }}_{2}^{q}\right)}^{\omega_{2}}+{\left({\bar{\eta }}_{2}^{q}\right)}^{\omega_{2}}}},\sqrt[{q}]{\frac{2{\left({\bar{\xi }}_{2}^{q}\right)}^{\omega_{2}}}{{\left(2-{\bar{\xi }}_{2}^{q}\right)}^{\omega_{2}}+{\left({\bar{\xi }}_{2}^{q}\right)}^{\omega_{2}}}} \end{array}\right\rangle \right]$$

$$\omega_{1}A_{1}{⊕}_{Ḛ}\omega_{2}A_{2}=\left[\left\langle \begin{array}{c} \sqrt[{q}]{\frac{{\left(1+{\underline{\zeta }}_{1}^{q}\right)}^{\omega_{1}}-{\left(1-{\underline{\zeta }}_{1}^{q}\right)}^{\omega_{1}}}{{\left(1+{\underline{\zeta }}_{1}^{q}\right)}^{\omega_{1}}+{\left(1-{\underline{\zeta }}_{1}^{q}\right)}^{\omega_{1}}}},\sqrt[{q}]{\frac{2{\left({\underline{\eta }}_{1}^{q}\right)}^{\omega_{1}}}{{\left(2-{\underline{\eta }}_{1}^{q}\right)}^{\omega_{1}}+{\left({\underline{\eta }}_{1}^{q}\right)}^{\omega_{1}}}},\sqrt[{q}]{\frac{2{\left({\underline{\xi }}_{1}^{q}\right)}^{\omega_{1}}}{{\left(2-{\underline{\xi }}_{1}^{q}\right)}^{\omega_{1}}+{\left({\underline{\xi }}_{1}^{q}\right)}^{\omega_{1}}},}\\ \sqrt[{q}]{\frac{{\left(1+{\bar{\zeta }}_{1}^{q}\right)}^{\omega_{1}}-{\left(1-{\bar{\zeta }}_{1}^{q}\right)}^{\omega_{1}}}{{\left(1+{\bar{\zeta }}_{1}^{q}\right)}^{\omega_{1}}+{\left(1-{\bar{\zeta }}_{1}^{q}\right)}^{\omega_{1}}}},\sqrt[{q}]{\frac{2{\left({\bar{\eta }}_{1}^{q}\right)}^{\omega_{1}}}{{\left(2-{\bar{\eta }}_{1}^{q}\right)}^{\omega_{1}}+{\left({\bar{\eta }}_{1}^{q}\right)}^{\omega_{1}}}},\sqrt[{q}]{\frac{2{\left({\bar{\eta }}_{1}^{q}\right)}^{\omega_{1}}}{{\left(2-{\bar{\xi }}_{1}^{q}\right)}^{\omega_{1}}+{\left({\bar{\eta }}_{1}^{q}\right)}^{\omega_{1}}}} \end{array}\right\rangle \right]\;{⊕}_{Ḛ}\;\left[\left\langle \begin{array}{c} \sqrt[{q}]{\frac{{\left(1+{\underline{\zeta }}_{2}^{q}\right)}^{\omega_{2}}-{\left(1-{\underline{\zeta }}_{2}^{q}\right)}^{\omega_{2}}}{{\left(1+{\underline{\zeta }}_{2}^{q}\right)}^{\omega_{2}}+{\left(1-{\underline{\zeta }}_{2}^{q}\right)}^{\omega_{2}}}},\sqrt[{q}]{\frac{2{\left({\underline{\eta }}_{2}^{q}\right)}^{\omega_{2}}}{{\left(2-{\underline{\eta }}_{2}^{q}\right)}^{\omega_{2}}+{\left({\underline{\eta }}_{2}^{q}\right)}^{\omega_{2}}}},\sqrt[{q}]{\frac{2{\left({\underline{\xi }}_{2}^{q}\right)}^{\omega_{2}}}{{\left(2-{\underline{\xi }}_{2}^{q}\right)}^{\omega_{2}}+{\left({\underline{\xi }}_{2}^{q}\right)}^{\omega_{2}}}},\\ \sqrt[{q}]{\frac{{\left(1+{\bar{\zeta }}_{2}^{q}\right)}^{\omega_{2}}-{\left(1-{\bar{\zeta }}_{2}^{q}\right)}^{\omega_{2}}}{{\left(1+{\bar{\zeta }}_{2}^{q}\right)}^{\omega_{2}}+{\left(1-{\bar{\zeta }}_{2}^{q}\right)}^{\omega_{2}}}},\sqrt[{q}]{\frac{2{\left({\bar{\eta }}_{2}^{q}\right)}^{\omega_{2}}}{{\left(2-{\bar{\eta }}_{2}^{q}\right)}^{\omega_{2}}+{\left({\bar{\eta }}_{2}^{q}\right)}^{\omega_{2}}}},\sqrt[{q}]{\frac{2{\left({\bar{\xi }}_{2}^{q}\right)}^{\omega_{2}}}{{\left(2-{\bar{\xi }}_{2}^{q}\right)}^{\omega_{2}}+{\left({\bar{\xi }}_{2}^{q}\right)}^{\omega_{2}}}} \end{array}\right\rangle \right]$$

$$=\left[\left\langle \begin{array}{c} \sqrt[{q}]{\frac{\sqrt[{q}]{\frac{{\left(1+{\underline{\zeta }}_{1}^{q}\right)}^{\omega_{1}}-{\left(1-{\underline{\zeta }}_{1}^{q}\right)}^{\omega_{1}}}{{\left(1+{\underline{\zeta }}_{1}^{q}\right)}^{\omega_{1}}+{\left(1-{\underline{\zeta }}_{1}^{q}\right)}^{\omega_{1}}}}\;\sqrt[{q}]{\frac{{\left(1+{\underline{\zeta }}_{2}^{q}\right)}^{\omega_{2}}-{\left(1-{\underline{\zeta }}_{2}^{q}\right)}^{\omega_{2}}}{{\left(1+{\underline{\zeta }}_{2}^{q}\right)}^{\omega_{2}}+{\left(1-{\underline{\zeta }}_{2}^{q}\right)}^{\omega_{2}}}\;}}{1+\left(\sqrt[{q}]{\frac{{\left(1+{\underline{\zeta }}_{1}^{q}\right)}^{\omega_{1}}-{\left(1-{\underline{\zeta }}_{1}^{q}\right)}^{\omega_{1}}}{{\left(1+{\underline{\zeta }}_{1}^{q}\right)}^{\omega_{1}}+{\left(1-{\underline{\zeta }}_{1}^{q}\right)}^{\omega_{1}}}}\;\right)\left(\sqrt[{q}]{\frac{{\left(1+{\underline{\zeta }}_{2}^{q}\right)}^{\omega_{2}}-{\left(1-{\underline{\zeta }}_{2}^{q}\right)}^{\omega_{2}}}{{\left(1+{\underline{\zeta }}_{2}^{q}\right)}^{\omega_{2}}+{\left(1-{\underline{\zeta }}_{2}^{q}\right)}^{\omega_{2}}}\;}\right)}\;},\sqrt[{q}]{\frac{\left(\sqrt[{q}]{\frac{2{\left({\underline{\eta }}_{1}^{q}\right)}^{\omega_{1}}}{{\left(2-{\underline{\eta }}_{1}^{q}\right)}^{\omega_{1}}+{\left({\underline{\eta }}_{1}^{q}\right)}^{\omega_{1}}}}\right)\left(\sqrt[{q}]{\frac{2{\left({\underline{\eta }}_{2}^{q}\right)}^{\omega_{2}}}{{\left(2-{\underline{\eta }}_{2}^{q}\right)}^{\omega_{2}}+{\left({\underline{\eta }}_{2}^{q}\right)}^{\omega_{2}}}}\right)}{1+\left(1-\;\sqrt[{q}]{\frac{2{\left({\underline{\eta }}_{1}^{q}\right)}^{\omega_{1}}}{{\left(2-{\underline{\eta }}_{1}^{q}\right)}^{\omega_{1}}+{\left({\underline{\eta }}_{1}^{q}\right)}^{\omega_{1}}}}\right)\left(1-\;\sqrt[{q}]{\frac{2{\left({\underline{\eta }}_{2}^{q}\right)}^{\omega_{2}}}{{\left(2-{\underline{\eta }}_{2}^{q}\right)}^{\omega_{2}}+{\left({\underline{\eta }}_{2}^{q}\right)}^{\omega_{2}}}}\right)}},\sqrt[{q}]{\;\frac{\left(\sqrt[{q}]{\frac{2{\left({\underline{\xi }}_{1}^{q}\right)}^{\omega_{1}}}{{\left(2-{\underline{\xi }}_{1}^{q}\right)}^{\omega_{1}}+{\left({\underline{\xi }}_{1}^{q}\right)}^{\omega_{1}}}}\right)\left(\sqrt[{q}]{\frac{2{\left({\underline{\xi }}_{2}^{q}\right)}^{\omega_{2}}}{{\left(2-{\underline{\xi }}_{2}^{q}\right)}^{\omega_{2}}+{\left({\underline{\xi }}_{2}^{q}\right)}^{\omega_{2}}}}\right)}{1+\left(1-\;\sqrt[{q}]{\frac{2{\left({\underline{\xi }}_{1}^{q}\right)}^{\omega_{1}}}{{\left(2-{\underline{\xi }}_{1}^{q}\right)}^{\omega_{1}}+{\left({\underline{\xi }}_{1}^{q}\right)}^{\omega_{1}}}}\right)\left(1-\;\sqrt[{q}]{\frac{2{\left({\underline{\xi }}_{2}^{q}\right)}^{\omega_{2}}}{{\left(2-{\underline{\xi }}_{2}^{q}\right)}^{\omega_{2}}+{\left({\underline{\xi }}_{2}^{q}\right)}^{\omega_{2}}}}\right)}},\\ \sqrt[{q}]{\frac{\sqrt[{q}]{\frac{{\left(1+{\bar{\zeta }}_{1}^{q}\right)}^{\omega_{1}}-{\left(1-{\bar{\zeta }}_{1}^{q}\right)}^{\omega_{1}}}{{\left(1+{\bar{\zeta }}_{1}^{q}\right)}^{\omega_{1}}+{\left(1-{\bar{\zeta }}_{1}^{q}\right)}^{\omega_{1}}}}\;\sqrt[{q}]{\frac{{\left(1+{\bar{\zeta }}_{2}^{q}\right)}^{\omega_{2}}-{\left(1-{\bar{\zeta }}_{2}^{q}\right)}^{\omega_{2}}}{{\left(1+{\bar{\zeta }}_{2}^{q}\right)}^{\omega_{2}}+{\left(1-{\bar{\zeta }}_{2}^{q}\right)}^{\omega_{2}}}\;}}{1+\left(\sqrt[{q}]{\frac{{\left(1+{\bar{\zeta }}_{1}^{q}\right)}^{\omega_{1}}-{\left(1-{\bar{\zeta }}_{1}^{q}\right)}^{\omega_{1}}}{{\left(1+{\bar{\zeta }}_{1}^{q}\right)}^{\omega_{1}}+{\left(1-{\bar{\zeta }}_{1}^{q}\right)}^{\omega_{1}}}}\;\right)\left(\sqrt[{q}]{\frac{{\left(1+{\bar{\zeta }}_{2}^{q}\right)}^{\omega_{2}}-{\left(1-{\bar{\zeta }}_{2}^{q}\right)}^{\omega_{2}}}{{\left(1+{\bar{\zeta }}_{2}^{q}\right)}^{\omega_{2}}+{\left(1-{\bar{\zeta }}_{2}^{q}\right)}^{\omega_{2}}}\;}\right)}\;},\sqrt[{q}]{\frac{\left(\sqrt[{q}]{\frac{2{\left({\bar{\eta }}_{1}^{q}\right)}^{\omega_{1}}}{\left(2-{\bar{\eta }}_{1}^{q}\right)s^{\omega_{1}}+{\left({\bar{\eta }}_{1}^{q}\right)}^{\omega_{1}}}}\right)\left(\sqrt[{q}]{\frac{2{\left({\bar{\eta }}_{2}^{q}\right)}^{\omega_{2}}}{{\left(2-{\bar{\eta }}_{2}^{q}\right)}^{\omega_{2}}+{\left({\bar{\eta }}_{2}^{q}\right)}^{\omega_{2}}}}\right)}{1+\left(1-\;\sqrt[{q}]{\frac{2{\left({\bar{\eta }}_{1}^{q}\right)}^{\omega_{1}}}{{\left(2-{\bar{\eta }}_{1}^{q}\right)}^{\omega_{1}}+{\left({\bar{\eta }}_{1}^{q}\right)}^{\omega_{1}}}}\right)\left(1-\;\sqrt[{q}]{\frac{2{\left({\bar{\eta }}_{2}^{q}\right)}^{\omega_{2}}}{{\left(2-{\bar{\eta }}_{2}^{q}\right)}^{\omega_{2}}+{\left({\bar{\eta }}_{2}^{q}\right)}^{\omega_{2}}}}\right)}},\sqrt[{q}]{\frac{\left(\sqrt[{q}]{\frac{2{\left({\bar{\xi }}_{1}^{q}\right)}^{\omega_{1}}}{{\left(2-{\bar{\xi }}_{1}^{q}\right)}^{\omega_{1}}+{\left({\bar{\xi }}_{1}^{q}\right)}^{\omega_{1}}}}\right)\left(\sqrt[{q}]{\frac{2{\left({\bar{\xi }}_{2}^{q}\right)}^{\omega_{2}}}{{\left(2-{\bar{\xi }}_{2}^{q}\right)}^{\omega_{2}}+{\left({\bar{\xi }}_{2}^{q}\right)}^{\omega_{2}}}}\right)}{1+\left(1-\;\sqrt[{q}]{\frac{2{\left({\bar{\xi }}_{1}^{q}\right)}^{\omega_{1}}}{{\left(2-{\bar{\xi }}_{1}^{q}\right)}^{\omega_{1}}+{\left({\bar{\xi }}_{1}^{q}\right)}^{\omega_{1}}}}\right)\left(1-\;\sqrt[{q}]{\frac{2{\left({\bar{\xi }}_{2}^{q}\right)}^{\omega_{2}}}{{\left(2-{\bar{\xi }}_{2}^{q}\right)}^{\omega_{2}}+{\left({\bar{\xi }}_{2}^{q}\right)}^{\omega_{2}}}}\right)}} \end{array}\right\rangle \right]$$

$$=\left[\left\langle \begin{array}{c} \sqrt[{q}]{\frac{{\left(1+{\underline{\zeta }}_{1}^{q}\right)}^{\omega_{1}}.{\left(1+{\underline{\zeta }}_{2}^{q}\right)}^{\omega_{2}}-{\left(1-{\underline{\zeta }}_{1}^{q}\right)}^{\omega_{1}}.{\left(1-{\underline{\zeta }}_{2}^{q}\right)}^{\omega_{2}}}{{\left(1+{\underline{\zeta }}_{1}^{q}\right)}^{\omega_{1}}.{\left(1+{\underline{\zeta }}_{2}^{q}\right)}^{\omega_{2}}+{\left(1-{\underline{\zeta }}_{1}^{q}\right)}^{\omega_{1}}.{\left(1-{\underline{\zeta }}_{2}^{q}\right)}^{\omega_{2}}}},\sqrt[{q}]{\frac{2{\left({\underline{\eta }}_{1}^{q}\right)}^{\omega_{1}}.{\left({\underline{\eta }}_{2}^{q}\right)}^{\omega_{2}}}{{\left(2-{\underline{\eta }}_{1}^{q}\right)}^{\omega_{1}}.{\left(2-{\underline{\eta }}_{2}^{q}\right)}^{\omega_{2}}+{\left({\underline{\eta }}_{1}^{q}\right)}^{\omega_{1}}{.\left({\underline{\eta }}_{2}^{q}\right)}^{\omega_{2}}}},\sqrt[{q}]{\frac{2{\left({\underline{\xi }}_{1}^{q}\right)}^{\omega_{1}}.{\left({\underline{\xi }}_{2}^{q}\right)}^{\omega_{2}}}{{\left(2-{\underline{\xi }}_{1}^{q}\right)}^{\omega_{1}}.{\left(2-{\underline{\xi }}_{2}^{q}\right)}^{\omega_{2}}+{\left({\underline{\xi }}_{1}^{q}\right)}^{\omega_{1}}.{\left({\underline{\xi }}_{2}^{q}\right)}^{\omega_{2}}}},\\ \sqrt[{q}]{\frac{{\left(1+{\bar{\zeta }}_{1}^{q}\right)}^{\omega_{1}}.{\left(1+{\bar{\zeta }}_{2}^{q}\right)}^{\omega_{2}}-{\left(1-{\bar{\zeta }}_{1}^{q}\right)}^{\omega_{1}}.{\left(1-{\bar{\zeta }}_{2}^{q}\right)}^{\omega_{2}}}{{\left(1+{\bar{\zeta }}_{1}^{q}\right)}^{\omega_{1}}.{\left(1+{\bar{\zeta }}_{2}^{q}\right)}^{\omega_{2}}+{\left(1-{\bar{\zeta }}_{1}^{q}\right)}^{\omega_{1}}.{\left(1-{\bar{\zeta }}_{2}^{q}\right)}^{\omega_{2}}}},\sqrt[{q}]{\frac{2{\left({\bar{\eta }}_{1}^{q}\right)}^{\omega_{1}}.{\left({\bar{\eta }}_{2}^{q}\right)}^{\omega_{2}}}{{\left(2-{\bar{\eta }}_{1}^{q}\right)}^{\omega_{1}}.{\left(2-{\bar{\eta }}_{2}^{q}\right)}^{\omega_{2}}+{\left({\bar{\eta }}_{1}^{q}\right)}^{\omega_{1}}{.\left({\bar{\eta }}_{2}^{q}\right)}^{\omega_{2}}}},\sqrt[{q}]{\frac{2{\left({\bar{\xi }}_{1}^{q}\right)}^{\omega_{1}}.{\left({\bar{\xi }}_{2}^{q}\right)}^{\omega_{2}}}{{\left(2-{\bar{\xi }}_{1}^{q}\right)}^{\omega_{1}}.{\left(2-{\bar{\xi }}_{2}^{q}\right)}^{\omega_{2}}+{\left({\bar{\xi }}_{1}^{q}\right)}^{\omega_{1}}.{\left({\bar{\xi }}_{2}^{q}\right)}^{\omega_{2}}}} \end{array}\right\rangle \right]$$

$$=\left[\left\langle \begin{array}{c} \sqrt[{q}]{\frac{\prod_{i=1}^{2}\;{\left(1+{\underline{\zeta }}_{i}^{q}\right)}^{\omega_{i}}-\prod_{i=1}^{2}\;{\left(1-{\underline{\zeta }}_{i}^{q}\right)}^{\omega_{i}}}{\prod_{i=1}^{2}\;{\left(1+{\underline{\zeta }}_{i}^{q}\right)}^{\omega_{i}}+{\prod_{i=1}^{2}\;\left(1-{\underline{\zeta }}_{i}^{q}\right)}^{\omega_{i}}}},\sqrt[{q}]{\frac{2{\prod_{i=1}^{2}\;\left({\underline{\eta }}_{i}^{q}\right)}^{\omega_{i}}}{{\prod_{i=1}^{2}\;\left(2-{\underline{\eta }}_{i}^{q}\right)}^{\omega_{i}}+\prod_{i=1}^{2}\;{\left({\underline{\eta }}_{i}^{q}\right)}^{\omega_{i}}}},\sqrt[{q}]{\frac{2{\prod_{i=1}^{2}\;\left({\underline{\xi }}_{i}^{q}\right)}^{\omega_{i}}}{{\prod_{i=1}^{2}\;\left(2-{\underline{\xi }}_{i}^{q}\right)}^{\omega_{i}}+\prod_{i=1}^{2}\;{\left({\underline{\xi }}_{i}^{q}\right)}^{\omega_{i}}},}\\ \sqrt[{q}]{\frac{\prod_{i=1}^{2}\;{\left(1+{\bar{\zeta }}_{i}^{q}\right)}^{\omega_{i}}-\prod_{i=1}^{2}\;{\left(1-{\bar{\zeta }}_{i}^{q}\right)}^{\omega_{i}}}{\prod_{i=1}^{2}\;{\left(1+{\bar{\zeta }}_{i}^{q}\right)}^{\omega_{i}}+{\prod_{i=1}^{2}\;\left(1-{\bar{\zeta }}_{i}^{q}\right)}^{\omega_{i}}}},\sqrt[{q}]{\frac{2{\prod_{i=1}^{2}\;\left({\bar{\eta }}_{i}^{q}\right)}^{\omega_{i}}}{{\prod_{i=1}^{2}\;\left(2-{\bar{\eta }}_{i}^{q}\right)}^{\omega_{i}}+\prod_{i=1}^{2}\;{\left({\bar{\eta }}_{i}^{q}\right)}^{\omega_{i}}}},\sqrt[{q}]{\frac{2{\prod_{i=1}^{2}\;\left({\bar{\xi }}_{i}^{q}\right)}^{\omega_{i}}}{{\prod_{i=1}^{2}\;\left(2-{\bar{\xi }}_{i}^{q}\right)}^{\omega_{i}}+\prod_{i=1}^{2}\;{\left({\bar{\xi }}_{i}^{q}\right)}^{\omega_{i}}}} \end{array}\right\rangle \right]\text{.}$$

Step 2**:** Suppose that Theorem (2) is true for n=k.

$$q-SFREWA\left(A_{1},A_{2},\ldots ,A_{k}\right)={{⊕}_{Ḛ}}_{i=1}^{k}\left(\omega_{i}A_{i}\right)$$

$$=\left[\left\langle \begin{array}{c} \sqrt[{q}]{\frac{\prod_{i=1}^{k}\;{\left(1+{\underline{\zeta }}_{i}^{q}\right)}^{\omega_{i}}-\prod_{i=1}^{k}\;{\left(1-{\underline{\zeta }}_{i}^{q}\right)}^{\omega_{i}}}{\prod_{i=1}^{k}\;{\left(1+{\underline{\zeta }}_{i}^{q}\right)}^{\omega_{i}}+{\prod_{i=1}^{k}\;\left(1-{\underline{\zeta }}_{i}^{q}\right)}^{\omega_{i}}}},\sqrt[{q}]{\frac{2{\prod_{i=1}^{k}\;\left({\underline{\eta }}_{i}^{q}\right)}^{\omega_{i}}}{{\prod_{i=1}^{k}\;\left(2-{\underline{\eta }}_{i}^{q}\right)}^{\omega_{i}}+\prod_{i=1}^{k}\;{\left({\underline{\eta }}_{i}^{q}\right)}^{\omega_{i}}}},\sqrt[{q}]{\frac{2{\prod_{i=1}^{k}\;\left({\underline{\xi }}_{i}^{q}\right)}^{\omega_{i}}}{{\prod_{i=1}^{k}\;\left(2-{\underline{\xi }}_{i}^{q}\right)}^{\omega_{i}}+\prod_{i=1}^{k}\;{\left({\underline{\xi }}_{i}^{q}\right)}^{\omega_{i}}},}\\ \sqrt[{q}]{\frac{\prod_{i=1}^{k}\;{\left(1+{\bar{\zeta }}_{i}^{q}\right)}^{\omega_{i}}-\prod_{i=1}^{k}\;{\left(1-{\bar{\zeta }}_{i}^{q}\right)}^{\omega_{i}}}{\prod_{i=1}^{k}\;{\left(1+{\bar{\zeta }}_{i}^{q}\right)}^{\omega_{i}}+{\prod_{i=1}^{k}\;\left(1-{\bar{\zeta }}_{i}^{q}\right)}^{\omega_{i}}}},\sqrt[{q}]{\frac{2{\prod_{i=1}^{k}\;\left({\bar{\eta }}_{i}^{q}\right)}^{\omega_{i}}}{{\prod_{i=1}^{k}\;\left(2-{\bar{\eta }}_{i}^{q}\right)}^{\omega_{i}}+\prod_{i=1}^{k}\;{\left({\bar{\eta }}_{i}^{q}\right)}^{\omega_{i}}}},\sqrt[{q}]{\frac{2{\prod_{i=1}^{k}\;\left({\bar{\xi }}_{i}^{q}\right)}^{\omega_{i}}}{{\prod_{i=1}^{k}\;\left(2-{\bar{\xi }}_{i}^{q}\right)}^{\omega_{i}}+\prod_{i=1}^{k}\;{\left({\bar{\xi }}_{i}^{q}\right)}^{\omega_{i}}}} \end{array}\right\rangle \right]$$

Step 3**:** We will prove that Theorem (2) is true for n=k+1.

$$q-SFREWA\left(A_{1},A_{2},\ldots ,A_{k},A_{k+1}\right)={{⊕}_{Ḛ}}_{i=1}^{k}\left(\omega_{i}A_{i}\right){⊕}_{Ḛ}\left(\omega_{k+1}A_{k+1}\right)$$

$$=\left[\left\langle \begin{array}{c} \sqrt[{q}]{\frac{\prod_{i=1}^{k}\;{\left(1+{\underline{\zeta }}_{i}^{q}\right)}^{\omega_{i}}-\prod_{i=1}^{k}\;{\left(1-{\underline{\zeta }}_{i}^{q}\right)}^{\omega_{i}}}{\prod_{i=1}^{k}\;{\left(1+{\underline{\zeta }}_{i}^{q}\right)}^{\omega_{i}}+{\prod_{i=1}^{k}\;\left(1-{\underline{\zeta }}_{i}^{q}\right)}^{\omega_{i}}}},\sqrt[{q}]{\frac{2{\prod_{i=1}^{k}\;\left({\underline{\eta }}_{i}^{q}\right)}^{\omega_{i}}}{{\prod_{i=1}^{k}\;\left(2-{\underline{\eta }}_{i}^{q}\right)}^{\omega_{i}}+\prod_{i=1}^{k}\;{\left({\underline{\eta }}_{i}^{q}\right)}^{\omega_{i}}}},\sqrt[{q}]{\frac{2{\prod_{i=1}^{k}\;\left({\underline{\xi }}_{i}^{q}\right)}^{\omega_{i}}}{{\prod_{i=1}^{k}\;\left(2-{\underline{\xi }}_{i}^{q}\right)}^{\omega_{i}}+\prod_{i=1}^{k}\;{\left({\underline{\xi }}_{i}^{q}\right)}^{\omega_{i}}},}\\ \sqrt[{q}]{\frac{\prod_{i=1}^{k}\;{\left(1+{\bar{\zeta }}_{i}^{q}\right)}^{\omega_{i}}-\prod_{i=1}^{k}\;{\left(1-{\bar{\zeta }}_{i}^{q}\right)}^{\omega_{i}}}{\prod_{i=1}^{k}\;{\left(1+{\bar{\zeta }}_{i}^{q}\right)}^{\omega_{i}}+{\prod_{i=1}^{k}\;\left(1-{\bar{\zeta }}_{i}^{q}\right)}^{\omega_{i}}}},\sqrt[{q}]{\frac{2{\prod_{i=1}^{k}\;\left({\bar{\eta }}_{i}^{q}\right)}^{\omega_{i}}}{{\prod_{i=1}^{k}\;\left(2-{\bar{\eta }}_{i}^{q}\right)}^{\omega_{i}}+\prod_{i=1}^{k}\;{\left({\bar{\eta }}_{i}^{q}\right)}^{\omega_{i}}}},\sqrt[{q}]{\frac{2{\prod_{i=1}^{k}\;\left({\bar{\xi }}_{i}^{q}\right)}^{\omega_{i}}}{{\prod_{i=1}^{k}\;\left(2-{\bar{\xi }}_{i}^{q}\right)}^{\omega_{i}}+\prod_{i=1}^{k}\;{\left({\bar{\xi }}_{i}^{q}\right)}^{\omega_{i}}}} \end{array}\right\rangle \right]$$

$${⊕}_{Ḛ}\left[\left\langle \begin{array}{c} \sqrt[{q}]{\frac{{\left(1+{\underline{\zeta }}_{k+1}^{q}\right)}^{\omega_{k+1}}-{\left(1-{\underline{\zeta }}_{k+1}^{q}\right)}^{\omega_{k+1}}}{{\left(1+{\underline{\zeta }}_{k+1}^{q}\right)}^{\omega_{k+1}}+{\left(1-{\underline{\zeta }}_{k+1}^{q}\right)}^{\omega_{k+1}}}},\sqrt[{q}]{\frac{2{\left({\underline{\eta }}_{k+1}^{q}\right)}^{\omega_{k+1}}}{{\left(2-{\underline{\eta }}_{k+1}^{q}\right)}^{\omega_{k+1}}+{\left({\underline{\eta }}_{k+1}^{q}\right)}^{\omega_{k+1}}}},\sqrt[{q}]{\frac{2{\left({\underline{\xi }}_{k+1}^{q}\right)}^{\omega_{k+1}}}{{\left(2-{\underline{\xi }}_{k+1}^{q}\right)}^{\omega_{k+1}}+{\left({\underline{\xi }}_{k+1}^{q}\right)}^{\omega_{k+1}}},}\\ \sqrt[{q}]{\frac{{\left(1+{\bar{\zeta }}_{k+1}^{q}\right)}^{\omega_{k+1}}-{\left(1-{\bar{\zeta }}_{k+1}^{q}\right)}^{\omega_{k+1}}}{{\left(1+{\bar{\zeta }}_{k+1}^{q}\right)}^{\omega_{k+1}}+{\left(1-{\bar{\zeta }}_{k+1}^{q}\right)}^{\omega_{k+1}}}},\sqrt[{q}]{\frac{2{\left({\bar{\eta }}_{k+1}^{q}\right)}^{\omega_{k+1}}}{{\left(2-{\bar{\eta }}_{k+1}^{q}\right)}^{\omega_{k+1}}+{\left({\bar{\eta }}_{k+1}^{q}\right)}^{\omega_{k+1}}}},\sqrt[{q}]{\frac{2{\left({\bar{\xi }}_{k+1}^{q}\right)}^{\omega_{k+1}}}{{\left(2-{\bar{\xi }}_{k+1}^{q}\right)}^{\omega_{k+1}}+{\left({\bar{\xi }}_{k+1}^{q}\right)}^{\omega_{k+1}}}} \end{array}\right\rangle \right]$$

$$=\left[\left\langle \begin{array}{c} \sqrt[{q}]{\frac{\prod_{i=1}^{k+1}\;{\left(1+{\underline{\zeta }}_{i}^{q}\right)}^{\omega_{i}}-\prod_{i=1}^{k+1}\;{\left(1-{\underline{\zeta }}_{i}^{q}\right)}^{\omega_{i}}}{\prod_{i=1}^{k+1}\;{\left(1+{\underline{\zeta }}_{i}^{q}\right)}^{\omega_{i}}+{\prod_{i=1}^{k+1}\;\left(1-{\underline{\zeta }}_{i}^{q}\right)}^{\omega_{i}}}},\sqrt[{q}]{\frac{2{\prod_{i=1}^{k+1}\;\left({\underline{\eta }}_{i}^{q}\right)}^{\omega_{i}}}{{\prod_{i=1}^{k+1}\;\left(2-{\underline{\eta }}_{i}^{q}\right)}^{\omega_{i}}+\prod_{i=1}^{k+1}\;{\left({\underline{\eta }}_{i}^{q}\right)}^{\omega_{i}}}},\sqrt[{q}]{\frac{2{\prod_{i=1}^{k+1}\;\left({\underline{\xi }}_{i}^{q}\right)}^{\omega_{i}}}{{\prod_{i=1}^{k+1}\;\left(2-{\underline{\xi }}_{i}^{q}\right)}^{\omega_{i}}+\prod_{i=1}^{k+1}\;{\left({\underline{\xi }}_{i}^{q}\right)}^{\omega_{i}}},}\\ \sqrt[{q}]{\frac{\prod_{i=1}^{k+1}\;{\left(1+{\bar{\zeta }}_{i}^{q}\right)}^{\omega_{i}}-\prod_{i=1}^{k+1}\;{\left(1-{\bar{\zeta }}_{i}^{q}\right)}^{\omega_{i}}}{\prod_{i=1}^{k+1}\;{\left(1+{\bar{\zeta }}_{i}^{q}\right)}^{\omega_{i}}+{\prod_{i=1}^{k+1}\;\left(1-{\bar{\zeta }}_{i}^{q}\right)}^{\omega_{i}}}},\sqrt[{q}]{\frac{2{\prod_{i=1}^{k+1}\;\left({\bar{\eta }}_{i}^{q}\right)}^{\omega_{i}}}{{\prod_{i=1}^{k+1}\;\left(2-{\bar{\eta }}_{i}^{q}\right)}^{\omega_{i}}+\prod_{i=1}^{k+1}\;{\left({\bar{\eta }}_{i}^{q}\right)}^{\omega_{i}}}},\sqrt[{q}]{\frac{2{\prod_{i=1}^{k+1}\;\left({\bar{\xi }}_{i}^{q}\right)}^{\omega_{i}}}{{\prod_{i=1}^{k+1}\;\left(2-{\bar{\xi }}_{i}^{q}\right)}^{\omega_{i}}+\prod_{i=1}^{k+1}\;{\left({\bar{\xi }}_{i}^{q}\right)}^{\omega_{i}}}} \end{array}\right\rangle \right]$$

Thus, the result holds for n=k+1. This proves the required result.

Theorem 3 (Idempotency) *Assuming*$A_{i}=\left({\underline{\zeta }}_{i},{\underline{\eta }}_{i},{\underline{\xi }}_{i},{\bar{\zeta }}_{i},{\bar{\eta }}_{i},{\bar{\xi }}_{i}\right)\left(i=1,2,\ldots ,n\right)$*be a collection of*$q-SFRNs\;\text{and}\;{\left(\omega_{1},\omega_{2},\ldots ,\omega_{n}\right)}^{T}$*signifies the weight vector adhering to the condition*$\omega_{i}>0$*and the constrain*$\sum_{i=1}^{n}\omega_{i}=1\text{.}$$A_{i}\left(i=1,2,\ldots ,n\right)$*are the same*∀i,*then*

$$q-SFREWA\left(A_{1},A_{2},\ldots ,A_{n}\right)=A$$

Proof: From Theorem 2, we have

$$q-SFREWA\left(A_{1},A_{2},\ldots ,A_{n}\right)={{⊕}_{Ḛ}}_{i=1}^{n}\left(\omega_{i}A_{i}\right)=\left[\left\langle \begin{array}{c} \sqrt[{q}]{\frac{\prod_{i=1}^{n}\;{\left(1+{\underline{\zeta }}_{i}^{q}\right)}^{\omega_{i}}-\prod_{i=1}^{n}\;{\left(1-{\underline{\zeta }}_{i}^{q}\right)}^{\omega_{i}}}{\prod_{i=1}^{n}\;{\left(1+{\underline{\zeta }}_{i}^{q}\right)}^{\omega_{i}}+{\prod_{i=1}^{n}\;\left(1-{\underline{\zeta }}_{i}^{q}\right)}^{\omega_{i}}}},\sqrt[{q}]{\frac{2{\prod_{i=1}^{n}\;\left({\underline{\eta }}_{i}^{q}\right)}^{\omega_{i}}}{{\prod_{i=1}^{n}\;\left(2-{\underline{\eta }}_{i}^{q}\right)}^{\omega_{i}}+\prod_{i=1}^{n}\;{\left({\underline{\eta }}_{i}^{q}\right)}^{\omega_{i}}}},\sqrt[{q}]{\frac{2{\prod_{i=1}^{n}\;\left({\underline{\xi }}_{i}^{q}\right)}^{\omega_{i}}}{{\prod_{i=1}^{n}\;\left(2-{\underline{\xi }}_{i}^{q}\right)}^{\omega_{i}}+\prod_{i=1}^{n}\;{\left({\underline{\xi }}_{i}^{q}\right)}^{\omega_{i}}},}\\ \sqrt[{q}]{\frac{\prod_{i=1}^{n}\;{\left(1+{\bar{\zeta }}_{i}^{q}\right)}^{\omega_{i}}-\prod_{i=1}^{n}\;{\left(1-{\bar{\zeta }}_{i}^{q}\right)}^{\omega_{i}}}{\prod_{i=1}^{n}\;{\left(1+{\bar{\zeta }}_{i}^{q}\right)}^{\omega_{i}}+{\prod_{i=1}^{n}\;\left(1-{\bar{\zeta }}_{i}^{q}\right)}^{\omega_{i}}}},\sqrt[{q}]{\frac{{\prod_{i=1}^{n}\;\left({\bar{\eta }}_{i}^{q}\right)}^{\omega_{i}}}{{\prod_{i=1}^{n}\;\left(2-{\bar{\eta }}_{i}^{q}\right)}^{\omega_{i}}+\prod_{i=1}^{n}\;{\left({\bar{\eta }}_{i}^{q}\right)}^{\omega_{i}}}},\sqrt[{q}]{\frac{{\prod_{i=1}^{n}\;\left({\bar{\xi }}_{i}^{q}\right)}^{\omega_{i}}}{{\prod_{i=1}^{n}\;\left(2-{\bar{\xi }}_{i}^{q}\right)}^{\omega_{i}}+\prod_{i=1}^{n}\;{\left({\bar{\xi }}_{i}^{q}\right)}^{\omega_{i}}}} \end{array}\right\rangle \right]$$

$\text{Since}\;A_{i}\left(i=1,2,\ldots ,n\right)$ are the same ∀i, so

$$q-SFREWA\left(A_{1},A_{2},\ldots ,A_{n}\right)=\left[\left\langle \begin{array}{c} \sqrt[{q}]{\frac{\prod_{i=1}^{n}\;{\left(1+{\underline{\zeta }}_{i}^{q}\right)}^{\omega_{i}}-\prod_{i=1}^{n}\;{\left(1-{\underline{\zeta }}_{i}^{q}\right)}^{\omega_{i}}}{\prod_{i=1}^{n}\;{\left(1+{\underline{\zeta }}_{i}^{q}\right)}^{\omega_{i}}+{\prod_{i=1}^{n}\;\left(1-{\underline{\zeta }}_{i}^{q}\right)}^{\omega_{i}}}},\sqrt[{q}]{\frac{2{\prod_{i=1}^{n}\;\left({\underline{\eta }}_{i}^{q}\right)}^{\omega_{i}}}{{\prod_{i=1}^{n}\;\left(2-{\underline{\eta }}_{i}^{q}\right)}^{\omega_{i}}+\prod_{i=1}^{n}\;{\left({\underline{\eta }}_{i}^{q}\right)}^{\omega_{i}}}},\sqrt[{q}]{\frac{2{\prod_{i=1}^{n}\;\left({\underline{\xi }}_{i}^{q}\right)}^{\omega_{i}}}{{\prod_{i=1}^{n}\;\left(2-{\underline{\xi }}_{i}^{q}\right)}^{\omega_{i}}+\prod_{i=1}^{n}\;{\left({\underline{\xi }}_{i}^{q}\right)}^{\omega_{i}}},}\\ \sqrt[{q}]{\frac{\prod_{i=1}^{n}\;{\left(1+{\bar{\zeta }}_{i}^{q}\right)}^{\omega_{i}}-\prod_{i=1}^{n}\;{\left(1-{\bar{\zeta }}_{i}^{q}\right)}^{\omega_{i}}}{\prod_{i=1}^{n}\;{\left(1+{\bar{\zeta }}_{i}^{q}\right)}^{\omega_{i}}+{\prod_{i=1}^{n}\;\left(1-{\bar{\zeta }}_{i}^{q}\right)}^{\omega_{i}}}},\sqrt[{q}]{\frac{{\prod_{i=1}^{n}\;\left({\bar{\eta }}_{i}^{q}\right)}^{\omega_{i}}}{{\prod_{i=1}^{n}\;\left(2-{\bar{\eta }}_{i}^{q}\right)}^{\omega_{i}}+\prod_{i=1}^{n}\;{\left({\bar{\eta }}_{i}^{q}\right)}^{\omega_{i}}}},\sqrt[{q}]{\frac{{\prod_{i=1}^{n}\;\left({\bar{\xi }}_{i}^{q}\right)}^{\omega_{i}}}{{\prod_{i=1}^{n}\;\left(2-{\bar{\xi }}_{i}^{q}\right)}^{\omega_{i}}+\prod_{i=1}^{n}\;{\left({\bar{\xi }}_{i}^{q}\right)}^{\omega_{i}}}} \end{array}\right\rangle \right]$$

$$=\left[\left\langle \begin{array}{c} \sqrt[{q}]{\frac{\prod_{i=1}^{n}\;{\left(1+{\underline{\zeta }}^{q}\right)}^{\omega_{i}}-\prod_{i=1}^{n}\;{\left(1-{\underline{\zeta }}^{q}\right)}^{\omega_{i}}}{\prod_{i=1}^{n}\;{\left(1+{\underline{\zeta }}^{q}\right)}^{\omega_{i}}+{\prod_{i=1}^{n}\;\left(1-{\underline{\zeta }}^{q}\right)}^{\omega_{i}}}},\sqrt[{q}]{\frac{2{\prod_{i=1}^{n}\;\left({\underline{\eta }}^{q}\right)}^{\omega_{i}}}{{\prod_{i=1}^{n}\;\left(2-{\underline{\eta }}^{q}\right)}^{\omega_{i}}+\prod_{i=1}^{n}\;{\left({\underline{\eta }}^{q}\right)}^{\omega_{i}}}},\sqrt[{q}]{\frac{2{\prod_{i=1}^{n}\;\left({\underline{\xi }}^{q}\right)}^{\omega_{i}}}{{\prod_{i=1}^{n}\;\left(2-{\underline{\xi }}^{q}\right)}^{\omega_{i}}+\prod_{i=1}^{n}\;{\left({\underline{\xi }}^{q}\right)}^{\omega_{i}}},}\\ \sqrt[{q}]{\frac{\prod_{i=1}^{n}\;{\left(1+{\bar{\zeta }}^{q}\right)}^{\omega_{i}}-\prod_{i=1}^{n}\;{\left(1-{\bar{\zeta }}^{q}\right)}^{\omega_{i}}}{\prod_{i=1}^{n}\;{\left(1+{\bar{\zeta }}^{q}\right)}^{\omega_{i}}+{\prod_{i=1}^{n}\;\left(1-{\bar{\zeta }}^{q}\right)}^{\omega_{i}}}},\sqrt[{q}]{\frac{{\prod_{i=1}^{n}\;\left({\bar{\eta }}^{q}\right)}^{\omega_{i}}}{{\prod_{i=1}^{n}\;\left(2-{\bar{\eta }}^{q}\right)}^{\omega_{i}}+\prod_{i=1}^{n}\;{\left({\bar{\eta }}^{q}\right)}^{\omega_{i}}}},\sqrt[{q}]{\frac{{\prod_{i=1}^{n}\;\left({\bar{\xi }}^{q}\right)}^{\omega_{i}}}{{\prod_{i=1}^{n}\;\left(2-{\bar{\xi }}^{q}\right)}^{\omega_{i}}+\prod_{i=1}^{n}\;{\left({\bar{\xi }}^{q}\right)}^{\omega_{i}}}} \end{array}\right\rangle \right]=A$$

Theorem 4 (Boundness) *Assuming*$A_{i}=\left({\underline{\zeta }}_{i},{\underline{\eta }}_{i},{\underline{\xi }}_{i},{\bar{\zeta }}_{i},{\bar{\eta }}_{i},{\bar{\xi }}_{i}\right)\left(i=1,2,\ldots ,n\right)$*be a collection of*$q-SFRNs\;\text{and}\;{\left(\omega_{1},\omega_{2},\ldots ,\omega_{n}\right)}^{T}$*signifies the weight vector adhering to the condition*$\omega_{i}>0$*and the constrain*$\sum_{i=1}^{n}\omega_{i}=1$.

$$\text{Let}\;A^{-}=\left(\min\;{\underline{\zeta }}_{i},\max\;{\underline{\eta }}_{i},\max\;{\underline{\xi }}_{i},\min\;{\bar{\zeta }}_{i},\max\;{\bar{\eta }}_{i},\max\;{\bar{\xi }}_{i}\right)\;\text{and}\;A^{+}=\left(\max\;{\underline{\zeta }}_{i},\min\;{\underline{\eta }}_{i},\min\;{\underline{\xi }}_{i},\max\;{\bar{\zeta }}_{i},\min\;{\bar{\eta }}_{i},\min\;{\bar{\xi }}_{i}\right)$$

$$\text{Then}\;A^{-}\leq q-SFREWA\left(A_{1},A_{2},\ldots ,A_{n}\right)\leq A^{+}$$

Proof: From Theorem 2, we have

$$q-SFREWA\left(A_{1},A_{2},\ldots ,A_{n}\right)={{⊕}_{Ḛ}}_{i=1}^{n}\left(\omega_{i}A_{i}\right)=\left[\left\langle \begin{array}{c} \sqrt[{q}]{\frac{\prod_{i=1}^{n}\;{\left(1+{\underline{\zeta }}_{i}^{q}\right)}^{\omega_{i}}-\prod_{i=1}^{n}\;{\left(1-{\underline{\zeta }}_{i}^{q}\right)}^{\omega_{i}}}{\prod_{i=1}^{n}\;{\left(1+{\underline{\zeta }}_{i}^{q}\right)}^{\omega_{i}}+{\prod_{i=1}^{n}\;\left(1-{\underline{\zeta }}_{i}^{q}\right)}^{\omega_{i}}}},\sqrt[{q}]{\frac{2{\prod_{i=1}^{n}\;\left({\underline{\eta }}_{i}^{q}\right)}^{\omega_{i}}}{{\prod_{i=1}^{n}\;\left(2-{\underline{\eta }}_{i}^{q}\right)}^{\omega_{i}}+\prod_{i=1}^{n}\;{\left({\underline{\eta }}_{i}^{q}\right)}^{\omega_{i}}}},\sqrt[{q}]{\frac{2{\prod_{i=1}^{n}\;\left({\underline{\xi }}_{i}^{q}\right)}^{\omega_{i}}}{{\prod_{i=1}^{n}\;\left(2-{\underline{\xi }}_{i}^{q}\right)}^{\omega_{i}}+\prod_{i=1}^{n}\;{\left({\underline{\xi }}_{i}^{q}\right)}^{\omega_{i}}},}\\ \sqrt[{q}]{\frac{\prod_{i=1}^{n}\;{\left(1+{\bar{\zeta }}_{i}^{q}\right)}^{\omega_{i}}-\prod_{i=1}^{n}\;{\left(1-{\bar{\zeta }}_{i}^{q}\right)}^{\omega_{i}}}{\prod_{i=1}^{n}\;{\left(1+{\bar{\zeta }}_{i}^{q}\right)}^{\omega_{i}}+{\prod_{i=1}^{n}\;\left(1-{\bar{\zeta }}_{i}^{q}\right)}^{\omega_{i}}}},\sqrt[{q}]{\frac{{\prod_{i=1}^{n}\;\left({\bar{\eta }}_{i}^{q}\right)}^{\omega_{i}}}{{\prod_{i=1}^{n}\;\left(2-{\bar{\eta }}_{i}^{q}\right)}^{\omega_{i}}+\prod_{i=1}^{n}\;{\left({\bar{\eta }}_{i}^{q}\right)}^{\omega_{i}}}},\sqrt[{q}]{\frac{{\prod_{i=1}^{n}\;\left({\bar{\xi }}_{i}^{q}\right)}^{\omega_{i}}}{{\prod_{i=1}^{n}\;\left(2-{\bar{\xi }}_{i}^{q}\right)}^{\omega_{i}}+\prod_{i=1}^{n}\;{\left({\bar{\xi }}_{i}^{q}\right)}^{\omega_{i}}}} \end{array}\right\rangle \right]$$

#### 3.2.1 For lower and upper memberships

We have

$$\min_{i}{\underline{\zeta }}_{i}\leq {{\underline{\zeta }}_{i}}^{q}\leq \max_{i}{{\underline{\zeta }}_{i}}^{q}$$

$${\left(1+\min_{i}{{\underline{\zeta }}_{i}}^{q}\right)}^{\omega_{i}}\leq {\prod_{i=1}^{n}\;\left(1+{{\underline{\zeta }}_{i}}^{q}\right)}^{\omega_{i}}\leq {\left(1+\max_{i}{{\underline{\zeta }}_{i}}^{q}\right)}^{\omega_{i}}$$

$$⟹\sqrt[{q}]{\frac{\prod_{i=1}^{n}\;{\left(1+\min_{i}{{\underline{\zeta }}_{i}}^{q}\right)}^{\omega_{i}}-\prod_{i=1}^{n}\;{\left(1-\min_{i}{{\underline{\zeta }}_{i}}^{q}\right)}^{\omega_{i}}}{\prod_{i=1}^{n}\;{\left(1+\min_{i}{{\underline{\zeta }}_{i}}^{q}\right)}^{\omega_{i}}+{\prod_{i=1}^{n}\;\left(1-\min_{i}{{\underline{\zeta }}_{i}}^{q}\right)}^{\omega_{i}}}}\leq \sqrt[{q}]{\frac{\prod_{i=1}^{n}\;{\left(1+{{\underline{\zeta }}_{i}}^{q}\right)}^{\omega_{i}}-\prod_{i=1}^{n}\;{\left(1-{{\underline{\zeta }}_{i}}^{q}\right)}^{\omega_{i}}}{\prod_{i=1}^{n}\;{\left(1+{{\underline{\zeta }}_{i}}^{q}\right)}^{\omega_{i}}+{\prod_{i=1}^{n}\;\left(1-{{\underline{\zeta }}_{i}}^{q}\right)}^{\omega_{i}}}}\leq \sqrt[{q}]{\frac{\prod_{i=1}^{n}\;{\left(1+\max_{i}{{\underline{\zeta }}_{i}}^{q}\right)}^{\omega_{i}}-\prod_{i=1}^{n}\;{\left(1-\max_{i}{{\underline{\zeta }}_{i}}^{q}\right)}^{\omega_{i}}}{\prod_{i=1}^{n}\;{\left(1+\max_{i}{{\underline{\zeta }}_{i}}^{q}\right)}^{\omega_{i}}+{\prod_{i=1}^{n}\;\left(1-\max_{i}{{\underline{\zeta }}_{i}}^{q}\right)}^{\omega_{i}}}}$$

And since

$$\min_{i}{\bar{\zeta }}_{i}\leq {\bar{\zeta }}_{i}\leq \max_{i}{\bar{\zeta }}_{i}$$

$$\min_{i}{{\bar{\zeta }}_{i}}^{q}\leq {{\bar{\zeta }}_{i}}^{q}\leq \max_{i}{{\bar{\zeta }}_{i}}^{q}$$

$${\left(1+\min_{i}{{\bar{\zeta }}_{i}}^{q}\right)}^{\omega_{i}}\leq {\prod_{i=1}^{n}\;\left(1+{{\bar{\zeta }}_{i}}^{q}\right)}^{\omega_{i}}\leq {\left(1+\max_{i}{{\bar{\zeta }}_{i}}^{q}\right)}^{\omega_{i}}$$

$$⟹\sqrt[{q}]{\frac{\prod_{i=1}^{n}\;{\left(1+\min_{i}{{\bar{\zeta }}_{i}}^{q}\right)}^{\omega_{i}}-\prod_{i=1}^{n}\;{\left(1-\min_{i}{{\bar{\zeta }}_{i}}^{q}\right)}^{\omega_{i}}}{\prod_{i=1}^{n}\;{\left(1+\min_{i}{{\bar{\zeta }}_{i}}^{q}\right)}^{\omega_{i}}+{\prod_{i=1}^{n}\;\left(1-\min_{i}{{\bar{\zeta }}_{i}}^{q}\right)}^{\omega_{i}}}}\leq \sqrt[{q}]{\frac{\prod_{i=1}^{n}\;{\left(1+{{\bar{\zeta }}_{i}}^{q}\right)}^{\omega_{i}}-\prod_{i=1}^{n}\;{\left(1-{{\bar{\zeta }}_{i}}^{q}\right)}^{\omega_{i}}}{\prod_{i=1}^{n}\;{\left(1+{{\bar{\zeta }}_{i}}^{q}\right)}^{\omega_{i}}+{\prod_{i=1}^{n}\;\left(1-{{\bar{\zeta }}_{i}}^{q}\right)}^{\omega_{i}}}}\leq \sqrt[{q}]{\frac{\prod_{i=1}^{n}\;{\left(1+\max_{i}{{\bar{\zeta }}_{i}}^{q}\right)}^{\omega_{i}}-\prod_{i=1}^{n}\;{\left(1-\max_{i}{{\bar{\zeta }}_{i}}^{q}\right)}^{\omega_{i}}}{\prod_{i=1}^{n}\;{\left(1+\max_{i}{{\bar{\zeta }}_{i}}^{q}\right)}^{\omega_{i}}+{\prod_{i=1}^{n}\;\left(1-\max_{i}{{\bar{\zeta }}_{i}}^{q}\right)}^{\omega_{i}}}}$$

#### 3.2.2 For lower and upper neutral memberships

We have

$$\max_{i}{\underline{\eta }}_{i}\geq {\underline{\eta }}_{i}\geq \min_{i}{\underline{\eta }}_{i}$$

$$\max_{i}{{\underline{\eta }}_{i}}^{q}\geq {{\underline{\eta }}_{i}}^{q}\geq \min_{i}{{\underline{\eta }}_{i}}^{q}$$

$$\underset{i}{2\;\max}{\left({{\underline{\eta }}_{i}}^{q}\right)}^{\omega_{i}}\geq 2\prod_{j=1}^{n}\;{\left({{\underline{\eta }}_{i}}^{q}\right)}^{\omega_{i}}\geq \underset{i}{2\;\min}{\left({{\underline{\eta }}_{i}}^{q}\right)}^{\omega_{i}}$$

$$⟹\sqrt[{q}]{\frac{2\max_{i}{\left({\underline{\eta }}^{q}\right)}^{\omega_{i}}}{{\left(2-\max_{i}{\underline{\eta }}^{q}\right)}^{\omega_{i}}+\max_{i}{\left({\underline{\eta }}^{q}\right)}^{\omega_{i}}}}\geq \sqrt[{q}]{\frac{\underset{i}{2\prod_{i=1}^{n}\;}{\left({\underline{\eta }}^{q}\right)}^{\omega_{i}}}{{\left(2-{\underline{\eta }}^{q}\right)}^{\omega_{i}}+\prod_{i=1}^{n}\;{\left({\underline{\eta }}^{q}\right)}^{\omega_{i}}}\;}\geq \sqrt[{q}]{\frac{\underset{i}{2\;\min}{\left({\underline{\eta }}^{q}\right)}^{\omega_{i}}}{{\left(2-\min_{i}{\underline{\eta }}^{q}\right)}^{\omega_{i}}+\min_{i}{\left({\underline{\eta }}^{q}\right)}^{\omega_{i}}}}$$

Also, we have

$$\max_{i}{\bar{\eta }}_{i}\geq {\bar{\eta }}_{i}\geq \min_{i}{\bar{\eta }}_{i}$$

$$\max_{i}{{\bar{\eta }}_{i}}^{q}\geq {{\bar{\eta }}_{i}}^{q}\geq \min_{i}{{\bar{\eta }}_{i}}^{q}$$

$$\underset{i}{2\;\max}{\left({{\bar{\eta }}_{i}}^{q}\right)}^{\omega_{i}}\geq 2\prod_{j=1}^{n}\;{\left({{\bar{\eta }}_{i}}^{q}\right)}^{\omega_{i}}\geq \underset{i}{2\;\min}{\left({{\bar{\eta }}_{i}}^{q}\right)}^{\omega_{i}}$$

$$⟹\sqrt[{q}]{\frac{2\max_{i}{\left({{\bar{\eta }}_{i}}^{q}\right)}^{\omega_{i}}}{{\left(2-\max_{i}{{\bar{\eta }}_{i}}^{q}\right)}^{\omega_{i}}+\max_{i}{\left({{\bar{\eta }}_{i}}^{q}\right)}^{\omega_{i}}}}\geq \sqrt[{q}]{\frac{\underset{i}{2\prod_{i=1}^{n}\;}{\left({{\bar{\eta }}_{i}}^{q}\right)}^{\omega_{i}}}{{\left(2-{{\bar{\eta }}_{i}}^{q}\right)}^{\omega_{i}}+\prod_{i=1}^{n}\;{\left({{\bar{\eta }}_{i}}^{q}\right)}^{\omega_{i}}}\;}\geq \sqrt[{q}]{\frac{\underset{i}{2\;\min}{\left({{\bar{\eta }}_{i}}^{q}\right)}^{\omega_{i}}}{{\left(2-\min_{i}{{\bar{\eta }}_{i}}^{q}\right)}^{\omega_{i}}+\min_{i}{\left({{\bar{\eta }}_{i}}^{q}\right)}^{\omega_{i}}}}$$

#### 3.2.3 For lower and upper non-memberships

We have

$$\max_{i}{\underline{\xi }}_{i}\geq {\underline{\xi }}_{i}\geq \min_{i}{\underline{\xi }}_{i}$$

$$\max_{i}{{\underline{\xi }}_{i}}^{q}\geq {{\underline{\xi }}_{i}}^{q}\geq \min_{i}{{\underline{\xi }}_{i}}^{q}$$

$$\underset{i}{2\;\max}{\left({{\underline{\xi }}_{i}}^{q}\right)}^{\omega_{i}}\geq 2\prod_{j=1}^{n}\;{\left({{\underline{\xi }}_{i}}^{q}\right)}^{\omega_{i}}\geq \underset{i}{2\;\min}{\left({{\underline{\xi }}_{i}}^{q}\right)}^{\omega_{i}}$$

$$⟹\sqrt[{q}]{\frac{2\max_{i}{\left({{\underline{\xi }}_{i}}^{q}\right)}^{\omega_{i}}}{{\left(2-\max_{i}{{\underline{\xi }}_{i}}^{q}\right)}^{\omega_{i}}+\max_{i}{\left({{\underline{\xi }}_{i}}^{q}\right)}^{\omega_{i}}}}\geq \sqrt[{q}]{\frac{\underset{i}{2\prod_{i=1}^{n}\;}{\left({{\underline{\xi }}_{i}}^{q}\right)}^{\omega_{i}}}{{\left(2-{{\underline{\xi }}_{i}}^{q}\right)}^{\omega_{i}}+\prod_{i=1}^{n}\;{\left({{\underline{\xi }}_{i}}^{q}\right)}^{\omega_{i}}}\;}\geq \sqrt[{q}]{\frac{\underset{i}{2\;\min}{\left({{\underline{\xi }}_{i}}^{q}\right)}^{\omega_{i}}}{{\left(2-\min_{i}{{\underline{\xi }}_{i}}^{q}\right)}^{\omega_{i}}+\min_{i}{\left({{\underline{\xi }}_{i}}^{q}\right)}^{\omega_{i}}}}$$

Also, we have

$$\max_{i}{\bar{\xi }}_{i}\geq {\bar{\xi }}_{i}\geq \min_{i}{\bar{\xi }}_{i}$$

$$\max_{i}{{\bar{\xi }}_{i}}^{q}\geq {{\bar{\xi }}_{i}}^{q}\geq \min_{i}{{\bar{\xi }}_{i}}^{q}$$

$$\underset{i}{2\;\max}{\left({{\bar{\xi }}_{i}}^{q}\right)}^{\omega_{i}}\geq 2\prod_{j=1}^{n}\;{\left({{\bar{\xi }}_{i}}^{q}\right)}^{\omega_{i}}\geq \underset{i}{2\;\min}{\left({{\bar{\xi }}_{i}}^{q}\right)}^{\omega_{i}}$$

$$⟹\sqrt[{q}]{\frac{2\max_{i}{\left({{\bar{\xi }}_{i}}^{q}\right)}^{\omega_{i}}}{{\left(2-\max_{i}{{\bar{\xi }}_{i}}^{q}\right)}^{\omega_{i}}+\max_{i}{\left({{\bar{\xi }}_{i}}^{q}\right)}^{\omega_{i}}}}\geq \sqrt[{q}]{\frac{\underset{i}{2\prod_{i=1}^{n}\;}{\left({{\bar{\xi }}_{i}}^{q}\right)}^{\omega_{i}}}{{\left(2-{{\bar{\xi }}_{i}}^{q}\right)}^{\omega_{i}}+\prod_{i=1}^{n}\;{\left({{\bar{\xi }}_{i}}^{q}\right)}^{\omega_{i}}}\;}\geq \sqrt[{q}]{\frac{\underset{i}{2\;\min}{\left({{\bar{\xi }}_{i}}^{q}\right)}^{\omega_{i}}}{{\left(2-\min_{i}{{\bar{\xi }}_{i}}^{q}\right)}^{\omega_{i}}+\min_{i}{\left({{\bar{\xi }}_{i}}^{q}\right)}^{\omega_{i}}}}$$

Hence $A^{-}\leq q-SFREWA\left(A_{1},A_{2},\ldots ,A_{n}\right)\leq A^{+}\text{.}$

Theorem 5 (Monotonicity) *Assuming*$A_{i}=\left({\underline{\zeta }}_{i},{\underline{\eta }}_{i},{\underline{\xi }}_{i},{\bar{\zeta }}_{i},{\bar{\eta }}_{i},{\bar{\xi }}_{i}\right)\left(i=1,2,\ldots ,n\right)\;\text{and}\;{A_{i}}^{*}=\left({{\underline{\zeta }}_{i}}^{*},{{\underline{\eta }}_{i}}^{*},{{\underline{\xi }}_{i}}^{*},{{\bar{\zeta }}_{i}}^{*},{{\bar{\eta }}_{i}}^{*},{{\bar{\xi }}_{i}}^{*}\right)\left(i=1,2,\ldots ,n\right)$*be a collection of two*q−SFRNs*such that*$A_{i}\leq {A_{i}}^{*}$for all i,*then*$q-SFREWA\left(A_{1},A_{2},\ldots ,A_{n}\right)\leq q-SFREWA\left({A_{1}}^{*},{A_{2}}^{*},\ldots ,{A_{n}}^{*}\right)$.

**Proof:** From Theorem 2, we have

$$q-SFREWA\left(A_{1},A_{2},\ldots ,A_{n}\right)={{⊕}_{Ḛ}}_{i=1}^{n}\left(\omega_{i}A_{i}\right)=\left[\left\langle \begin{array}{c} \sqrt[{q}]{\frac{\prod_{i=1}^{n}\;{\left(1+{\underline{\zeta }}_{i}^{q}\right)}^{\omega_{i}}-\prod_{i=1}^{n}\;{\left(1-{\underline{\zeta }}_{i}^{q}\right)}^{\omega_{i}}}{\prod_{i=1}^{n}\;{\left(1+{\underline{\zeta }}_{i}^{q}\right)}^{\omega_{i}}+{\prod_{i=1}^{n}\;\left(1-{\underline{\zeta }}_{i}^{q}\right)}^{\omega_{i}}}},\sqrt[{q}]{\frac{2{\prod_{i=1}^{n}\;\left({\underline{\eta }}_{i}^{q}\right)}^{\omega_{i}}}{{\prod_{i=1}^{n}\;\left(2-{\underline{\eta }}_{i}^{q}\right)}^{\omega_{i}}+\prod_{i=1}^{n}\;{\left({\underline{\eta }}_{i}^{q}\right)}^{\omega_{i}}}},\sqrt[{q}]{\frac{2{\prod_{i=1}^{n}\;\left({\underline{\xi }}_{i}^{q}\right)}^{\omega_{i}}}{{\prod_{i=1}^{n}\;\left(2-{\underline{\xi }}_{i}^{q}\right)}^{\omega_{i}}+\prod_{i=1}^{n}\;{\left({\underline{\xi }}_{i}^{q}\right)}^{\omega_{i}}},}\\ \sqrt[{q}]{\frac{\prod_{i=1}^{n}\;{\left(1+{\bar{\zeta }}_{i}^{q}\right)}^{\omega_{i}}-\prod_{i=1}^{n}\;{\left(1-{\bar{\zeta }}_{i}^{q}\right)}^{\omega_{i}}}{\prod_{i=1}^{n}\;{\left(1+{\bar{\zeta }}_{i}^{q}\right)}^{\omega_{i}}+{\prod_{i=1}^{n}\;\left(1-{\bar{\zeta }}_{i}^{q}\right)}^{\omega_{i}}}},\sqrt[{q}]{\frac{2{\prod_{i=1}^{n}\;\left({\bar{\eta }}_{i}^{q}\right)}^{\omega_{i}}}{{\prod_{i=1}^{n}\;\left(2-{\bar{\eta }}_{i}^{q}\right)}^{\omega_{i}}+\prod_{i=1}^{n}\;{\left({\bar{\eta }}_{i}^{q}\right)}^{\omega_{i}}}},\sqrt[{q}]{\frac{2{\prod_{i=1}^{n}\;\left({\bar{\xi }}_{i}^{q}\right)}^{\omega_{i}}}{{\prod_{i=1}^{n}\;\left(2-{\bar{\xi }}_{i}^{q}\right)}^{\omega_{i}}+\prod_{i=1}^{n}\;{\left({\bar{\xi }}_{i}^{q}\right)}^{\omega_{i}}}} \end{array}\right\rangle \right]\;\text{and}$$

$$q-SFREWA\left({A_{1}}^{*},{A_{2}}^{*},\ldots ,{A_{n}}^{*}\right)={{⊕}_{Ḛ}}_{i=1}^{n}\left(\omega_{i}{A_{i}}^{*}\right)=\left[\left\langle \begin{array}{c} \sqrt[{q}]{\frac{\prod_{i=1}^{n}\;{\left(1+{{\underline{\zeta }}_{i}^{*}}^{q}\right)}^{\omega_{i}}-\prod_{i=1}^{n}\;{\left(1-{{\underline{\zeta }}_{i}^{*}}^{q}\right)}^{\omega_{i}}}{\prod_{i=1}^{n}\;{\left(1+{{\underline{\zeta }}_{i}^{*}}^{q}\right)}^{\omega_{i}}+{\prod_{i=1}^{n}\;\left(1-{{\underline{\zeta }}_{i}^{*}}^{q}\right)}^{\omega_{i}}}},\sqrt[{q}]{\frac{2{\prod_{i=1}^{n}\;\left({{\underline{\eta }}_{i}^{*}}^{q}\right)}^{\omega_{i}}}{{\prod_{i=1}^{n}\;\left(2-{{\underline{\eta }}_{i}^{*}}^{q}\right)}^{\omega_{i}}+\prod_{i=1}^{n}\;{\left({{\underline{\eta }}_{i}^{*}}^{q}\right)}^{\omega_{i}}}},\sqrt[{q}]{\frac{2{\prod_{i=1}^{n}\;\left({{\underline{\xi }}_{i}^{*}}^{q}\right)}^{\omega_{i}}}{{\prod_{i=1}^{n}\;\left(2-{{\underline{\xi }}_{i}^{*}}^{q}\right)}^{\omega_{i}}+\prod_{i=1}^{n}\;{\left({{\underline{\xi }}_{i}^{*}}^{q}\right)}^{\omega_{i}}},}\\ \sqrt[{q}]{\frac{\prod_{i=1}^{n}\;{\left(1+{{\bar{\zeta }}_{i}^{*}}^{q}\right)}^{\omega_{i}}-\prod_{i=1}^{n}\;{\left(1-{{\bar{\zeta }}_{i}^{*}}^{q}\right)}^{\omega_{i}}}{\prod_{i=1}^{n}\;{\left(1+{{\bar{\zeta }}_{i}^{*}}^{q}\right)}^{\omega_{i}}+{\prod_{i=1}^{n}\;\left(1-{{\bar{\zeta }}_{i}^{*}}^{q}\right)}^{\omega_{i}}}},\sqrt[{q}]{\frac{2{\prod_{i=1}^{n}\;\left({{\bar{\eta }}_{i}^{*}}^{q}\right)}^{\omega_{i}}}{{\prod_{i=1}^{n}\;\left(2-{{\bar{\eta }}_{i}^{*}}^{q}\right)}^{\omega_{i}}+\prod_{i=1}^{n}\;{\left({{\bar{\eta }}_{i}^{*}}^{q}\right)}^{\omega_{i}}}},\sqrt[{q}]{\frac{2{\prod_{i=1}^{n}\;\left({{\bar{\xi }}_{i}^{*}}^{q}\right)}^{\omega_{i}}}{{\prod_{i=1}^{n}\;\left(2-{{\bar{\xi }}_{i}^{*}}^{q}\right)}^{\omega_{i}}+\prod_{i=1}^{n}\;{\left({{\bar{\xi }}_{i}^{*}}^{q}\right)}^{\omega_{i}}}} \end{array}\right\rangle \right]$$

#### 3.2.4 For the lower and upper memberships

$$\text{As},{\underline{\zeta }}_{i}\leq {{\underline{\zeta }}_{i}}^{*}⟹{\underline{\zeta }}_{i}^{q}\leq {{\underline{\zeta }}_{i}^{*}}^{q}⟹1+{\underline{\zeta }}_{i}^{q}\leq 1+{{\underline{\zeta }}_{i}^{*}}^{q}⟹{\left({1+\underline{\zeta }}_{i}^{q}\right)}^{\omega_{i}}\leq {\left(1+{{\underline{\zeta }}_{i}^{*}}^{q}\right)}^{\omega_{i}}⟹{\prod_{i=1}^{n}\;\left({1+\underline{\zeta }}_{i}^{q}\right)}^{\omega_{i}}\leq {\prod_{i=1}^{n}\;\left(1+{{\underline{\zeta }}_{i}^{*}}^{q}\right)}^{\omega_{i}}$$

$$\sqrt[{q}]{\frac{\prod_{i=1}^{n}\;{\left(1+{\underline{\zeta }}_{i}^{q}\right)}^{\omega_{i}}-\prod_{j=1}^{n}\;{\left(1-{\underline{\zeta }}_{i}^{q}\right)}^{\omega_{i}}}{\prod_{i=1}^{n}\;{\left(1+{\underline{\zeta }}_{i}^{q}\right)}^{\omega_{i}}+{\prod_{j=1}^{n}\;\left(1-{\underline{\zeta }}_{i}^{q}\right)}^{\omega_{i}}}}\leq \sqrt[{q}]{\frac{\prod_{i=1}^{n}\;{\left(1+{{\underline{\zeta }}_{i}^{*}}^{q}\right)}^{\omega_{i}}-\prod_{i=1}^{n}\;{\left(1-{{\underline{\zeta }}_{i}^{*}}^{q}\right)}^{\omega_{i}}}{\prod_{i=1}^{n}\;{\left(1+{{\underline{\zeta }}_{i}^{*}}^{q}\right)}^{\omega_{i}}+{\prod_{i=1}^{n}\;\left(1-{{\underline{\zeta }}_{i}^{*}}^{q}\right)}^{\omega_{i}}}\;}$$

$\text{and}\;{\bar{\zeta }}_{i}\leq {{\bar{\zeta }}_{i}}^{*}$ for all i then ${\bar{\zeta }}_{i}^{q}\leq {{\bar{\zeta }}_{i}^{*}}^{q}⟹{1+\bar{\zeta }}_{i}^{q}\leq 1+{{\bar{\zeta }}_{i}^{*}}^{q}⟹{\left({1+\bar{\zeta }}_{i}^{q}\right)}^{\omega_{i}}\leq {\left(1+{{\bar{\zeta }}_{i}^{*}}^{q}\right)}^{\omega_{i}}⟹{\prod_{i=1}^{n}\;\left({1+\bar{\zeta }}_{i}^{q}\right)}^{\omega_{i}}\leq {\prod_{i=1}^{n}\;\left(1+{{\bar{\zeta }}_{i}^{*}}^{q}\right)}^{\omega_{i}}$.

$$\sqrt[{q}]{\frac{\prod_{i=1}^{n}\;{\left({1+\bar{\zeta }}_{i}^{q}\right)}^{\omega_{i}}-\prod_{i=1}^{n}\;{\left({1-\bar{\zeta }}_{i}^{q}\right)}^{\omega_{i}}}{\prod_{i=1}^{n}\;{\left({1+\bar{\zeta }}_{i}^{q}\right)}^{\omega_{i}}+{\prod_{i=1}^{n}\;\left({1-\bar{\zeta }}_{i}^{q}\right)}^{\omega_{i}}}}\leq \sqrt[{q}]{\frac{\prod_{i=1}^{n}\;{\left(1+{{\bar{\zeta }}_{i}^{*}}^{q}\right)}^{\omega_{i}}-\prod_{i=1}^{n}\;{\left(1-{{\bar{\zeta }}_{i}^{*}}^{q}\right)}^{\omega_{i}}}{\prod_{i=1}^{n}\;{\left(1+{{\bar{\zeta }}_{i}^{*}}^{q}\right)}^{\omega_{i}}+{\prod_{i=1}^{n}\;\left(1-{{\bar{\zeta }}_{i}^{*}}^{q}\right)}^{\omega_{i}}}\;}$$

#### 3.2.5 For the lower and upper neutral memberships

$$\text{As},{\underline{\eta }}_{i}\geq {{\underline{\eta }}_{i}}^{*}\;⟹{\underline{\eta }}_{i}^{q}\geq {{\underline{\eta }}_{i}^{*}}^{q}⟹{\left({\underline{\eta }}_{i}^{q}\right)}^{\omega_{i}}\geq {\left({{\underline{\eta }}_{i}^{*}}^{q}\right)}^{\omega_{i}}⟹2\prod_{i=1}^{n}\;{\left({\underline{\eta }}_{i}^{q}\right)}^{\omega_{i}}\geq {2\prod_{i=1}^{n}\;\left({{\underline{\eta }}_{i}^{*}}^{q}\right)}^{\omega_{i}}$$

$$\sqrt[{q}]{\frac{2\prod_{i=1}^{n}\;{\left({\underline{\eta }}_{i}^{q}\right)}^{\omega_{i}}}{{\prod_{i=1}^{n}\;\left(2-{\underline{\eta }}_{i}^{q}\right)}^{\omega_{i}}+\prod_{i=1}^{n}\;{\left({\underline{\eta }}_{i}^{q}\right)}^{\omega_{i}}}}\leq \sqrt[{q}]{\frac{2\prod_{i=1}^{n}\;{\left({{\underline{\eta }}_{i}^{*}}^{q}\right)}^{\omega_{i}}}{{\prod_{i=1}^{n}\;\left(2-{{\underline{\eta }}_{i}^{*}}^{q}\right)}^{\omega_{i}}+\prod_{i=1}^{n}\;{\left({{\underline{\eta }}_{i}^{*}}^{q}\right)}^{\omega_{i}}}}$$

$$\text{And},{\bar{\eta }}_{i}\geq {{\bar{\eta }}_{i}}^{*}\;⟹{\bar{\eta }}_{i}^{q}\geq {{\bar{\eta }}_{i}^{*}}^{q}⟹{\left({\bar{\eta }}_{i}^{q}\right)}^{\omega_{i}}\geq {\left({{\bar{\eta }}_{i}^{*}}^{q}\right)}^{\omega_{i}}⟹2\prod_{i=1}^{n}\;{\left({\bar{\eta }}_{i}^{q}\right)}^{\omega_{i}}\geq {2\prod_{i=1}^{n}\;\left({{\bar{\eta }}_{i}^{*}}^{q}\right)}^{\omega_{i}}$$

$$\sqrt[{q}]{\frac{2\prod_{i=1}^{n}\;{\left({\bar{\eta }}_{i}^{q}\right)}^{\omega_{i}}}{{\prod_{i=1}^{n}\;\left(2-{\bar{\eta }}_{i}^{q}\right)}^{\omega_{i}}+\prod_{i=1}^{n}\;{\left({\bar{\eta }}_{i}^{q}\right)}^{\omega_{i}}}}\geq \sqrt[{q}]{\frac{2\prod_{i=1}^{n}\;{\left({{\bar{\eta }}_{i}^{*}}^{q}\right)}^{\omega_{i}}}{{\prod_{i=1}^{n}\;\left(2-{{\bar{\eta }}_{i}^{*}}^{q}\right)}^{\omega_{i}}+\prod_{i=1}^{n}\;{\left({{\bar{\eta }}_{i}^{*}}^{q}\right)}^{\omega_{i}}}}$$

#### 3.2.6 For the lower and upper non-memberships

$$\text{As},{\underline{\xi }}_{i}\geq {{\underline{\xi }}_{i}}^{*}\;⟹{\underline{\xi }}_{i}^{q}\geq {{\underline{\xi }}_{i}^{*}}^{q}⟹{\left({\underline{\xi }}_{i}^{q}\right)}^{\omega_{i}}\geq {\left({{\underline{\xi }}_{i}^{*}}^{q}\right)}^{\omega_{i}}⟹2\prod_{i=1}^{n}\;{\left({\underline{\xi }}_{i}^{q}\right)}^{\omega_{i}}\geq {2\prod_{i=1}^{n}\;\left({{\underline{\xi }}_{i}^{*}}^{q}\right)}^{\omega_{i}}$$

$$\sqrt[{q}]{\frac{2\prod_{i=1}^{n}\;{\left({\underline{\xi }}_{i}^{q}\right)}^{\omega_{i}}}{{\prod_{i=1}^{n}\;\left(2-{\underline{\xi }}_{i}^{q}\right)}^{\omega_{i}}+\prod_{i=1}^{n}\;{\left({\underline{\xi }}_{i}^{q}\right)}^{\omega_{i}}}}\leq \sqrt[{q}]{\frac{2\prod_{i=1}^{n}\;{\left({{\underline{\xi }}_{i}^{*}}^{q}\right)}^{\omega_{i}}}{{\prod_{i=1}^{n}\;\left(2-{{\underline{\xi }}_{i}^{*}}^{q}\right)}^{\omega_{i}}+\prod_{i=1}^{n}\;{\left({{\underline{\xi }}_{i}^{*}}^{q}\right)}^{\omega_{i}}}}$$

And, ${\bar{\xi }}_{i}\geq {{\bar{\xi }}_{i}}^{*}$ ⟹ ${\bar{\xi }}_{i}^{q}\geq {{\bar{\xi }}_{i}^{*}}^{q}⟹{\left({\bar{\xi }}_{i}^{q}\right)}^{\omega_{i}}\geq {\left({{\bar{\xi }}_{i}^{*}}^{q}\right)}^{\omega_{i}}⟹2\prod_{i=1}^{n}\;{\left({\bar{\xi }}_{i}^{q}\right)}^{\omega_{i}}\geq {2\prod_{i=1}^{n}\;\left({{\bar{\xi }}_{i}^{*}}^{q}\right)}^{\omega_{i}}$.

$$\sqrt[{q}]{\frac{2\prod_{i=1}^{n}\;{\left({\bar{\xi }}_{i}^{q}\right)}^{\omega_{i}}}{{\prod_{i=1}^{n}\;\left(2-{\bar{\xi }}_{i}^{q}\right)}^{\omega_{i}}+\prod_{i=1}^{n}\;{\left({\bar{\xi }}_{i}^{q}\right)}^{\omega_{i}}}}\geq \sqrt[{q}]{\frac{2\prod_{i=1}^{n}\;{\left({{\bar{\xi }}_{i}^{*}}^{q}\right)}^{\omega_{i}}}{{\prod_{i=1}^{n}\;\left(2-{{\bar{\xi }}_{i}^{*}}^{q}\right)}^{\omega_{i}}+\prod_{i=1}^{n}\;{\left({{\bar{\xi }}_{i}^{*}}^{q}\right)}^{\omega_{i}}}}$$

### 3.3 Some specific cases regarding the q−SFREWAoperator

From Theorem 2, we have

$$q-SFREWA\left(A_{1},A_{2},\ldots ,A_{n}\right)={{⊕}_{Ḛ}}_{i=1}^{n}\left(\omega_{i}A_{i}\right)=\left[\left\langle \begin{array}{c} \sqrt[{q}]{\frac{\prod_{i=1}^{n}\;{\left(1+{\underline{\zeta }}_{i}^{q}\right)}^{\omega_{i}}-\prod_{i=1}^{n}\;{\left(1-{\underline{\zeta }}_{i}^{q}\right)}^{\omega_{i}}}{\prod_{i=1}^{n}\;{\left(1+{\underline{\zeta }}_{i}^{q}\right)}^{\omega_{i}}+{\prod_{i=1}^{n}\;\left(1-{\underline{\zeta }}_{i}^{q}\right)}^{\omega_{i}}}},\sqrt[{q}]{\frac{2{\prod_{i=1}^{n}\;\left({\underline{\eta }}_{i}^{q}\right)}^{\omega_{i}}}{{\prod_{i=1}^{n}\;\left(2-{\underline{\eta }}_{i}^{q}\right)}^{\omega_{i}}+\prod_{i=1}^{n}\;{\left({\underline{\eta }}_{i}^{q}\right)}^{\omega_{i}}}},\sqrt[{q}]{\frac{2{\prod_{i=1}^{n}\;\left({\underline{\xi }}_{i}^{q}\right)}^{\omega_{i}}}{{\prod_{i=1}^{n}\;\left(2-{\underline{\xi }}_{i}^{q}\right)}^{\omega_{i}}+\prod_{i=1}^{n}\;{\left({\underline{\xi }}_{i}^{q}\right)}^{\omega_{i}}},}\\ \sqrt[{q}]{\frac{\prod_{i=1}^{n}\;{\left(1+{\bar{\zeta }}_{i}^{q}\right)}^{\omega_{i}}-\prod_{i=1}^{n}\;{\left(1-{\bar{\zeta }}_{i}^{q}\right)}^{\omega_{i}}}{\prod_{i=1}^{n}\;{\left(1+{\bar{\zeta }}_{i}^{q}\right)}^{\omega_{i}}+{\prod_{i=1}^{n}\;\left(1-{\bar{\zeta }}_{i}^{q}\right)}^{\omega_{i}}}},\sqrt[{q}]{\frac{2{\prod_{i=1}^{n}\;\left({\bar{\eta }}_{i}^{q}\right)}^{\omega_{i}}}{{\prod_{i=1}^{n}\;\left(2-{\bar{\eta }}_{i}^{q}\right)}^{\omega_{i}}+\prod_{i=1}^{n}\;{\left({\bar{\eta }}_{i}^{q}\right)}^{\omega_{i}}}},\sqrt[{q}]{\frac{2{\prod_{i=1}^{n}\;\left({\bar{\xi }}_{i}^{q}\right)}^{\omega_{i}}}{{\prod_{i=1}^{n}\;\left(2-{\bar{\xi }}_{i}^{q}\right)}^{\omega_{i}}+\prod_{i=1}^{n}\;{\left({\bar{\xi }}_{i}^{q}\right)}^{\omega_{i}}}} \end{array}\right\rangle \right]$$

We are facing the following cases.

Case 1 If ${\underline{\zeta }}_{i}={\underline{\eta }}_{i}={\underline{\xi }}_{i}=0\;and\;q=2$ then

$$q-SFREWA\left(A_{1},A_{2},\ldots ,A_{n}\right)={{⊕}_{Ḛ}}_{i=1}^{n}\left(\omega_{i}A_{i}\right)=\left[\left\langle \sqrt{\frac{\prod_{i=1}^{n}\;{\left(1+{\bar{\zeta }}_{i}^{2}\right)}^{\omega_{i}}-\prod_{i=1}^{n}\;{\left(1-{\bar{\zeta }}_{i}^{2}\right)}^{\omega_{i}}}{\prod_{i=1}^{n}\;{\left(1+{\bar{\zeta }}_{i}^{2}\right)}^{\omega_{i}}+{\prod_{i=1}^{n}\;\left(1-{\bar{\zeta }}_{i}^{2}\right)}^{\omega_{i}}}},\sqrt{\frac{2{\prod_{i=1}^{n}\;\left({\bar{\eta }}_{i}^{2}\right)}^{\omega_{i}}}{{\prod_{i=1}^{n}\;\left(2-{\bar{\eta }}_{i}^{2}\right)}^{\omega_{i}}+\prod_{i=1}^{n}\;{\left({\bar{\eta }}_{i}^{2}\right)}^{\omega_{i}}}},\sqrt{\frac{2{\prod_{i=1}^{n}\;\left({\bar{\xi }}_{i}^{2}\right)}^{\omega_{i}}}{{\prod_{i=1}^{n}\;\left(2-{\bar{\xi }}_{i}^{2}\right)}^{\omega_{i}}+\prod_{i=1}^{n}\;{\left({\bar{\xi }}_{i}^{2}\right)}^{\omega_{i}}}}\right\rangle \right]$$

$$=SFEWA\left(A_{1},A_{2},\ldots ,A_{n}\right)={{⊕}_{Ḛ}}_{i=1}^{n}\left(\omega_{i}A_{i}\right)$$

(The spherical fuzzy rough Einstein weighted averaging operator).

Case 2 If ${\underline{\xi }}_{i}={\bar{\xi }}_{i}=0\;and\;q=2$ then

$$q-SFREWA\left(A_{1},A_{2},\ldots ,A_{n}\right)={{⊕}_{Ḛ}}_{i=1}^{n}\left(\omega_{i}A_{i}\right)=\left[\left\langle \begin{array}{c} \sqrt{\frac{\prod_{i=1}^{n}\;{\left(1+{\underline{\zeta }}_{i}^{2}\right)}^{\omega_{i}}-\prod_{i=1}^{n}\;{\left(1-{\underline{\zeta }}_{i}^{2}\right)}^{\omega_{i}}}{\prod_{i=1}^{n}\;{\left(1+{\underline{\zeta }}_{i}^{2}\right)}^{\omega_{i}}+{\prod_{i=1}^{n}\;\left(1-{\underline{\zeta }}_{i}^{2}\right)}^{\omega_{i}}}},\sqrt[{q}]{\frac{2{\prod_{i=1}^{n}\;\left({\underline{\eta }}_{i}^{q}\right)}^{\omega_{i}}}{{\prod_{i=1}^{n}\;\left(2-{\underline{\eta }}_{i}^{q}\right)}^{\omega_{i}}+\prod_{i=1}^{n}\;{\left({\underline{\eta }}_{i}^{q}\right)}^{\omega_{i}}},}\\ \sqrt{\frac{\prod_{i=1}^{n}\;{\left(1+{\bar{\zeta }}_{i}^{2}\right)}^{\omega_{i}}-\prod_{i=1}^{n}\;{\left(1-{\bar{\zeta }}_{i}^{2}\right)}^{\omega_{i}}}{\prod_{i=1}^{n}\;{\left(1+{\bar{\zeta }}_{i}^{2}\right)}^{\omega_{i}}+{\prod_{i=1}^{n}\;\left(1-{\bar{\zeta }}_{i}^{2}\right)}^{\omega_{i}}}},\sqrt[{q}]{\frac{2{\prod_{i=1}^{n}\;\left({\bar{\eta }}_{i}^{2}\right)}^{\omega_{i}}}{{\prod_{i=1}^{n}\;\left(2-{\bar{\eta }}_{i}^{2}\right)}^{\omega_{i}}+\prod_{i=1}^{n}\;{\left({\bar{\eta }}_{i}^{2}\right)}^{\omega_{i}}}} \end{array}\right\rangle \right]$$

$$=PyFREWA\left(A_{1},A_{2},\ldots ,A_{n}\right)={{⊕}_{Ḛ}}_{i=1}^{n}\left(\omega_{i}A_{i}\right)$$

(The Pythagorean fuzzy rough Einstein weighted averaging operator).

Case 3 If q=1 then

$$q-SFREWA\left(A_{1},A_{2},\ldots ,A_{n}\right)={{⊕}_{Ḛ}}_{i=1}^{n}\left(\omega_{i}A_{i}\right)=\left[\left\langle \begin{array}{c} \frac{\prod_{i=1}^{n}\;{\left(1+{\underline{\zeta }}_{i}\right)}^{\omega_{i}}-\prod_{i=1}^{n}\;{\left(1-{\underline{\zeta }}_{i}\right)}^{\omega_{i}}}{\prod_{i=1}^{n}\;{\left(1+{\underline{\zeta }}_{i}\right)}^{\omega_{i}}+{\prod_{i=1}^{n}\;\left(1-{\underline{\zeta }}_{i}\right)}^{\omega_{i}}},\frac{2{\prod_{i=1}^{n}\;\left({\underline{\eta }}_{i}\right)}^{\omega_{i}}}{{\prod_{i=1}^{n}\;\left(2-{\underline{\eta }}_{i}\right)}^{\omega_{i}}+\prod_{i=1}^{n}\;{\left({\underline{\eta }}_{i}\right)}^{\omega_{i}}},\frac{2{\prod_{i=1}^{n}\;\left({\underline{\xi }}_{i}\right)}^{\omega_{i}}}{{\prod_{i=1}^{n}\;\left(2-{\underline{\xi }}_{i}\right)}^{\omega_{i}}+\prod_{i=1}^{n}\;{\left({\underline{\xi }}_{i}\right)}^{\omega_{i}}},\\ \frac{\prod_{i=1}^{n}\;{\left(1+{\bar{\zeta }}_{i}\right)}^{\omega_{i}}-\prod_{i=1}^{n}\;{\left(1-{\bar{\zeta }}_{i}\right)}^{\omega_{i}}}{\prod_{i=1}^{n}\;{\left(1+{\bar{\zeta }}_{i}\right)}^{\omega_{i}}+{\prod_{i=1}^{n}\;\left(1-{\bar{\zeta }}_{i}\right)}^{\omega_{i}}},\frac{2{\prod_{i=1}^{n}\;\left({\bar{\eta }}_{i}\right)}^{\omega_{i}}}{{\prod_{i=1}^{n}\;\left(2-{\bar{\eta }}_{i}\right)}^{\omega_{i}}+\prod_{i=1}^{n}\;{\left({\bar{\eta }}_{i}\right)}^{\omega_{i}}},\frac{2{\prod_{i=1}^{n}\;\left({\bar{\xi }}_{i}\right)}^{\omega_{i}}}{{\prod_{i=1}^{n}\;\left(2-{\bar{\xi }}_{i}\right)}^{\omega_{i}}+\prod_{i=1}^{n}\;{\left({\bar{\xi }}_{i}\right)}^{\omega_{i}}} \end{array}\right\rangle \right]$$

$$=PFREWA\left(A_{1},A_{2},\ldots ,A_{n}\right)={{⊕}_{Ḛ}}_{i=1}^{n}\left(\omega_{i}A_{i}\right)$$

(The picture fuzzy rough Einstein weighted averaging operator).

Case 4 If ${\underline{\xi }}_{i}={\bar{\xi }}_{i}=0\;and\;q=1$ then

$$q-SFREWA\left(A_{1},A_{2},\ldots ,A_{n}\right)={⊕}_{i=1}^{n}\left(\omega_{i}A_{i}\right)=\left[\left\langle \begin{array}{c} \frac{\prod_{i=1}^{n}\;{\left(1+{\underline{\zeta }}_{i}\right)}^{\omega_{i}}-\prod_{i=1}^{n}\;{\left(1-{\underline{\zeta }}_{i}\right)}^{\omega_{i}}}{\prod_{i=1}^{n}\;{\left(1+{\underline{\zeta }}_{i}\right)}^{\omega_{i}}+{\prod_{i=1}^{n}\;\left(1-{\underline{\zeta }}_{i}\right)}^{\omega_{i}}},\frac{2{\prod_{i=1}^{n}\;\left({\underline{\eta }}_{i}\right)}^{\omega_{i}}}{{\prod_{i=1}^{n}\;\left(2-{\underline{\eta }}_{i}\right)}^{\omega_{i}}+\prod_{i=1}^{n}\;{\left({\underline{\eta }}_{i}\right)}^{\omega_{i}}},\\ \frac{\prod_{i=1}^{n}\;{\left(1+{\bar{\zeta }}_{i}\right)}^{\omega_{i}}-\prod_{i=1}^{n}\;{\left(1-{\bar{\zeta }}_{i}\right)}^{\omega_{i}}}{\prod_{i=1}^{n}\;{\left(1+{\bar{\zeta }}_{i}\right)}^{\omega_{i}}+{\prod_{i=1}^{n}\;\left(1-{\bar{\zeta }}_{i}\right)}^{\omega_{i}}},\frac{2{\prod_{i=1}^{n}\;\left({\bar{\eta }}_{i}\right)}^{\omega_{i}}}{{\prod_{i=1}^{n}\;\left(2-{\bar{\eta }}_{i}\right)}^{\omega_{i}}+\prod_{i=1}^{n}\;{\left({\bar{\eta }}_{i}\right)}^{\omega_{i}}}, \end{array}\right\rangle \right]$$

$$=IFREWA\left(A_{1},A_{2},\ldots ,A_{n}\right)={{⊕}_{Ḛ}}_{i=1}^{n}\left(\omega_{i}A_{i}\right)$$

(The intuitionistic fuzzy rough Einstein weighted averaging operator).

**Fig. 4:**
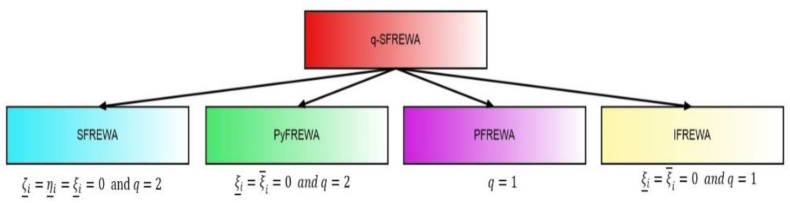
Specificcasesregardingtheq−SFREWAoperator.

Fig. 4 represents the specificcasesregarding the q−SFREWAoperator.

### 3.4 q−SFREOWAoperator

Definition 15 Assuming $A_{i}=\left({\underline{\zeta }}_{i},{\underline{\eta }}_{i},{\underline{\xi }}_{i},{\bar{\zeta }}_{i},{\bar{\eta }}_{i},{\bar{\xi }}_{i}\right)\left(i=1,2,\ldots ,n\right)$ ne a collection of q-SFRNs, the q-spherical fuzzy rough Einstein ordered averaging operator (q-SFREOWA) operator is defined as a mapping $q-SFREOWA:A^{n}⟶A$ associated with the weight vector ${\left(\omega_{1},\omega_{2},\ldots ,\omega_{n}\right)}^{T}$ adhering the condition $\omega_{i}>0$ and the constrain $\sum_{i=1}^{n}\omega_{i}=1$.

$$q-SFREOWA\left(A_{\delta \left(1\right)},A_{\delta \left(2\right)},\ldots ,A_{\delta \left(n\right)}\right)=A_{\delta \left(1\right)}{⊕}_{Ḛ}A_{\delta \left(2\right)},\ldots ,{⊕}_{Ḛ}A_{\delta \left(n\right)}={{⊕}_{Ḛ}}_{i=1}^{n}\left(\omega_{i}A_{\delta \left(i\right)}\right)$$

Where δ(1),δ(2),…,δ(n) is a permutation of (1,2,3,‥,n) such that $A_{\delta \left(1\right)}\leq A_{\delta \left(i-1\right)}$ for all i=1,2,3,‥,n.

Now,

Given $A_{i}=\left({\underline{\zeta }}_{i},{\underline{\eta }}_{i},{\underline{\xi }}_{i},{\bar{\zeta }}_{i},{\bar{\eta }}_{i},{\bar{\xi }}_{i}\right)$ for (i=1,2,…,n) as a collection of q-SFRNs, and ${\omega =\left(\omega_{1},\omega_{2},\ldots ,\omega_{n}\right)}^{T}$ is a weight vector satisfying $\omega_{i}>0$ and $\sum_{i=1}^{n}\omega_{i}=1\text{.}$ Then

$$q-SFREOWA\left(A_{\delta \left(1\right)},A_{\delta \left(2\right)},\ldots ,A_{\delta \left(n\right)}\right)=\left[\left\langle \begin{array}{c} \sqrt[{q}]{\frac{\prod_{i=1}^{n}\;{\left(1+{\underline{\zeta }}_{\delta \left(i\right)}^{q}\right)}^{\omega_{i}}-\prod_{i=1}^{n}\;{\left(1-{\underline{\zeta }}_{\delta \left(i\right)}^{q}\right)}^{\omega_{i}}}{\prod_{i=1}^{n}\;{\left(1+{\underline{\zeta }}_{\delta \left(i\right)}^{q}\right)}^{\omega_{i}}+{\prod_{i=1}^{n}\;\left(1-{\underline{\zeta }}_{\delta \left(i\right)}^{q}\right)}^{\omega_{i}}}},\sqrt[{q}]{\frac{2{\prod_{i=1}^{n}\;\left({\underline{\eta }}_{\delta \left(i\right)}^{q}\right)}^{\omega_{i}}}{{\prod_{i=1}^{n}\;\left(2-{\underline{\eta }}_{\delta \left(i\right)}^{q}\right)}^{\omega_{i}}+\prod_{i=1}^{n}\;{\left({\underline{\eta }}_{\delta \left(i\right)}^{q}\right)}^{\omega_{i}}}},\sqrt[{q}]{\frac{2{\prod_{i=1}^{n}\;\left(\;{\underline{\xi }}_{\delta \left(i\right)}^{q}\right)}^{\omega_{i}}}{{\prod_{i=1}^{n}\;\left(2-\;{\underline{\xi }}_{\delta \left(i\right)}^{q}\right)}^{\omega_{i}}+\prod_{i=1}^{n}\;{\left(\;{\underline{\xi }}_{\delta \left(i\right)}^{q}\right)}^{\omega_{i}}},}\\ \sqrt[{q}]{\frac{\prod_{i=1}^{n}\;{\left(1+{\bar{\zeta }}_{\delta \left(i\right)}^{q}\right)}^{\omega_{i}}-\prod_{i=1}^{n}\;{\left(1-{\bar{\zeta }}_{\delta \left(i\right)}^{q}\right)}^{\omega_{i}}}{\prod_{i=1}^{n}\;{\left(1+{\bar{\zeta }}_{\delta \left(i\right)}^{q}\right)}^{\omega_{i}}+{\prod_{i=1}^{n}\;\left(1-{\bar{\zeta }}_{\delta \left(i\right)}^{q}\right)}^{\omega_{i}}}},\sqrt[{q}]{\frac{2{\prod_{i=1}^{n}\;\left({\bar{\eta }}_{\delta \left(i\right)}^{q}\right)}^{\omega_{i}}}{{\prod_{i=1}^{n}\;\left(2-{\bar{\eta }}_{\delta \left(i\right)}^{q}\right)}^{\omega_{i}}+\prod_{i=1}^{n}\;{\left({\bar{\eta }}_{\delta \left(i\right)}^{q}\right)}^{\omega_{i}}}},\sqrt[{q}]{\frac{2{\prod_{i=1}^{n}\;\left(\;{\bar{\xi }}_{\delta \left(i\right)}^{q}\right)}^{\omega_{i}}}{{\prod_{i=1}^{n}\;\left(2-\;{\bar{\xi }}_{\delta \left(i\right)}^{q}\right)}^{\omega_{i}}+\prod_{i=1}^{n}\;{\left(\;{\bar{\xi }}_{\delta \left(i\right)}^{q}\right)}^{\omega_{i}}}} \end{array}\right\rangle \right]$$

Theorem 6 *Let*$A_{i}=\left({\underline{\zeta }}_{i},{\underline{\eta }}_{i},{\underline{\xi }}_{i},{\bar{\zeta }}_{i},{\bar{\eta }}_{i},{\bar{\xi }}_{i}\right)$*for*(i=1,2,…,n)*be a collection of q-SFRNs*, *and*${\omega =\left(\omega_{1},\omega_{2},\ldots ,\omega_{n}\right)}^{T}$*be the weight vector satisfying*$\omega_{i}>0$*and*$\sum_{i=1}^{n}\omega_{i}=1\text{.}$*If these conditions are met*, *the operator is referred to as the q-SFREOWA operator*.

$$q-SFREOWA\left(A_{\delta \left(1\right)},A_{\delta \left(2\right)},\ldots ,A_{\delta \left(n\right)}\right)=\left[\left\langle \begin{array}{c} \sqrt[{q}]{\frac{\prod_{i=1}^{n}\;{\left(1+{\underline{\zeta }}_{\delta \left(i\right)}^{q}\right)}^{\omega_{i}}-\prod_{i=1}^{n}\;{\left(1-{\underline{\zeta }}_{\delta \left(i\right)}^{q}\right)}^{\omega_{i}}}{\prod_{i=1}^{n}\;{\left(1+{\underline{\zeta }}_{\delta \left(i\right)}^{q}\right)}^{\omega_{i}}+{\prod_{i=1}^{n}\;\left(1-{\underline{\zeta }}_{\delta \left(i\right)}^{q}\right)}^{\omega_{i}}}},\sqrt[{q}]{\frac{2{\prod_{i=1}^{n}\;\left({\underline{\eta }}_{\delta \left(i\right)}^{q}\right)}^{\omega_{i}}}{{\prod_{i=1}^{n}\;\left(2-{\underline{\eta }}_{\delta \left(i\right)}^{q}\right)}^{\omega_{i}}+\prod_{i=1}^{n}\;{\left({\underline{\eta }}_{\delta \left(i\right)}^{q}\right)}^{\omega_{i}}}},\sqrt[{q}]{\frac{2{\prod_{i=1}^{n}\;\left(\;{\underline{\xi }}_{\delta \left(i\right)}^{q}\right)}^{\omega_{i}}}{{\prod_{i=1}^{n}\;\left(2-\;{\underline{\xi }}_{\delta \left(i\right)}^{q}\right)}^{\omega_{i}}+\prod_{i=1}^{n}\;{\left(\;{\underline{\xi }}_{\delta \left(i\right)}^{q}\right)}^{\omega_{i}}},}\\ \sqrt[{q}]{\frac{\prod_{i=1}^{n}\;{\left(1+{\bar{\zeta }}_{\delta \left(i\right)}^{q}\right)}^{\omega_{i}}-\prod_{i=1}^{n}\;{\left(1-{\bar{\zeta }}_{\delta \left(i\right)}^{q}\right)}^{\omega_{i}}}{\prod_{i=1}^{n}\;{\left(1+{\bar{\zeta }}_{\delta \left(i\right)}^{q}\right)}^{\omega_{i}}+{\prod_{i=1}^{n}\;\left(1-{\bar{\zeta }}_{\delta \left(i\right)}^{q}\right)}^{\omega_{i}}}},\sqrt[{q}]{\frac{2{\prod_{i=1}^{n}\;\left({\bar{\eta }}_{\delta \left(i\right)}^{q}\right)}^{\omega_{i}}}{{\prod_{i=1}^{n}\;\left(2-{\bar{\eta }}_{\delta \left(i\right)}^{q}\right)}^{\omega_{i}}+\prod_{i=1}^{n}\;{\left({\bar{\eta }}_{\delta \left(i\right)}^{q}\right)}^{\omega_{i}}}},\sqrt[{q}]{\frac{2{\prod_{i=1}^{n}\;\left(\;{\bar{\xi }}_{\delta \left(i\right)}^{q}\right)}^{\omega_{i}}}{{\prod_{i=1}^{n}\;\left(2-\;{\bar{\xi }}_{\delta \left(i\right)}^{q}\right)}^{\omega_{i}}+\prod_{i=1}^{n}\;{\left(\;{\bar{\xi }}_{\delta \left(i\right)}^{q}\right)}^{\omega_{i}}}} \end{array}\right\rangle \right]$$

**Proof:** The proof follows the same steps as in Theorem 2.

Theorem 7 (Idempotency) *Assuming*$A_{i}=\left({\underline{\zeta }}_{i},{\underline{\eta }}_{i},{\underline{\xi }}_{i},{\bar{\zeta }}_{i},{\bar{\eta }}_{i},{\bar{\xi }}_{i}\right)\left(i=1,2,\ldots ,n\right)$*be a collection of*$q-SFRNs\;\text{and}\;{\left(\omega_{1},\omega_{2},\ldots ,\omega_{n}\right)}^{T}$*signifies the weight vector adhering to the condition*$\omega_{i}>0$*and the constrain*$\sum_{i=1}^{n}\omega_{i}=1\text{.}$$A_{i}\left(i=1,2,\ldots ,n\right)$*are the same*∀i,*then*

$$q-SFREOWA\left(A_{\delta \left(1\right)},A_{\delta \left(2\right)},\ldots ,A_{\delta \left(n\right)}\right)=A$$

**Proof:** The proof follows the same steps as in Theorem 3.

Theorem 8 (Boundness) *Assuming*$A_{i}=\left({\underline{\zeta }}_{i},{\underline{\eta }}_{i},{\underline{\xi }}_{i},{\bar{\zeta }}_{i},{\bar{\eta }}_{i},{\bar{\xi }}_{i}\right)\left(i=1,2,\ldots ,n\right)$*be a collection of*$q-SFRNs\;\text{and}\;{\left(\omega_{1},\omega_{2},\ldots ,\omega_{n}\right)}^{T}$*signifies the weight vector adhering to the condition*$\omega_{i}>0$*and the constrain*$\sum_{i=1}^{n}\omega_{i}=1$.

$$\text{Let}\;A^{-}=\left(\min\;{\underline{\zeta }}_{i},\max\;{\underline{\eta }}_{i},\max\;{\underline{\xi }}_{i},\min\;{\bar{\zeta }}_{i},\max\;{\bar{\eta }}_{i},\max\;{\bar{\xi }}_{i}\;\right)\;\text{and}\;A^{+}=\left(\max\;{\underline{\zeta }}_{i},\min\;{\underline{\eta }}_{i},\min\;{\underline{\xi }}_{i},\max\;{\bar{\zeta }}_{i},\min\;{\bar{\eta }}_{i},\min\;{\bar{\xi }}_{i}\right)$$

$$\text{Then}\;A^{-}\leq q-SFREOWA\left(A_{\delta \left(1\right)},A_{\delta \left(2\right)},\ldots ,A_{\delta \left(n\right)}\right)\leq A^{+}$$

**Proof:** The proof follows the same steps as in Theorem 4.

Theorem 9 (Monotonicity) *Let*$A_{i}=\left({\underline{\zeta }}_{i},{\underline{\eta }}_{i},{\underline{\xi }}_{i},{\bar{\zeta }}_{i},{\bar{\eta }}_{i},{\bar{\xi }}_{i}\right)$*and*${A_{i}}^{*}=\left({{\underline{\zeta }}_{i}}^{*},{{\underline{\eta }}_{i}}^{*},{{\underline{\xi }}_{i}}^{*},{{\bar{\zeta }}_{i}}^{*},{{\bar{\eta }}_{i}}^{*},{{\bar{\xi }}_{i}}^{*}\right)$*for*(i=1,2,…,n)*be a collection of two q-SFRNs such that*$A_{i}\leq {A_{i}}^{*}$for all i. *Then*, $q-SFREOWA\left(A_{\delta \left(1\right)},A_{\delta \left(2\right)},\ldots ,A_{\delta \left(n\right)}\right)\leq q-SFREOWA\left(A_{\delta \left(1\right)}^{*},A_{\delta \left(2\right)}^{*},\ldots ,A_{\delta \left(n\right)}^{*}\right)$.

**Proof:** The proof follows the same steps as in Theorem 5.

### 3.5 Some specific cases regarding q−SFREOWAoperator

From Theorem 6, we have

$$q-SFREOWA\left(A_{\delta \left(1\right)},A_{\delta \left(2\right)},\ldots ,A_{\delta \left(n\right)}\right)=\left[\left\langle \begin{array}{c} \sqrt[{q}]{\frac{\prod_{i=1}^{n}\;{\left(1+{\underline{\zeta }}_{\delta \left(i\right)}^{q}\right)}^{\omega_{i}}-\prod_{i=1}^{n}\;{\left(1-{\underline{\zeta }}_{\delta \left(i\right)}^{q}\right)}^{\omega_{i}}}{\prod_{i=1}^{n}\;{\left(1+{\underline{\zeta }}_{\delta \left(i\right)}^{q}\right)}^{\omega_{i}}+{\prod_{i=1}^{n}\;\left(1-{\underline{\zeta }}_{\delta \left(i\right)}^{q}\right)}^{\omega_{i}}}},\sqrt[{q}]{\frac{2{\prod_{i=1}^{n}\;\left({\underline{\eta }}_{\delta \left(i\right)}^{q}\right)}^{\omega_{i}}}{{\prod_{i=1}^{n}\;\left(2-{\underline{\eta }}_{\delta \left(i\right)}^{q}\right)}^{\omega_{i}}+\prod_{i=1}^{n}\;{\left({\underline{\eta }}_{\delta \left(i\right)}^{q}\right)}^{\omega_{i}}}},\sqrt[{q}]{\frac{2{\prod_{i=1}^{n}\;\left(\;{\underline{\xi }}_{\delta \left(i\right)}^{q}\right)}^{\omega_{i}}}{{\prod_{i=1}^{n}\;\left(2-\;{\underline{\xi }}_{\delta \left(i\right)}^{q}\right)}^{\omega_{i}}+\prod_{i=1}^{n}\;{\left(\;{\underline{\xi }}_{\delta \left(i\right)}^{q}\right)}^{\omega_{i}}},}\\ \sqrt[{q}]{\frac{\prod_{i=1}^{n}\;{\left(1+{\bar{\zeta }}_{\delta \left(i\right)}^{q}\right)}^{\omega_{i}}-\prod_{i=1}^{n}\;{\left(1-{\bar{\zeta }}_{\delta \left(i\right)}^{q}\right)}^{\omega_{i}}}{\prod_{i=1}^{n}\;{\left(1+{\bar{\zeta }}_{\delta \left(i\right)}^{q}\right)}^{\omega_{i}}+{\prod_{i=1}^{n}\;\left(1-{\bar{\zeta }}_{\delta \left(i\right)}^{q}\right)}^{\omega_{i}}}},\sqrt[{q}]{\frac{2{\prod_{i=1}^{n}\;\left({\bar{\eta }}_{\delta \left(i\right)}^{q}\right)}^{\omega_{i}}}{{\prod_{i=1}^{n}\;\left(2-{\bar{\eta }}_{\delta \left(i\right)}^{q}\right)}^{\omega_{i}}+\prod_{i=1}^{n}\;{\left({\bar{\eta }}_{\delta \left(i\right)}^{q}\right)}^{\omega_{i}}}},\sqrt[{q}]{\frac{2{\prod_{i=1}^{n}\;\left(\;{\bar{\xi }}_{\delta \left(i\right)}^{q}\right)}^{\omega_{i}}}{{\prod_{i=1}^{n}\;\left(2-\;{\bar{\xi }}_{\delta \left(i\right)}^{q}\right)}^{\omega_{i}}+\prod_{i=1}^{n}\;{\left(\;{\bar{\xi }}_{\delta \left(i\right)}^{q}\right)}^{\omega_{i}}}} \end{array}\right\rangle \right]$$

We are facing the following cases.

Case 1 If ${\underline{\zeta }}_{\delta \left(i\right)}={\underline{\eta }}_{\delta \left(i\right)}={\underline{\xi }}_{\delta \left(i\right)}=0\;and\;q=2$ then

$$q-SFREOWA\left(A_{\delta \left(1\right)},A_{\delta \left(2\right)},\ldots ,A_{\delta \left(n\right)}\right)={{⊕}_{Ḛ}}_{i=1}^{n}\left(\omega_{i}A_{\delta \left(i\right)}\right)$$

$$=\left[\left\langle \sqrt{\frac{\prod_{i=1}^{n}\;{\left(1+{\bar{\zeta }}_{\delta \left(i\right)}^{2}\right)}^{\omega_{i}}-\prod_{i=1}^{n}\;{\left(1-{\bar{\zeta }}_{\delta \left(i\right)}^{2}\right)}^{\omega_{i}}}{\prod_{i=1}^{n}\;{\left(1+{\bar{\zeta }}_{\delta \left(i\right)}^{2}\right)}^{\omega_{i}}+{\prod_{i=1}^{n}\;\left(1-{\bar{\zeta }}_{\delta \left(i\right)}^{2}\right)}^{\omega_{i}}}},\sqrt{\frac{2{\prod_{i=1}^{n}\;\left({\bar{\eta }}_{\delta \left(i\right)}^{2}\right)}^{\omega_{i}}}{{\prod_{i=1}^{n}\;\left(2-{\bar{\eta }}_{\delta \left(i\right)}^{2}\right)}^{\omega_{i}}+\prod_{i=1}^{n}\;{\left({\bar{\eta }}_{\delta \left(i\right)}^{2}\right)}^{\omega_{i}}}},\sqrt{\frac{2{\prod_{i=1}^{n}\;\left(\;{\bar{\xi }}_{\delta \left(i\right)}^{2}\right)}^{\omega_{i}}}{{\prod_{i=1}^{n}\;\left(2-\;{\bar{\xi }}_{\delta \left(i\right)}^{2}\right)}^{\omega_{i}}+\prod_{i=1}^{n}\;{\left(\;{\bar{\xi }}_{\delta \left(i\right)}^{2}\right)}^{\omega_{i}}}}\right\rangle \right]$$

$$=SFREOWA\left(A_{\delta \left(1\right)},A_{\delta \left(2\right)},\ldots ,A_{\delta \left(n\right)}\right)={{⊕}_{Ḛ}}_{i=1}^{n}\left(\omega_{i}A_{\delta \left(i\right)}\right)$$

(The spherical fuzzy rough Einstein ordered weighted averaging operator).

Case 2 If ${\underline{\xi }}_{\delta \left(i\right)}={\bar{\xi }}_{\delta \left(i\right)}=0\;and\;q=2$ then

$$q-SFREOWA\left(A_{\delta \left(1\right)},A_{\delta \left(2\right)},\ldots ,A_{\delta \left(n\right)}\right)={{⊕}_{Ḛ}}_{i=1}^{n}\left(\omega_{i}A_{\delta \left(i\right)}\right)=\left[\left\langle \begin{array}{c} \sqrt{\frac{\prod_{i=1}^{n}\;{\left(1+{\underline{\zeta }}_{\delta \left(i\right)}^{2}\right)}^{\omega_{i}}-\prod_{i=1}^{n}\;{\left(1-{\underline{\zeta }}_{\delta \left(i\right)}^{2}\right)}^{\omega_{i}}}{\prod_{i=1}^{n}\;{\left(1+{\underline{\zeta }}_{\delta \left(i\right)}^{2}\right)}^{\omega_{i}}+{\prod_{i=1}^{n}\;\left(1-{\underline{\zeta }}_{\delta \left(i\right)}^{2}\right)}^{\omega_{i}}}},\sqrt{\frac{2{\prod_{i=1}^{n}\;\left({\underline{\eta }}_{\delta \left(i\right)}^{2}\right)}^{\omega_{i}}}{{\prod_{i=1}^{n}\;\left(2-{\underline{\eta }}_{\delta \left(i\right)}^{2}\right)}^{\omega_{i}}+\prod_{i=1}^{n}\;{\left({\underline{\eta }}_{\delta \left(i\right)}^{2}\right)}^{\omega_{i}}},}\\ \sqrt{\frac{\prod_{i=1}^{n}\;{\left(1+{\bar{\zeta }}_{\delta \left(i\right)}^{2}\right)}^{\omega_{i}}-\prod_{i=1}^{n}\;{\left(1-{\bar{\zeta }}_{\delta \left(i\right)}^{2}\right)}^{\omega_{i}}}{\prod_{i=1}^{n}\;{\left(1+{\bar{\zeta }}_{\delta \left(i\right)}^{2}\right)}^{\omega_{i}}+{\prod_{i=1}^{n}\;\left(1-{\bar{\zeta }}_{\delta \left(i\right)}^{2}\right)}^{\omega_{i}}}},\sqrt{\frac{2{\prod_{i=1}^{n}\;\left({\bar{\eta }}_{\delta \left(i\right)}^{2}\right)}^{\omega_{i}}}{{\prod_{i=1}^{n}\;\left(2-{\bar{\eta }}_{\delta \left(i\right)}^{2}\right)}^{\omega_{i}}+\prod_{i=1}^{n}\;{\left({\bar{\eta }}_{\delta \left(i\right)}^{2}\right)}^{\omega_{i}}}} \end{array}\right\rangle \right]$$

$$=PyFREOWA\left(A_{1},A_{2},\ldots ,A_{n}\right)={{⊕}_{Ḛ}}_{i=1}^{n}\left(\omega_{i}A_{i}\right)$$

(The Pythagorean fuzzy rough Einstein ordered weighted averaging operator).

Case 3 If q=1 then

$$q-SFREOWA\left(A_{\delta \left(1\right)},A_{\delta \left(2\right)},\ldots ,A_{\delta \left(n\right)}\right)=\left[\left\langle \begin{array}{c} \frac{\prod_{i=1}^{n}\;{\left(1+{\underline{\zeta }}_{\delta \left(i\right)}\right)}^{\omega_{i}}-\prod_{i=1}^{n}\;{\left(1-{\underline{\zeta }}_{\delta \left(i\right)}\right)}^{\omega_{i}}}{\prod_{i=1}^{n}\;{\left(1+{\underline{\zeta }}_{\delta \left(i\right)}\right)}^{\omega_{i}}+{\prod_{i=1}^{n}\;\left(1-{\underline{\zeta }}_{\delta \left(i\right)}\right)}^{\omega_{i}}},\frac{2{\prod_{i=1}^{n}\;\left({\underline{\eta }}_{\delta \left(i\right)}\right)}^{\omega_{i}}}{{\prod_{i=1}^{n}\;\left(2-{\underline{\eta }}_{\delta \left(i\right)}\right)}^{\omega_{i}}+\prod_{i=1}^{n}\;{\left({\underline{\eta }}_{\delta \left(i\right)}\right)}^{\omega_{i}}},\frac{2{\prod_{i=1}^{n}\;\left(\;{\underline{\xi }}_{\delta \left(i\right)}\right)}^{\omega_{i}}}{{\prod_{i=1}^{n}\;\left(2-{\underline{\xi }}_{\delta \left(i\right)}\right)}^{\omega_{i}}+\prod_{i=1}^{n}\;{\left({\underline{\xi }}_{\delta \left(i\right)}\right)}^{\omega_{i}}},\\ \frac{\prod_{i=1}^{n}\;{\left(1+{\bar{\zeta }}_{\delta \left(i\right)}\right)}^{\omega_{i}}-\prod_{i=1}^{n}\;{\left(1-{\bar{\zeta }}_{\delta \left(i\right)}\right)}^{\omega_{i}}}{\prod_{i=1}^{n}\;{\left(1+{\bar{\zeta }}_{\delta \left(i\right)}\right)}^{\omega_{i}}+{\prod_{i=1}^{n}\;\left(1-{\bar{\zeta }}_{\delta \left(i\right)}\right)}^{\omega_{i}}},\frac{2{\prod_{i=1}^{n}\;\left({\bar{\eta }}_{\delta \left(i\right)}\right)}^{\omega_{i}}}{{\prod_{i=1}^{n}\;\left(2-{\bar{\eta }}_{\delta \left(i\right)}\right)}^{\omega_{i}}+\prod_{i=1}^{n}\;{\left({\bar{\eta }}_{\delta \left(i\right)}\right)}^{\omega_{i}}},\frac{2{\prod_{i=1}^{n}\;\left(\;{\bar{\xi }}_{\delta \left(i\right)}\right)}^{\omega_{i}}}{{\prod_{i=1}^{n}\;\left(2-\;{\bar{\xi }}_{\delta \left(i\right)}\right)}^{\omega_{i}}+\prod_{i=1}^{n}\;{\left({\bar{\xi }}_{\delta \left(i\right)}\right)}^{\omega_{i}}} \end{array}\right\rangle \right]$$

$$=PFREOWA\left(A_{\delta \left(1\right)},A_{\delta \left(2\right)},\ldots ,A_{\delta \left(n\right)}\right)={{⊕}_{Ḛ}}_{i=1}^{n}\left(\omega_{i}A_{\delta \left(i\right)}\right)$$

(The picture fuzzy rough Einstein ordered weighted averaging operator).

Case 4 If ${\underline{\xi }}_{i}={\bar{\xi }}_{i}=0\;and\;q=1$ then

$$q-SFREOWA\left(A_{\delta \left(1\right)},A_{\delta \left(2\right)},\ldots ,A_{\delta \left(n\right)}\right)={{⊕}_{Ḛ}}_{i=1}^{n}\left(\omega_{i}A_{i}\right)\left[\left\langle \begin{array}{c} \frac{\prod_{i=1}^{n}\;{\left(1+{\underline{\zeta }}_{\delta \left(i\right)}\right)}^{\omega_{i}}-\prod_{i=1}^{n}\;{\left(1-{\underline{\zeta }}_{\delta \left(i\right)}\right)}^{\omega_{i}}}{\prod_{i=1}^{n}\;{\left(1+{\underline{\zeta }}_{\delta \left(i\right)}\right)}^{\omega_{i}}+{\prod_{i=1}^{n}\;\left(1-{\underline{\zeta }}_{\delta \left(i\right)}\right)}^{\omega_{i}}},\frac{2{\prod_{i=1}^{n}\;\left({\underline{\eta }}_{\delta \left(i\right)}\right)}^{\omega_{i}}}{{\prod_{i=1}^{n}\;\left(2-{\underline{\eta }}_{\delta \left(i\right)}\right)}^{\omega_{i}}+\prod_{i=1}^{n}\;{\left({\underline{\eta }}_{\delta \left(i\right)}\right)}^{\omega_{i}}},\\ \frac{\prod_{i=1}^{n}\;{\left(1+{\bar{\zeta }}_{\delta \left(i\right)}\right)}^{\omega_{i}}-\prod_{i=1}^{n}\;{\left(1-{\bar{\zeta }}_{\delta \left(i\right)}\right)}^{\omega_{i}}}{\prod_{i=1}^{n}\;{\left(1+{\bar{\zeta }}_{\delta \left(i\right)}\right)}^{\omega_{i}}+{\prod_{i=1}^{n}\;\left(1-{\bar{\zeta }}_{\delta \left(i\right)}\right)}^{\omega_{i}}},\frac{2{\prod_{i=1}^{n}\;\left({\bar{\eta }}_{\delta \left(i\right)}\right)}^{\omega_{i}}}{{\prod_{i=1}^{n}\;\left(2-{\bar{\eta }}_{\delta \left(i\right)}\right)}^{\omega_{i}}+\prod_{i=1}^{n}\;{\left({\bar{\eta }}_{\delta \left(i\right)}\right)}^{\omega_{i}}} \end{array}\right\rangle \right]=IFREOWA\left(A_{\delta \left(1\right)},A_{\delta \left(2\right)},\ldots ,A_{\delta \left(n\right)}\right)={{⊕}_{Ḛ}}_{i=1}^{n}\left(\omega_{i}A_{\delta \left(i\right)}\right)$$

(The intuitionistic fuzzy rough Einstein ordered weighted averaging operator).

**Fig. 5:**
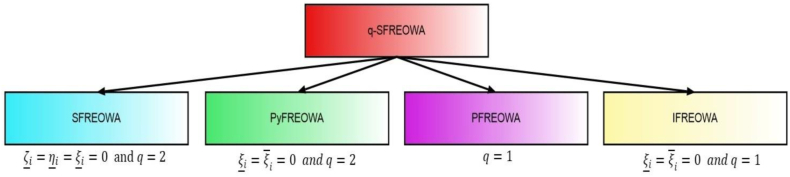
Specificcasesregardingtheq−SFREOWAoperator.

Fig. 5 represents specificcasesregardingthe q−SFREOWAoperator.

Example 3 Consider four q-SFRNs $A_{1}=(0.4,0.1,0.3,0.2,0.4,0.3$), $A_{2}=\left(0.5,0.9,0.5,0.8,0.6,0.2\right)\text{,}$ $A_{3}=\left(0.5,0.9,0.4,0.2,0.6,0.3\right)\text{,}$ and $A_{4}=\left(0.2,0.5,0.5,0.8,0.9,0.3\right)\text{.}$ If ω= ${\left(0.3,0.1,0.4,0.2\right)}^{T}$ and q=3, then the q-SFREOWA operator, as defined in Definition (15), can be computed as:

Now we calculate the score values of $A_{i}\left(i=1,2,3,4\right)$.

$$Sco\left(A_{1}\right)=\frac{{2+\left(0.4\right)}^{3}+{\left(0.2\right)}^{3}-{\left(0.1\right)}^{3}-{\left(0.4\right)}^{3}-{\left(0.3\right)}^{3}-{\left(0.3\right)}^{3}}{3}=\left(0.6510\right)$$

$$Sco\left(A_{2}\right)=\frac{{2+\left(0.5\right)}^{3}+{\left(0.8\right)}^{3}-{\left(0.9\right)}^{3}-{\left(0.6\right)}^{3}-{\left(0.5\right)}^{3}-{\left(0.2\right)}^{3}}{3}=\left(0.5197\right)$$

$$Sco\left(A_{3}\right)=\frac{{2+\left(0.5\right)}^{3}+{\left(0.2\right)}^{3}-{\left(0.9\right)}^{3}-{\left(0.6\right)}^{3}-{\left(0.4\right)}^{3}-{\left(0.3\right)}^{3}}{3}=\left(0.3657\right)$$

$$Sco\left(A_{4}\right)=\frac{{2+\left(0.2\right)}^{3}+{\left(0.8\right)}^{3}-{\left(0.5\right)}^{3}-{\left(0.9\right)}^{3}-{\left(0.5\right)}^{3}-{\left(0.3\right)}^{3}}{3}=\left(0.5047\right)$$

$$\text{Since}\;Sco\left(A_{1}\right)>\;Sco\left(A_{2}\right)>\;Sco\left(A_{4}\right)>\;Sco\left(A_{3}\right)$$

$$\left(A_{1}\right)>\;\left(A_{2}\right)>\;\left(A_{4}\right)>\left(A_{3}\right)$$

Hence

$$A_{\delta \left(1\right)}=A_{1}^{\circ }=\left(0.4,0.1,0.3,0.2,0.4,0.3\right)$$

$$A_{\delta \left(2\right)}=A_{2}^{\circ }=\left(0.5,0.9,0.5,0.8,0.6,0.2\right)$$

$$A_{\delta \left(3\right)}=A_{3}^{\circ }=\left(0.2,0.5,0.5,0.8,0.9,0.3\right)$$

$$A_{\delta \left(4\right)}=A_{4}^{\circ }=\left(0.5,0.9,0.4,0.2,0.6,0.3\right)$$

For $A_{\delta \left(i\right)}\left(i=1,2,3,4\right)$ we have

$$q-SFREOWA\left(\;A_{\delta \left(1\right)},A_{\delta \left(2\right)},A_{\delta \left(3\right)},A_{\delta \left(4\right)}\right)=$$

then the q-SFREWA operator defined in Definition (14) can be calculated as:

Hence

$$\left[\left\langle \begin{array}{c} \sqrt[{q}]{\frac{\prod_{i=1}^{4}\;{\left(1+{\underline{\zeta }}_{\delta \left(i\right)}^{q}\right)}^{\omega_{i}}-\prod_{i=1}^{4}\;{\left(1-{\underline{\zeta }}_{\delta \left(i\right)}^{q}\right)}^{\omega_{i}}}{\prod_{i=1}^{4}\;{\left(1+{\underline{\zeta }}_{\delta \left(i\right)}^{q}\right)}^{\omega_{i}}+{\prod_{i=1}^{4}\;\left(1-{\underline{\zeta }}_{\delta \left(i\right)}^{q}\right)}^{\omega_{i}}}},\sqrt[{q}]{\frac{2{\prod_{i=1}^{4}\;\left({\underline{\eta }}_{\delta \left(i\right)}^{q}\right)}^{\omega_{i}}}{{\prod_{i=1}^{4}\;\left(2-{\underline{\eta }}_{\delta \left(i\right)}^{q}\right)}^{\omega_{i}}+\prod_{i=1}^{4}\;{\left({\underline{\eta }}_{\delta \left(i\right)}^{q}\right)}^{\omega_{i}}}},\sqrt[{q}]{\frac{2{\prod_{i=1}^{4}\;\left(\;{\underline{\xi }}_{\delta \left(i\right)}^{q}\right)}^{\omega_{i}}}{{\prod_{i=1}^{4}\;\left(2-\;{\underline{\xi }}_{\delta \left(i\right)}^{q}\right)}^{\omega_{i}}+\prod_{i=1}^{4}\;{\left(\;{\underline{\xi }}_{\delta \left(i\right)}^{q}\right)}^{\omega_{i}}},}\\ \sqrt[{q}]{\frac{\prod_{i=1}^{4}\;{\left(1+{\bar{\zeta }}_{\delta \left(i\right)}^{q}\right)}^{\omega_{i}}-\prod_{i=1}^{4}\;{\left(1-{\bar{\zeta }}_{\delta \left(i\right)}^{q}\right)}^{\omega_{i}}}{\prod_{i=1}^{4}\;{\left(1+{\bar{\zeta }}_{\delta \left(i\right)}^{q}\right)}^{\omega_{i}}+{\prod_{i=1}^{4}\;\left(1-{\bar{\zeta }}_{\delta \left(i\right)}^{q}\right)}^{\omega_{i}}}},\sqrt[{q}]{\frac{2{\prod_{i=1}^{4}\;\left({\bar{\eta }}_{\delta \left(i\right)}^{q}\right)}^{\omega_{i}}}{{\prod_{i=1}^{4}\;\left(2-{\bar{\eta }}_{\delta \left(i\right)}^{q}\right)}^{\omega_{i}}+\prod_{i=1}^{4}\;{\left({\bar{\eta }}_{\delta \left(i\right)}^{q}\right)}^{\omega_{i}}}},\sqrt[{q}]{\frac{2{\prod_{i=1}^{4}\;\left(\;{\bar{\xi }}_{\delta \left(i\right)}^{q}\right)}^{\omega_{i}}}{{\prod_{i=1}^{4}\;\left(2-\;{\bar{\xi }}_{\delta \left(i\right)}^{q}\right)}^{\omega_{i}}+\prod_{i=1}^{4}\;{\left(\;{\bar{\xi }}_{\delta \left(i\right)}^{q}\right)}^{\omega_{i}}}} \end{array}\right\rangle \right]=\left(0.3916,0.6422,0.6385,0.6535,0.7535,0.5366\right)$$

### 3.6 q−SFREHWAoperator

Definition 16 Assuming $A_{i}=\left({\underline{\zeta }}_{i},{\underline{\eta }}_{i},{\underline{\xi }}_{i},{\bar{\zeta }}_{i},{\bar{\eta }}_{i},{\bar{\xi }}_{i}\right)\left(i=1,2,\ldots ,n\right)$ be a family of q-SFRNs, the q-spherical fuzzy rough Einstein hybrid averaging operator (q-SFREHWA) is defined as a mapping $q-SFREHWA:A^{n}⟶A$. This mapping is connected with a weight vector ${\left(\omega_{1},\omega_{2},\ldots ,\omega_{n}\right)}^{T}\text{,}$ which complies with the constraints $\omega_{i}>0$ and the constrain $\sum_{i=1}^{n}\omega_{i}=1$.

$$q-SFREHWA\left(A_{\alpha \left(1\right)},A_{\alpha \left(2\right)},\ldots ,A_{\alpha \left(n\right)}\right)=A_{\alpha \left(1\right)}{⊕}_{Ḛ}A_{\alpha \left(2\right)},\ldots ,{⊕}_{Ḛ}A_{\alpha \left(n\right)}={{⊕}_{Ḛ}}_{i=1}^{n}\left(\omega_{i}A_{\alpha \left(i\right)}\right)$$

Where α(1),α(2),…,α(n) is a permutation of (1,2,3,‥,n) such that $A_{\alpha \left(1\right)}\leq A_{\alpha \left(i-1\right)}$ for all i=1,2,3,‥,n.

Now,

Given $A_{i}=\left({\underline{\zeta }}_{i},{\underline{\eta }}_{i},{\underline{\xi }}_{i},{\bar{\zeta }}_{i},{\bar{\eta }}_{i},{\bar{\xi }}_{i}\right)$ for (i=1,2,…,n) as a collection of q-SFRNs, and ${\omega =\left(\omega_{1},\omega_{2},\ldots ,\omega_{n}\right)}^{T}$ is the weight vector satisfying $\omega_{i}>0$ and $\sum_{i=1}^{n}\omega_{i}=1\text{.}$ Then

$$q-SFREHWA\left(A_{\alpha \left(1\right)},A_{\alpha \left(2\right)},\ldots ,A_{\alpha \left(n\right)}\right)=\left[\left\langle \begin{array}{c} \sqrt[{q}]{\frac{\prod_{i=1}^{n}\;{\left(1+{\underline{\zeta }}_{\alpha \left(i\right)}^{q}\right)}^{\omega_{i}}-\prod_{i=1}^{n}\;{\left(1-{\underline{\zeta }}_{\alpha \left(i\right)}^{q}\right)}^{\omega_{i}}}{\prod_{i=1}^{n}\;{\left(1+{\underline{\zeta }}_{\alpha \left(i\right)}^{q}\right)}^{\omega_{i}}+{\prod_{i=1}^{n}\;\left(1-{\underline{\zeta }}_{\alpha \left(i\right)}^{q}\right)}^{\omega_{i}}}},\sqrt[{q}]{\frac{2{\prod_{i=1}^{n}\;\left({\underline{\eta }}_{\alpha \left(i\right)}^{q}\right)}^{\omega_{i}}}{{\prod_{i=1}^{n}\;\left(2-{\underline{\eta }}_{\alpha \left(i\right)}^{q}\right)}^{\omega_{i}}+\prod_{i=1}^{n}\;{\left({\underline{\eta }}_{\alpha \left(i\right)}^{q}\right)}^{\omega_{i}}}},\sqrt[{q}]{\frac{2{\prod_{i=1}^{n}\;\left(\;{\underline{\xi }}_{\alpha \left(i\right)}^{q}\right)}^{\omega_{i}}}{{\prod_{i=1}^{n}\;\left(2-\;{\underline{\xi }}_{\alpha \left(i\right)}^{q}\right)}^{\omega_{i}}+\prod_{i=1}^{n}\;{\left(\;{\underline{\xi }}_{\alpha \left(i\right)}^{q}\right)}^{\omega_{i}}},}\\ \sqrt[{q}]{\frac{\prod_{i=1}^{n}\;{\left(1+{\bar{\zeta }}_{\alpha \left(i\right)}^{q}\right)}^{\omega_{i}}-\prod_{i=1}^{n}\;{\left(1-{\bar{\zeta }}_{\alpha \left(i\right)}^{q}\right)}^{\omega_{i}}}{\prod_{i=1}^{n}\;{\left(1+{\bar{\zeta }}_{\alpha \left(i\right)}^{q}\right)}^{\omega_{i}}+{\prod_{i=1}^{n}\;\left(1-{\bar{\zeta }}_{\alpha \left(i\right)}^{q}\right)}^{\omega_{i}}}},\sqrt[{q}]{\frac{2{\prod_{i=1}^{n}\;\left({\bar{\eta }}_{\alpha \left(i\right)}^{q}\right)}^{\omega_{i}}}{{\prod_{i=1}^{n}\;\left(2-{\bar{\eta }}_{\alpha \left(i\right)}^{q}\right)}^{\omega_{i}}+\prod_{i=1}^{n}\;{\left({\bar{\eta }}_{\alpha \left(i\right)}^{q}\right)}^{\omega_{i}}}},\sqrt[{q}]{\frac{2{\prod_{i=1}^{n}\;\left(\;{\bar{\xi }}_{\alpha \left(i\right)}^{q}\right)}^{\omega_{i}}}{{\prod_{i=1}^{n}\;\left(2-\;{\bar{\xi }}_{\alpha \left(i\right)}^{q}\right)}^{\omega_{i}}+\prod_{i=1}^{n}\;{\left(\;{\bar{\xi }}_{\alpha \left(i\right)}^{q}\right)}^{\omega_{i}}}} \end{array}\right\rangle \right]$$

Where α(i), the $i^{th}$ greatest value as determined by the overall order of $A_{\alpha \left(1\right)}\geq A_{\alpha \left(2\right)}\geq \ldots \geq A_{\alpha \left(n\right)}$ where $A_{\alpha \left(i\right)}$ has the $i^{th}$ highest weighted value.

Theorem 10 *Let*$A_{i}=\left({\underline{\zeta }}_{i},{\underline{\eta }}_{i},{\underline{\xi }}_{i},{\bar{\zeta }}_{i},{\bar{\eta }}_{i},{\bar{\xi }}_{i}\right)$*for*(i=1,2,…,n)*be a collection of q-SFRNs*, *and*${\omega =\left(\omega_{1},\omega_{2},\ldots ,\omega_{n}\right)}^{T}$*be the weight vector satisfying*$\omega_{i}>0$*and*$\sum_{i=1}^{n}\omega_{i}=1\text{.}$*If these conditions are met*, *it is referred to as the q-SFREHWA operator*.

$$q-SFREHWA\left(A_{\alpha \left(1\right)},A_{\alpha \left(2\right)},\ldots ,A_{\alpha \left(n\right)}\right)=\left[\left\langle \begin{array}{c} \sqrt[{q}]{\frac{\prod_{i=1}^{n}\;{\left(1+{\underline{\zeta }}_{\alpha \left(i\right)}^{q}\right)}^{\omega_{i}}-\prod_{i=1}^{n}\;{\left(1-{\underline{\zeta }}_{\alpha \left(i\right)}^{q}\right)}^{\omega_{i}}}{\prod_{i=1}^{n}\;{\left(1+{\underline{\zeta }}_{\alpha \left(i\right)}^{q}\right)}^{\omega_{i}}+{\prod_{i=1}^{n}\;\left(1-{\underline{\zeta }}_{\alpha \left(i\right)}^{q}\right)}^{\omega_{i}}}},\sqrt[{q}]{\frac{2{\prod_{i=1}^{n}\;\left({\underline{\eta }}_{\alpha \left(i\right)}^{q}\right)}^{\omega_{i}}}{{\prod_{i=1}^{n}\;\left(2-{\underline{\eta }}_{\alpha \left(i\right)}^{q}\right)}^{\omega_{i}}+\prod_{i=1}^{n}\;{\left({\underline{\eta }}_{\alpha \left(i\right)}^{q}\right)}^{\omega_{i}}}},\sqrt[{q}]{\frac{2{\prod_{i=1}^{n}\;\left(\;{\underline{\xi }}_{\alpha \left(i\right)}^{q}\right)}^{\omega_{i}}}{{\prod_{i=1}^{n}\;\left(2-\;{\underline{\xi }}_{\alpha \left(i\right)}^{q}\right)}^{\omega_{i}}+\prod_{i=1}^{n}\;{\left(\;{\underline{\xi }}_{\alpha \left(i\right)}^{q}\right)}^{\omega_{i}}},}\\ \sqrt[{q}]{\frac{\prod_{i=1}^{n}\;{\left(1+{\bar{\zeta }}_{\alpha \left(i\right)}^{q}\right)}^{\omega_{i}}-\prod_{i=1}^{n}\;{\left(1-{\bar{\zeta }}_{\alpha \left(i\right)}^{q}\right)}^{\omega_{i}}}{\prod_{i=1}^{n}\;{\left(1+{\bar{\zeta }}_{\alpha \left(i\right)}^{q}\right)}^{\omega_{i}}+{\prod_{i=1}^{n}\;\left(1-{\bar{\zeta }}_{\alpha \left(i\right)}^{q}\right)}^{\omega_{i}}}},\sqrt[{q}]{\frac{2{\prod_{i=1}^{n}\;\left({\bar{\eta }}_{\alpha \left(i\right)}^{q}\right)}^{\omega_{i}}}{{\prod_{i=1}^{n}\;\left(2-{\bar{\eta }}_{\alpha \left(i\right)}^{q}\right)}^{\omega_{i}}+\prod_{i=1}^{n}\;{\left({\bar{\eta }}_{\alpha \left(i\right)}^{q}\right)}^{\omega_{i}}}},\sqrt[{q}]{\frac{2{\prod_{i=1}^{n}\;\left(\;{\bar{\xi }}_{\alpha \left(i\right)}^{q}\right)}^{\omega_{i}}}{{\prod_{i=1}^{n}\;\left(2-\;{\bar{\xi }}_{\alpha \left(i\right)}^{q}\right)}^{\omega_{i}}+\prod_{i=1}^{n}\;{\left(\;{\bar{\xi }}_{\alpha \left(i\right)}^{q}\right)}^{\omega_{i}}}} \end{array}\right\rangle \right]$$

**Proof:** The proof follows the same steps as in Theorem 2.

Theorem 11 (Idempotency) *Assuming*$A_{i}=\left({\underline{\zeta }}_{i},{\underline{\eta }}_{i},{\underline{\xi }}_{i},{\bar{\zeta }}_{i},{\bar{\eta }}_{i},{\bar{\xi }}_{i}\right)\left(i=1,2,\ldots ,n\right)$*be a collection of*$q-SFRNs\;\text{and}\;{\left(\omega_{1},\omega_{2},\ldots ,\omega_{n}\right)}^{T}$*signifies the weight vector adhering to the condition*$\omega_{i}>0$*and the constrain*$\sum_{i=1}^{n}\omega_{i}=1\text{.}$$A_{i}\left(i=1,2,\ldots ,n\right)$*are the same*∀i,*then*

$$q-SFREHWA\left(A_{\alpha \left(1\right)},A_{\alpha \left(2\right)},\ldots ,A_{\alpha \left(n\right)}\right)=A$$

**Proof:** The proof follows the same steps as in Theorem 3.

Theorem 12 (Boundness) *Assuming*$A_{i}=\left({\underline{\zeta }}_{i},{\underline{\eta }}_{i},{\underline{\xi }}_{i},{\bar{\zeta }}_{i},{\bar{\eta }}_{i},{\bar{\xi }}_{i}\right)\left(i=1,2,\ldots ,n\right)$*be a collection of*$q-SFRNs\;\text{and}\;{\left(\omega_{1},\omega_{2},\ldots ,\omega_{n}\right)}^{T}$*signifies the weight vector adhering to the condition*$\omega_{i}>0$*and the constrain*$\sum_{i=1}^{n}\omega_{i}=1$.

$$\text{Let}\;A^{-}=\left(\min\;{\underline{\zeta }}_{i},\max\;{\underline{\eta }}_{i},\max\;{\underline{\xi }}_{i},\min\;{\bar{\zeta }}_{i},\max\;{\bar{\eta }}_{i},\max\;{\bar{\xi }}_{i}\;\right)\;\text{and}\;A^{+}=\left(\max\;{\underline{\zeta }}_{i},\min\;{\underline{\eta }}_{i},\min\;{\underline{\xi }}_{i},\max\;{\bar{\zeta }}_{i},\min\;{\bar{\eta }}_{i},\min\;{\bar{\xi }}_{i}\right)$$

$$\text{Then}\;A^{-}\leq q-SFREHWA\left(A_{\alpha \left(1\right)},A_{\alpha \left(2\right)},\ldots ,A_{\alpha \left(n\right)}\right)\leq A^{+}$$

**Proof:** The proof follows the same steps as in Theorem 4.

Theorem 13 (Monotonicity) *Assuming*$A_{i}=\left({\underline{\zeta }}_{i},{\underline{\eta }}_{i},{\underline{\xi }}_{i},{\bar{\zeta }}_{i},{\bar{\eta }}_{i},{\bar{\xi }}_{i}\right)\left(i=1,2,\ldots ,n\right)\;\text{and}\;{A_{i}}^{*}=\left({{\underline{\zeta }}_{i}}^{*},{{\underline{\eta }}_{i}}^{*},{{\underline{\xi }}_{i}}^{*},{{\bar{\zeta }}_{i}}^{*},{{\bar{\eta }}_{i}}^{*},{{\bar{\xi }}_{i}}^{*}\right)\left(i=1,2,\ldots ,n\right)$*be a collection of two*q−SFRNs.*If*${\underline{\zeta }}_{i}\leq {{\underline{\zeta }}_{i}}^{*},{\underline{\eta }}_{i}\leq {{\underline{\eta }}_{i}}^{*}\text{,}$${\underline{\xi }}_{i}\leq {{\underline{\xi }}_{i}}^{*}$and${\bar{\zeta }}_{i}\leq {{\bar{\zeta }}_{i}}^{*},{\bar{\eta }}_{i}\leq {{\bar{\eta }}_{i}}^{*}\text{,}$${\bar{\xi }}_{i}\leq {{\bar{\xi }}_{i}}^{*}$for all i,*then*

$$q-SFREHWA\left(A_{\alpha \left(1\right)},A_{\alpha \left(2\right)},\ldots ,A_{\alpha \left(n\right)}\right)\leq q--SFREHWA\left(A_{\alpha \left(1\right)}^{*},A_{\alpha \left(2\right)}^{*},\ldots ,A_{\alpha \left(n\right)}^{*}\right)\text{.}$$

**Proof:** The proof follows the same steps as in Theorem 5.

### 3.7 Some specific cases regarding the q−SFREHWAoperator

From Theorem 10, we have

$$q-SFREHWA\left(A_{\alpha \left(1\right)},A_{\alpha \left(2\right)},\ldots ,A_{\alpha \left(n\right)}\right)=\left[\left\langle \begin{array}{c} \sqrt[{q}]{\frac{\prod_{i=1}^{n}\;{\left(1+{\underline{\zeta }}_{\alpha \left(i\right)}^{q}\right)}^{\omega_{i}}-\prod_{i=1}^{n}\;{\left(1-{\underline{\zeta }}_{\alpha \left(i\right)}^{q}\right)}^{\omega_{i}}}{\prod_{i=1}^{n}\;{\left(1+{\underline{\zeta }}_{\alpha \left(i\right)}^{q}\right)}^{\omega_{i}}+{\prod_{i=1}^{n}\;\left(1-{\underline{\zeta }}_{\alpha \left(i\right)}^{q}\right)}^{\omega_{i}}}},\sqrt[{q}]{\frac{2{\prod_{i=1}^{n}\;\left({\underline{\eta }}_{\alpha \left(i\right)}^{q}\right)}^{\omega_{i}}}{{\prod_{i=1}^{n}\;\left(2-{\underline{\eta }}_{\alpha \left(i\right)}^{q}\right)}^{\omega_{i}}+\prod_{i=1}^{n}\;{\left({\underline{\eta }}_{\alpha \left(i\right)}^{q}\right)}^{\omega_{i}}}},\sqrt[{q}]{\frac{2{\prod_{i=1}^{n}\;\left(\;{\underline{\xi }}_{\alpha \left(i\right)}^{q}\right)}^{\omega_{i}}}{{\prod_{i=1}^{n}\;\left(2-\;{\underline{\xi }}_{\alpha \left(i\right)}^{q}\right)}^{\omega_{i}}+\prod_{i=1}^{n}\;{\left(\;{\underline{\xi }}_{\alpha \left(i\right)}^{q}\right)}^{\omega_{i}}},}\\ \sqrt[{q}]{\frac{\prod_{i=1}^{n}\;{\left(1+{\bar{\zeta }}_{\alpha \left(i\right)}^{q}\right)}^{\omega_{i}}-\prod_{i=1}^{n}\;{\left(1-{\bar{\zeta }}_{\alpha \left(i\right)}^{q}\right)}^{\omega_{i}}}{\prod_{i=1}^{n}\;{\left(1+{\bar{\zeta }}_{\alpha \left(i\right)}^{q}\right)}^{\omega_{i}}+{\prod_{i=1}^{n}\;\left(1-{\bar{\zeta }}_{\alpha \left(i\right)}^{q}\right)}^{\omega_{i}}}},\sqrt[{q}]{\frac{2{\prod_{i=1}^{n}\;\left({\bar{\eta }}_{\alpha \left(i\right)}^{q}\right)}^{\omega_{i}}}{{\prod_{i=1}^{n}\;\left(2-{\bar{\eta }}_{\alpha \left(i\right)}^{q}\right)}^{\omega_{i}}+\prod_{i=1}^{n}\;{\left({\bar{\eta }}_{\alpha \left(i\right)}^{q}\right)}^{\omega_{i}}}},\sqrt[{q}]{\frac{2{\prod_{i=1}^{n}\;\left(\;{\bar{\xi }}_{\alpha \left(i\right)}^{q}\right)}^{\omega_{i}}}{{\prod_{i=1}^{n}\;\left(2-\;{\bar{\xi }}_{\alpha \left(i\right)}^{q}\right)}^{\omega_{i}}+\prod_{i=1}^{n}\;{\left(\;{\bar{\xi }}_{\alpha \left(i\right)}^{q}\right)}^{\omega_{i}}}} \end{array}\right\rangle \right]$$

We are facing the following cases.

Case 1 If ${\underline{\zeta }}_{\alpha \left(i\right)}={\underline{\eta }}_{\alpha \left(i\right)}={\underline{\xi }}_{\alpha \left(i\right)}=0\;and\;q=2$ then

$$q-SFREHWA\left(A_{\alpha \left(1\right)},A_{\alpha \left(2\right)},\ldots ,A_{\alpha \left(n\right)}\right)={⊕}_{i=1}^{n}\left(\omega_{i}A_{\alpha \left(i\right)}\right)$$

$$=\left[\left\langle \sqrt{\frac{\prod_{i=1}^{n}\;{\left(1+{\bar{\zeta }}_{\alpha \left(i\right)}^{2}\right)}^{\omega_{i}}-\prod_{i=1}^{n}\;{\left(1-{\bar{\zeta }}_{\alpha \left(i\right)}^{2}\right)}^{\omega_{i}}}{\prod_{i=1}^{n}\;{\left(1+{\bar{\zeta }}_{\alpha \left(i\right)}^{2}\right)}^{\omega_{i}}+{\prod_{i=1}^{n}\;\left(1-{\bar{\zeta }}_{\alpha \left(i\right)}^{2}\right)}^{\omega_{i}}}},\sqrt{\frac{2{\prod_{i=1}^{n}\;\left({\bar{\eta }}_{\alpha \left(i\right)}^{2}\right)}^{\omega_{i}}}{{\prod_{i=1}^{n}\;\left(2-{\bar{\eta }}_{\alpha \left(i\right)}^{2}\right)}^{\omega_{i}}+\prod_{i=1}^{n}\;{\left({\bar{\eta }}_{\alpha \left(i\right)}^{2}\right)}^{\omega_{i}}}},\sqrt{\frac{2{\prod_{i=1}^{n}\;\left(\;{\bar{\xi }}_{\alpha \left(i\right)}^{2}\right)}^{\omega_{i}}}{{\prod_{i=1}^{n}\;\left(2-\;{\bar{\xi }}_{\alpha \left(i\right)}^{2}\right)}^{\omega_{i}}+\prod_{i=1}^{n}\;{\left({\bar{\xi }}_{\alpha \left(i\right)}^{2}\;\right)}^{\omega_{i}}}}\right\rangle \right]$$

$$=SFREHWA\left(A_{\alpha \left(1\right)},A_{\alpha \left(2\right)},\ldots ,A_{\alpha \left(n\right)}\right)={⊕}_{i=1}^{n}\left(\omega_{i}A_{\alpha \left(i\right)}\right)$$

(The spherical fuzzy rough Einstein hybrid weighted averaging operator).

Case 2 If ${\underline{\xi }}_{\alpha \left(i\right)}={\bar{\xi }}_{\alpha \left(i\right)}=0\;and\;q=2$ then

$$q-SFREHWA\left(A_{\alpha \left(1\right)},A_{\alpha \left(2\right)},\ldots ,A_{\alpha \left(n\right)}\right)={⊕}_{i=1}^{n}\left(\omega_{i}A_{\alpha \left(i\right)}\right)=\left[\left\langle \begin{array}{c} \sqrt{\frac{\prod_{i=1}^{n}\;{\left(1+{\underline{\zeta }}_{\alpha \left(i\right)}^{2}\right)}^{\omega_{i}}-\prod_{i=1}^{n}\;{\left(1-{\underline{\zeta }}_{\alpha \left(i\right)}^{2}\right)}^{\omega_{i}}}{\prod_{i=1}^{n}\;{\left(1+{\underline{\zeta }}_{\alpha \left(i\right)}^{2}\right)}^{\omega_{i}}+{\prod_{i=1}^{n}\;\left(1-{\underline{\zeta }}_{\alpha \left(i\right)}^{2}\right)}^{\omega_{i}}}},\sqrt{\frac{2{\prod_{i=1}^{n}\;\left({\underline{\eta }}_{\alpha \left(i\right)}^{q}\right)}^{\omega_{i}}}{{\prod_{i=1}^{n}\;\left(2-{\underline{\eta }}_{\alpha \left(i\right)}^{q}\right)}^{\omega_{i}}+\prod_{i=1}^{n}\;{\left({\underline{\eta }}_{\alpha \left(i\right)}^{q}\right)}^{\omega_{i}}}},\\ \sqrt{\frac{\prod_{i=1}^{n}\;{\left(1+{\bar{\zeta }}_{\alpha \left(i\right)}^{2}\right)}^{\omega_{i}}-\prod_{i=1}^{n}\;{\left(1-{\bar{\zeta }}_{\alpha \left(i\right)}^{2}\right)}^{\omega_{i}}}{\prod_{i=1}^{n}\;{\left(1+{\bar{\zeta }}_{\alpha \left(i\right)}^{2}\right)}^{\omega_{i}}+{\prod_{i=1}^{n}\;\left(1-{\bar{\zeta }}_{\alpha \left(i\right)}^{2}\right)}^{\omega_{i}}}},\sqrt{\frac{2{\prod_{i=1}^{n}\;\left({\bar{\eta }}_{\alpha \left(i\right)}^{2}\right)}^{\omega_{i}}}{{\prod_{i=1}^{n}\;\left(2-{\bar{\eta }}_{\alpha \left(i\right)}^{2}\right)}^{\omega_{i}}+\prod_{i=1}^{n}\;{\left({\bar{\eta }}_{\alpha \left(i\right)}^{2}\right)}^{\omega_{i}}}} \end{array}\right\rangle \right]=PyFREHWA\left(A_{\alpha \left(1\right)},A_{\alpha \left(2\right)},\ldots ,A_{\alpha \left(n\right)}\right)={⊕}_{i=1}^{n}\left(\omega_{i}A_{\alpha \left(i\right)}\right)$$

(The Pythagorean fuzzy rough Einstein hybrid weighted averaging operator).

Case 3 If q=1 then

$$q-SFREHWA\left(A_{\alpha \left(1\right)},A_{\alpha \left(2\right)},\ldots ,A_{\alpha \left(n\right)}\right)=\left[\left\langle \begin{array}{c} \frac{\prod_{i=1}^{n}\;{\left(1+{\underline{\zeta }}_{\alpha \left(i\right)}\right)}^{\omega_{i}}-\prod_{i=1}^{n}\;{\left(1-{\underline{\zeta }}_{\alpha \left(i\right)}\right)}^{\omega_{i}}}{\prod_{i=1}^{n}\;{\left(1+{\underline{\zeta }}_{\alpha \left(i\right)}\right)}^{\omega_{i}}+{\prod_{i=1}^{n}\;\left(1-{\underline{\zeta }}_{\alpha \left(i\right)}\right)}^{\omega_{i}}},\frac{2{\prod_{i=1}^{n}\;\left({\underline{\eta }}_{\alpha \left(i\right)}\right)}^{\omega_{i}}}{{\prod_{i=1}^{n}\;\left(2-{\underline{\eta }}_{\alpha \left(i\right)}\right)}^{\omega_{i}}+\prod_{i=1}^{n}\;{\left({\underline{\eta }}_{\alpha \left(i\right)}\right)}^{\omega_{i}}},\frac{2{\prod_{i=1}^{n}\;\left(\;{\underline{\xi }}_{\alpha \left(i\right)}\right)}^{\omega_{i}}}{{\prod_{i=1}^{n}\;\left(2-\;{\underline{\xi }}_{\alpha \left(i\right)}\right)}^{\omega_{i}}+\prod_{i=1}^{n}\;{\left({\underline{\xi }}_{\alpha \left(i\right)}\right)}^{\omega_{i}}}.\\ \frac{\prod_{i=1}^{n}\;{\left(1+{\bar{\zeta }}_{\alpha \left(i\right)}\right)}^{\omega_{i}}-\prod_{i=1}^{n}\;{\left(1-{\bar{\zeta }}_{\alpha \left(i\right)}\right)}^{\omega_{i}}}{\prod_{i=1}^{n}\;{\left(1+{\bar{\zeta }}_{\alpha \left(i\right)}\right)}^{\omega_{i}}+{\prod_{i=1}^{n}\;\left(1-{\bar{\zeta }}_{\alpha \left(i\right)}\right)}^{\omega_{i}}},\frac{2{\prod_{i=1}^{n}\;\left({\bar{\eta }}_{\alpha \left(i\right)}\right)}^{\omega_{i}}}{{\prod_{i=1}^{n}\;\left(2-{\bar{\eta }}_{\alpha \left(i\right)}\right)}^{\omega_{i}}+\prod_{i=1}^{n}\;{\left({\bar{\eta }}_{\alpha \left(i\right)}\right)}^{\omega_{i}}},\frac{2{\prod_{i=1}^{n}\;\left(\;{\bar{\xi }}_{\alpha \left(i\right)}\right)}^{\omega_{i}}}{{\prod_{i=1}^{n}\;\left(2-{\bar{\xi }}_{\alpha \left(i\right)}\right)}^{\omega_{i}}+\prod_{i=1}^{n}\;{\left(\;{\bar{\xi }}_{\alpha \left(i\right)}\right)}^{\omega_{i}}} \end{array}\right\rangle \right]$$

$$=PFREHWA\left(A_{\alpha \left(1\right)},A_{\alpha \left(2\right)},\ldots ,A_{\alpha \left(n\right)}\right)={⊕}_{i=1}^{n}\left(\omega_{i}A_{\alpha \left(i\right)}\right)$$

(The picture fuzzy rough Einstein hybrid weighted averaging operator).

Case 4 If $\text{If}\;{\underline{\xi }}_{\alpha \left(i\right)}={\bar{\xi }}_{\alpha \left(i\right)}=0\;and\;q=1$ then

$$q-SFREHWA\left(A_{\alpha \left(1\right)},A_{\alpha \left(2\right)},\ldots ,A_{\alpha \left(n\right)}\right)=\left[\left\langle \begin{array}{c} \frac{\prod_{i=1}^{n}\;{\left(1+{\underline{\zeta }}_{\alpha \left(i\right)}\right)}^{\omega_{i}}-\prod_{i=1}^{n}\;{\left(1-{\underline{\zeta }}_{\alpha \left(i\right)}\right)}^{\omega_{i}}}{\prod_{i=1}^{n}\;{\left(1+{\underline{\zeta }}_{\alpha \left(i\right)}\right)}^{\omega_{i}}+{\prod_{i=1}^{n}\;\left(1-{\underline{\zeta }}_{\alpha \left(i\right)}\right)}^{\omega_{i}}},\frac{2{\prod_{i=1}^{n}\;\left({\underline{\eta }}_{\alpha \left(i\right)}\right)}^{\omega_{i}}}{{\prod_{i=1}^{n}\;\left(2-{\underline{\eta }}_{\alpha \left(i\right)}\right)}^{\omega_{i}}+\prod_{i=1}^{n}\;{\left({\underline{\eta }}_{\alpha \left(i\right)}\right)}^{\omega_{i}}}.\\ \frac{\prod_{i=1}^{n}\;{\left(1+{\bar{\zeta }}_{\alpha \left(i\right)}\right)}^{\omega_{i}}-\prod_{i=1}^{n}\;{\left(1-{\bar{\zeta }}_{\alpha \left(i\right)}\right)}^{\omega_{i}}}{\prod_{i=1}^{n}\;{\left(1+{\bar{\zeta }}_{\alpha \left(i\right)}\right)}^{\omega_{i}}+{\prod_{i=1}^{n}\;\left(1-{\bar{\zeta }}_{\alpha \left(i\right)}\right)}^{\omega_{i}}},\frac{2{\prod_{i=1}^{n}\;\left({\bar{\eta }}_{\alpha \left(i\right)}\right)}^{\omega_{i}}}{{\prod_{i=1}^{n}\;\left(2-{\bar{\eta }}_{\alpha \left(i\right)}\right)}^{\omega_{i}}+\prod_{i=1}^{n}\;{\left({\bar{\eta }}_{\alpha \left(i\right)}\right)}^{\omega_{i}}} \end{array}\right\rangle \right]$$

$$=IFREHWA\left(A_{\alpha \left(1\right)},A_{\alpha \left(2\right)},\ldots ,A_{\alpha \left(n\right)}\right)={⊕}_{i=1}^{n}\left(\omega_{i}A_{\alpha \left(i\right)}\right)$$

(The intuitionistic fuzzy rough Einstein hybrid weighted averaging operator).

**Fig. 6:**
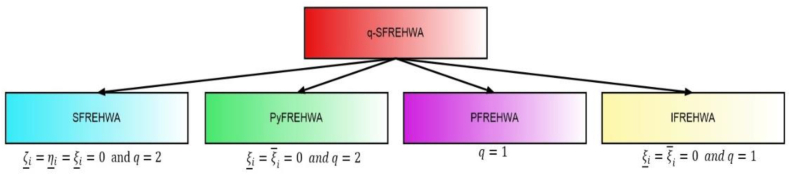
Specificcasesregardingtheq−SFREHWAoperator.

Fig. 6 represents specificcasesregardingthe q−SFREHWAoperator.

Example 4 Consider four q-SFRNs $A_{1}=\left(0.4,0.1,0.3,0.2,0.4,0.3\right)$, $A_{2}=\left(0.5,0.9,0.5,0.8,0.6,0.2\right)\text{,}$ $A_{3}=\left(0.5,0.9,0.4,0.2,0.6,0.3\right)\text{,}$ and $A_{4}=\left(0.2,0.5,0.5,0.8,0.9,0.3\right)\text{.}$ If ω= ${\left(0.3,0.1,0.4,0.2\right)}^{T}$ and q=4, then the q-SFREHWA operator, as defined in Definition (16), can be computed as:

$${A_{1}}^{*}=\left[\left\langle \begin{array}{c} \sqrt[{q}]{\frac{{\left(1+{\underline{\zeta }}_{1}^{q}\right)}^{{\sigma \times \omega }_{1}}-{\left(1-{\underline{\zeta }}_{1)}^{q}\right)}^{{\sigma \times \omega }_{1}}}{{\left(1+{\underline{\zeta }}_{1}^{q}\right)}^{{\sigma \times \omega }_{1}}+{\left(1-{\underline{\zeta }}_{1}^{q}\right)}^{{\sigma \times \omega }_{1}}}},\sqrt[{q}]{\frac{2{\left({\underline{\eta }}_{1}^{q}\right)}^{{\sigma \times \omega }_{1}}}{{\left(2-{\underline{\eta }}_{1}^{q}\right)}^{{\sigma \times \omega }_{1}}+{\left({\underline{\eta }}_{1}^{q}\right)}^{{\sigma \times \omega }_{1}}}},\sqrt[{q}]{\frac{2{\left(\;{\underline{\xi }}_{1}^{q}\right)}^{{\sigma \times \omega }_{1}}}{{\left(2-\;{\underline{\xi }}_{1}^{q}\right)}^{{\sigma \times \omega }_{1}}+{\left(\;{\underline{\xi }}_{1}^{q}\right)}^{{\sigma \times \omega }_{1}}},}\\ \sqrt[{q}]{\frac{{\left(1+{\bar{\zeta }}_{1}^{q}\right)}^{{\sigma \times \omega }_{1}}-{\left(1-{\bar{\zeta }}_{1}^{q}\right)}^{{\sigma \times \omega }_{1}}}{{\left(1+{\bar{\zeta }}_{1}^{q}\right)}^{{\sigma \times \omega }_{1}}+{\left(1-{\bar{\zeta }}_{1}^{q}\right)}^{{\sigma \times \omega }_{1}}}},\sqrt[{q}]{\frac{2{\left({\bar{\eta }}_{1}^{q}\right)}^{{\sigma \times \omega }_{1}}}{{\left(2-{\bar{\eta }}_{1}^{q}\right)}^{{\sigma \times \omega }_{1}}+{\left({\bar{\eta }}_{1}^{q}\right)}^{{\sigma \times \omega }_{1}}}},\sqrt[{q}]{\frac{2{\left({\bar{\xi }}_{1}^{q}\right)}^{{\sigma \times \omega }_{1}}}{{\left(2-{\bar{\xi }}_{1}^{q}\right)}^{{\sigma \times \omega }_{1}}+{\left(\;{\bar{\xi }}_{1}^{q}\right)}^{{\sigma \times \omega }_{1}}}} \end{array}\right\rangle \right]$$

=(0.4250,0.0603,0.2259,0.2125,0.3204,0.2259)

$${A_{2}}^{*}=\left[\left\langle \begin{array}{c} \sqrt[{q}]{\frac{{\left(1+{\underline{\zeta }}_{2}^{q}\right)}^{{\sigma \times \omega }_{2}}-{\left(1-{\underline{\zeta }}_{2}^{q}\right)}^{{\sigma \times \omega }_{2}}}{{\left(1+{\underline{\zeta }}_{2}^{q}\right)}^{{\sigma \times \omega }_{2}}+{\left(1-{\underline{\zeta }}_{2}^{q}\right)}^{{\sigma \times \omega }_{2}}}},\sqrt[{q}]{\frac{2{\left({\underline{\eta }}_{2}^{q}\right)}^{{\sigma \times \omega }_{2}}}{{\left(2-{\underline{\eta }}_{2}^{q}\right)}^{{\sigma \times \omega }_{2}}+{\left({\underline{\eta }}_{2}^{q}\right)}^{{\sigma \times \omega }_{2}}}},\sqrt[{q}]{\frac{2{\left(\;{\underline{\xi }}_{2}^{q}\right)}^{{\sigma \times \omega }_{2}}}{{\left(2-\;{\underline{\xi }}_{2}^{q}\right)}^{{\sigma \times \omega }_{2}}+{\left(\;{\underline{\xi }}_{2}^{q}\right)}^{{\sigma \times \omega }_{2}}},}\\ \sqrt[{q}]{\frac{{\left(1+{\bar{\zeta }}_{2}^{q}\right)}^{{\sigma \times \omega }_{2}}-{\left(1-{\bar{\zeta }}_{2}^{q}\right)}^{{\sigma \times \omega }_{2}}}{{\left(1+{\bar{\zeta }}_{2}^{q}\right)}^{{\sigma \times \omega }_{2}}+{\left(1-{\bar{\zeta }}_{2}^{q}\right)}^{{\sigma \times \omega }_{2}}}},\sqrt[{q}]{\frac{2{\left({\bar{\eta }}_{2}^{q}\right)}^{{\sigma \times \omega }_{2}}}{{\left(2-{\bar{\eta }}_{2}^{q}\right)}^{{\sigma \times \omega }_{2}}+{\left({\bar{\eta }}_{2}^{q}\right)}^{{\sigma \times \omega }_{2}}}},\sqrt[{q}]{\frac{2{\left({\bar{\xi }}_{2}^{q}\right)}^{{\sigma \times \omega }_{2}}}{{\left(2-{\bar{\xi }}_{2}^{q}\right)}^{{\sigma \times \omega }_{2}}+{\left({\bar{\xi }}_{2}^{q}\right)}^{{\sigma \times \omega }_{2}}}} \end{array}\right\rangle \right]$$

=(0.3689,0.9616,0.7968,0.6059,0.8440,0.5831)

$${A_{3}}^{*}=\left[\left\langle \begin{array}{c} \sqrt[{q}]{\frac{{\left(1+{\underline{\zeta }}_{3}^{q}\right)}^{{\sigma \times \omega }_{3}}-{\left(1-{\underline{\zeta }}_{3}^{q}\right)}^{{\sigma \times \omega }_{3}}}{{\left(1+{\underline{\zeta }}_{3}^{q}\right)}^{{\sigma \times \omega }_{3}}+{\left(1-{\underline{\zeta }}_{3}^{q}\right)}^{{\sigma \times \omega }_{3}}}},\sqrt[{q}]{\frac{2{\left({\underline{\eta }}_{3}^{q}\right)}^{{\sigma \times \omega }_{3}}}{{\left(2-{\underline{\eta }}_{3}^{q}\right)}^{{\sigma \times \omega }_{3}}+{\left({\underline{\eta }}_{3}^{q}\right)}^{{\sigma \times \omega }_{3}}}},\sqrt[{q}]{\frac{2{\left(\;{\underline{\xi }}_{3}^{q}\right)}^{{\sigma \times \omega }_{3}}}{{\left(2-\;{\underline{\xi }}_{3}^{q}\right)}^{{\sigma \times \omega }_{3}}+{\left(\;{\underline{\xi }}_{3}^{q}\right)}^{{\sigma \times \omega }_{3}}},}\\ \sqrt[{3}]{\frac{{\left(1+{\bar{\zeta }}_{3}^{q}\right)}^{{\sigma \times \omega }_{3}}-\prod_{i=1}^{n}\;{\left(1-{\bar{\zeta }}_{3}^{q}\right)}^{{\sigma \times \omega }_{3}}}{{\left(1+{\bar{\zeta }}_{3}^{q}\right)}^{{\sigma \times \omega }_{3}}+{\prod_{i=1}^{n}\;\left(1-{\bar{\zeta }}_{3}^{q}\right)}^{{\sigma \times \omega }_{3}}}},\sqrt[{q}]{\frac{2{\left({\bar{\eta }}_{3}^{q}\right)}^{{\sigma \times \omega }_{3}}}{{\left(2-{\bar{\eta }}_{3}^{q}\right)}^{{\sigma \times \omega }_{3}}+{\left({\bar{\eta }}_{3}^{q}\right)}^{{\sigma \times \omega }_{3}}}},\sqrt[{q}]{\frac{2{\left({\bar{\xi }}_{3}^{q}\right)}^{{\sigma \times \omega }_{3}}}{{\left(2-{\bar{\xi }}_{3}^{q}\right)}^{{\sigma \times \omega }_{3}}+{\left({\bar{\xi }}_{3}^{q}\right)}^{{\sigma \times \omega }_{3}}}} \end{array}\right\rangle \right]$$

=(0.5832,0.8351,0.2042,0.2339,0.4041,0.1277)

$${A_{4}}^{*}=\left[\left\langle \begin{array}{c} \sqrt[{q}]{\frac{{\left(1+{\underline{\zeta }}_{4}^{q}\right)}^{{\sigma \times \omega }_{4}}-{\left(1-{\underline{\zeta }}_{4}^{q}\right)}^{{\sigma \times \omega }_{4}}}{{\left(1+{\underline{\zeta }}_{4}^{q}\right)}^{{\sigma \times \omega }_{4}}+{\left(1-{\underline{\zeta }}_{4}^{q}\right)}^{{\sigma \times \omega }_{4}}}},\sqrt[{q}]{\frac{2{\left({\underline{\eta }}_{4}^{q}\right)}^{{\sigma \times \omega }_{4}}}{{\left(2-{\underline{\eta }}_{4}^{q}\right)}^{{\sigma \times \omega }_{4}}+{\left({\underline{\eta }}_{4}^{q}\right)}^{{\sigma \times \omega }_{4}}}},\sqrt[{q}]{\frac{2{\left(\;{\underline{\xi }}_{4}^{q}\right)}^{{\sigma \times \omega }_{4}}}{{\left(2-\;{\underline{\xi }}_{4}^{q}\right)}^{{\sigma \times \omega }_{4}}+{\left(\;{\underline{\xi }}_{4}^{q}\right)}^{{\sigma \times \omega }_{4}}},}\\ \sqrt[{q}]{\frac{{\left(1+{\bar{\zeta }}_{4}^{q}\right)}^{{\sigma \times \omega }_{4}}-{\left(1-{\bar{\zeta }}_{4}^{q}\right)}^{{\sigma \times \omega }_{4}}}{{\left(1+{\bar{\zeta }}_{4}^{q}\right)}^{{\sigma \times \omega }_{4}}+{\left(1-{\bar{\zeta }}_{4}^{q}\right)}^{{\sigma \times \omega }_{4}}}},\sqrt[{q}]{\frac{2{\left({\bar{\eta }}_{4}^{q}\right)}^{{\sigma \times \omega }_{4}}}{{\left(2-{\bar{\eta }}_{4}^{q}\right)}^{{\sigma \times \omega }_{4}}+{\left({\bar{\eta }}_{4}^{q}\right)}^{{\sigma \times \omega }_{4}}}},\sqrt[{q}]{\frac{2{\left({\bar{\xi }}_{4}^{q}\right)}^{{\sigma \times \omega }_{4}}}{{\left(2-{\bar{\xi }}_{4}^{q}\right)}^{{\sigma \times \omega }_{4}}+{\left(\;{\bar{\xi }}_{4}^{q}\right)}^{{\sigma \times \omega }_{4}}}} \end{array}\right\rangle \right]$$

=(0.1857,0.5902,0.5902,0.7512,0.9210,0.3970)

Now we calculate the score values of ${A_{i}}^{*}\left(i=1,2,3,4\right)$.

$$Sco\left({A_{1}}^{*}\right)=\frac{{2+\left(0.4250\right)}^{3}+{\left(0.2125\right)}^{3}-{\left(0.0603\right)}^{3}-{\left(0.3204\right)}^{3}-{\left(0.2259\right)}^{3}-{\left(0.2259\right)}^{3}}{3}=\left(0.6767\right)$$

$$Sco\left({A_{2}}^{*}\right)=\frac{{2+\left(0.3689\right)}^{3}+{\left(\;0.6059\right)}^{3}-{\left(0.9616\right)}^{3}-{\left(0.8440\right)}^{3}-{\left(0.7968\right)}^{3}-{\left(0.5831\right)}^{3}}{3}=\left(0.0260\right)$$

$$Sco\left({A_{3}}^{*}\right)=\frac{{2+\left(0.5832\right)}^{3}+{\left(0.2339\right)}^{3}-{\left(0.8351\right)}^{3}-{\left(0.4041\right)}^{3}-{\left(0.2042\right)}^{3}-{\left(0.1277\right)}^{3}}{3}=\left(0.5174\right)$$

$$Sco\left({A_{4}}^{*}\right)=\frac{{2+\left(0.1857\right)}^{3}+{\left(0.7512\right)}^{3}-{\left(0.5902\right)}^{3}-{\left(0.9210\right)}^{3}-{\left(0.5902\right)}^{3}-{\left(0.3970\right)}^{3}}{3}=\left(\;0.3918\right)$$

Since $Sco\left({A_{1}}^{*}\right)>\;Sco\left({A_{3}}^{*}\right)>\;Sco\left({A_{4}}^{*}\right)>\;Sco\left({A_{2}}^{*}\right)$.

$$\left({A_{1}}^{*}\right)>\;\left({A_{3}}^{*}\right)>\;\left({A_{4}}^{*}\right)>\left({A_{2}}^{*}\right)$$

Hence

$$A_{\alpha \left(1\right)}=A_{1}^{\circ }=\left(0.4250,0.0603,0.2259,0.2125,0.3204,0.2259\right)$$

$$A_{\alpha \left(2\right)}=A_{2}^{\circ }=\left(0.5832,0.8351,0.2042,0.2339,0.4041,0.1277\right)$$

$$A_{\alpha \left(3\right)}=A_{3}^{\circ }=\left(0.1857,0.5902,0.5902,0.7512,0.9210,0.3970\right)$$

$$A_{\alpha \left(4\right)}=A_{4}^{\circ }=\left(0.3689,0.9616,0.7968,0.6059,0.8440,0.5831\right)$$

Hence for $A_{\alpha \left(i\right)}\left(i=1,2,3,4\right)\text{,}$ we have

$$q-SFREHWA\left(\;A_{\alpha \left(1\right)},A_{\alpha \left(2\right)},A_{\alpha \left(3\right)},A_{\alpha \left(4\right)}\right)=\left[\left\langle \begin{array}{c} \sqrt[{q}]{\frac{\prod_{i=1}^{4}\;{\left(1+{\underline{\zeta }}_{\alpha \left(i\right)}^{q}\right)}^{\omega_{i}}-\prod_{i=1}^{4}\;{\left(1-{\underline{\zeta }}_{\alpha \left(i\right)}^{q}\right)}^{\omega_{i}}}{\prod_{i=1}^{4}\;{\left(1+{\underline{\zeta }}_{\alpha \left(i\right)}^{q}\right)}^{\omega_{i}}+{\prod_{i=1}^{4}\;\left(1-{\underline{\zeta }}_{\alpha \left(i\right)}^{q}\right)}^{\omega_{i}}}},\sqrt[{q}]{\frac{2{\prod_{i=1}^{4}\;\left({\underline{\eta }}_{\alpha \left(i\right)}^{q}\right)}^{\omega_{i}}}{{\prod_{i=1}^{4}\;\left(2-{\underline{\eta }}_{\alpha \left(i\right)}^{q}\right)}^{\omega_{i}}+\prod_{i=1}^{4}\;{\left({\underline{\eta }}_{\alpha \left(i\right)}^{q}\right)}^{\omega_{i}}}},\sqrt[{q}]{\frac{2{\prod_{i=1}^{4}\;\left(\;{\underline{\xi }}_{\alpha \left(i\right)}^{q}\right)}^{\omega_{i}}}{{\prod_{i=1}^{4}\;\left(2-\;{\underline{\xi }}_{\alpha \left(i\right)}^{q}\right)}^{\omega_{i}}+\prod_{i=1}^{4}\;{\left(\;{\underline{\xi }}_{\alpha \left(i\right)}^{q}\right)}^{\omega_{i}}},}\\ \sqrt[{q}]{\frac{\prod_{i=1}^{4}\;{\left(1+{\bar{\zeta }}_{\alpha \left(i\right)}^{q}\right)}^{\omega_{i}}-\prod_{i=1}^{4}\;{\left(1-{\bar{\zeta }}_{\alpha \left(i\right)}^{q}\right)}^{\omega_{i}}}{\prod_{i=1}^{4}\;{\left(1+{\bar{\zeta }}_{\alpha \left(i\right)}^{q}\right)}^{\omega_{i}}+{\prod_{i=1}^{4}\;\left(1-{\bar{\zeta }}_{\alpha \left(i\right)}^{q}\right)}^{\omega_{i}}}},\sqrt[{q}]{\frac{2{\prod_{i=1}^{4}\;\left({\bar{\eta }}_{\alpha \left(i\right)}^{q}\right)}^{\omega_{i}}}{{\prod_{i=1}^{4}\;\left(2-{\bar{\eta }}_{\alpha \left(i\right)}^{q}\right)}^{\omega_{i}}+\prod_{i=1}^{4}\;{\left({\bar{\eta }}_{\alpha \left(i\right)}^{q}\right)}^{\omega_{i}}}},\sqrt[{q}]{\frac{2{\prod_{i=1}^{4}\;\left(\;{\bar{\xi }}_{\alpha \left(i\right)}^{q}\right)}^{\omega_{i}}}{{\prod_{i=1}^{4}\;\left(2-\;{\bar{\xi }}_{\alpha \left(i\right)}^{q}\right)}^{\omega_{i}}+\prod_{i=1}^{4}\;{\left(\;{\bar{\xi }}_{\alpha \left(i\right)}^{q}\right)}^{\omega_{i}}}} \end{array}\right\rangle \right]=\left(\;0.3820,0.6335,0.6569,0.6095,0.7606,0.5918\right)\text{.}$$

## 4 An application of the proposed aggregation operators

This section emphasizes the resolution of multi-attribute decision-making (MADM) problems utilizing the operators and q-SFR numbers described earlier. An example is provided to illustrate the efficacy and application of these operators in practical scenarios.

Consider the following sets: V = {$V_{1},V_{2},V_{3},\ldots ,V_{m}$} for the m alternatives, J = {$J_{1},J_{2},J_{3},{\ldots ,J}_{n}$} for n criteria, and D = {$D_{1}$, $D_{2}$, $D_{3}\text{,}$., $D_{k}$} for k experts. Consider the corresponding weight vector for alternatives as $\omega ={\left(\omega_{1},\omega_{2},\ldots ,\omega_{n}\right)}^{T}$. Let $\lambda ={\left(\lambda_{1},\lambda_{2},\ldots ,\lambda_{n}\right)}^{T}$ represent the weighted vector for experts D = {$D_{1}$, $D_{2}$, $D_{3}\text{,}$., $D_{k}$}. Both weight vectors meet the identical requirements and are in the closed interval [0,1], with their sum equal to one. Let $A_{ij}=\left({\underline{\zeta }}_{ij},{\underline{\eta }}_{ij},{\underline{\xi }}_{ij},{\bar{\zeta }}_{ij},{\bar{\eta }}_{ij},{\bar{\xi }}_{ij}\right)\;\text{for}\;\left(i=1,2,\ldots ,n\right)$ and (j=1,2,…,m), where (${\underline{\zeta }}_{i},{\underline{\eta }}_{i},{\underline{\xi }}_{i}$) and (${\bar{\zeta }}_{i},{\bar{\eta }}_{i},{\bar{\xi }}_{i}$) represents lower set approximation and upper aet approximation, subject to the constraint $\left(0\leq {{\underline{\zeta }}_{ij}}^{q}+{{\underline{\eta }}_{ij}}^{q}+{{\underline{\xi }}_{ij}}^{q}\leq 1\right)$ and $\left(0\leq {{\bar{\zeta }}_{ij}}^{q}+{{\bar{\eta }}_{ij}}^{q}+{{\bar{\xi }}_{ij}}^{q}\leq 1\right)$. Following is the procedure to solve an MCDM problem.

Step 1**:** Construct $D^{\left(k\right)}={\left[\left(A_{ij}^{\left(k\right)}\right)\right]}_{m\times n}$ (k=1,2,3,…,d) for decision.

Step 2**:** If the criteria have two types, such as benefit criteria and cost criteria, the $D^{\left(k\right)}={\left[\left(A_{ij}^{\left(k\right)}\right)\right]}_{m\times n}$ (k=1,2,3,…,d) van be converted into the normalized decision matrices $R^{\left(s\right)}$ = (s=1,2,3,…,t) where

$$r^{\left(s\right)}=\left\{ \begin{array}{c} A_{ij}^{\left(k\right)}\;\text{for}\;\text{benefit}\;\text{type}\;\text{of}\;\text{criteria}\\ {\left[\left(A_{ij}^{\left(k\right)}\right)\right]}^{C}\text{for}\;\text{cost}\;\text{type}\;\text{of}\;\text{criteria} \end{array}\right.$$

Where ${\left[\left(A_{ij}^{\left(k\right)}\right)\right]}^{C}$ is a complement of $A_{ij}^{\left(k\right)}$.

Step 3**:** Utilize the proposed operators to aggregate $R^{\left(k\right)}={\left[\left(A_{ij}^{\left(k\right)}\right)\right]}_{m\times n}$ into R = ${\left[A_{ij}\right]}_{m\times n}$.

**Step 4:** Utilize the ${\bar{A}}_{ij}$ = $\sigma {\bar{\lambda }}_{i}A_{ij}$.

**Step 5:** Utilize the proposed operator to derive the overall preference values**.**

**Step 6:** Calculate the scores of all values**.**

**Step 7:** Select the alternative which has the highest score value**.**

**Fig. 7:**
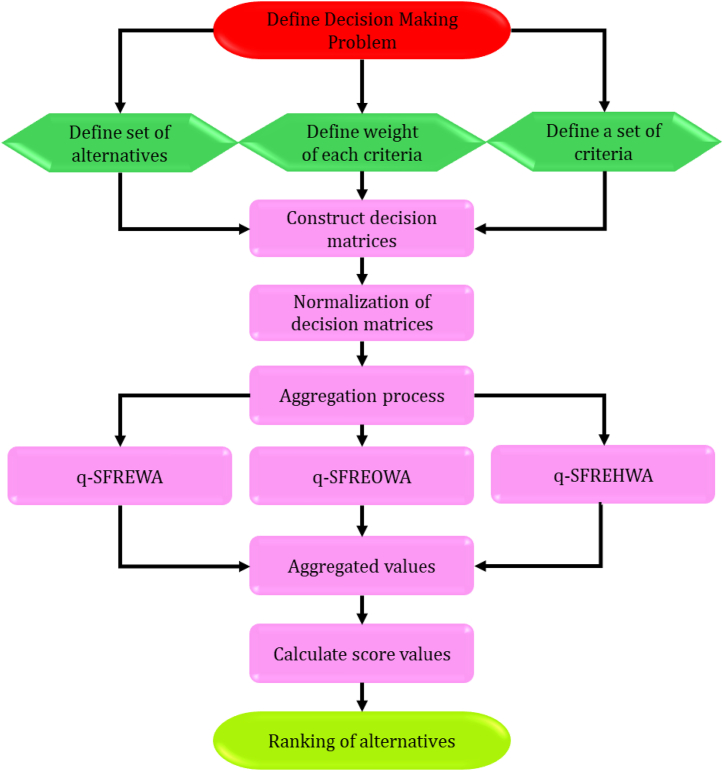
Flow chart of the proposed model.

The flow chart of the proposed model is shown in Fig. 7.

### 4.1 Numericalexample

**Fig. 8:**
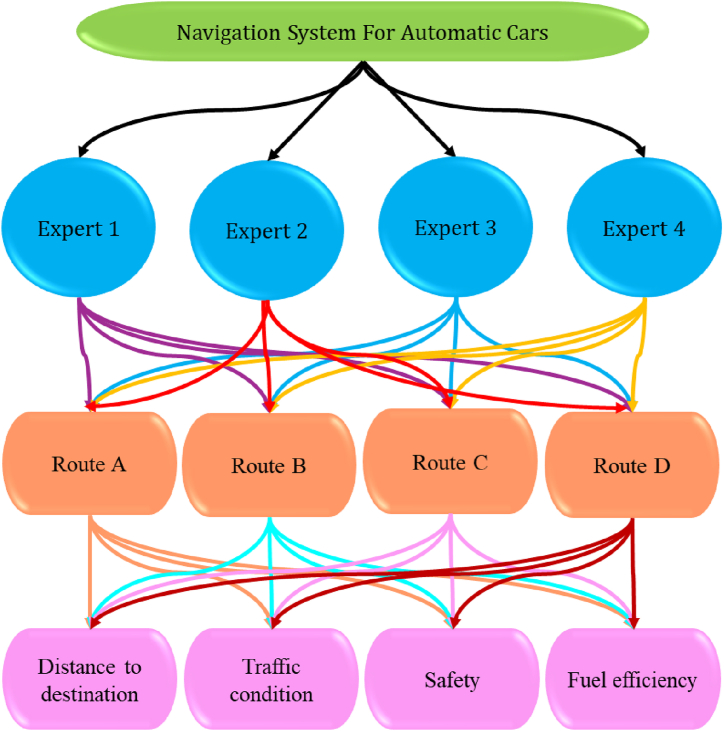
Navigation system for automatic cars.

To clarify and demonstrate the proposed procedure, we present a case in this segment. In this situation, let a major basiness company is assessing a q-SFR-based navigation system for automatic cars using four criteria $J_{1},J_{2},J_{3},J_{4}$ and four alternatives $V_{1},V_{2},V_{3},V_{4}$. The criteria are: $J_{1}=$ Distance to destination, $J_{2}=$ Traffic condition, $J_{3}=$ Safety and $J_{4}=$ Fuel efficiency. The alternatives are ${\;V}_{1}=$ Route A, ${\;V}_{2}=$ Route B, $V_{3}=$ Route C and $V_{4}=$ Route D. A set of four professionals assigns weights to these criteria, denoted as $\omega ={\left(0.3,0.1,0.4,0.2\right)}^{T}$. The weight vector reflects the significance of each criterion as assessed by the professionals. Next, we make a decision matrix $D^{\left(k\right)}=\left(\alpha_{ij}^{\left(k\right)}\right)$ (k=1,2,3,4). Table 1, Table 2, Table 3, Table 4 provide the decision matrices for evaluating the navigation systems for automatic cars. The goal is to rank these automatic cars and choose the best car. Fig. 8 displays a decision tree used in the navigation system to simplify the process of picking an automatic car.

**Table 1:** Decision matrix $D^{1}$.

**Alternatives**	$J_{1}$	$J_{2}$	$J_{3}$	$J_{4}$
$V_{1}$	$\left(\begin{array}{c} 0.56,0.53,0.95\text{,}\\ 0.55,0.45,0.16 \end{array}\right)$	$\left(\begin{array}{c} 0.29,0.65,0.65\text{,}\\ 0.28,0.08,0.36 \end{array}\right)$	$\left(\begin{array}{c} 0.95,0.16,0.95\text{,}\\ 0.05,0.16,0.65 \end{array}\right)$	$\left(\begin{array}{c} 0.96,0.75,0.79\text{,}\\ 0.32,0.73,0.63 \end{array}\right)$
$V_{2}$	$\left(\begin{array}{c} 0.66,0.89,0.72\text{,}\\ 0.95,0.85,0.45 \end{array}\right)$	$\left(\begin{array}{c} 0.05,0.06,0.26\text{,}\\ 0.56,0.06,0.98 \end{array}\right)$	$\left(\begin{array}{c} 0.95,0.45,0.35\text{,}\\ 0.19,0.45,0.55 \end{array}\right)$	$\left(\begin{array}{c} 0.45,0.66,0.96\text{,}\\ 0.64,0.54,0.23 \end{array}\right)$
$V_{3}$	$\left(\begin{array}{c} 0.25,0.26,0.73\text{,}\\ 0.65,0.95,0.26 \end{array}\right)$	$\left(\begin{array}{c} 0.98,0.68,0.19\text{,}\\ 0.68,0.28,0.79 \end{array}\right)$	$\left(\begin{array}{c} 0.15,0.28,0.23\text{,}\\ 0.31,0.25,0.18 \end{array}\right)$	$\left(\begin{array}{c} 0.75,0.46,0.35\text{,}\\ 0.86,0.35,0.83 \end{array}\right)$
$V_{4}$	$\left(\begin{array}{c} 0.65,0.26,0.25\text{,}\\ 0.95,0.85,0.25 \end{array}\right)$	$\left(\begin{array}{c} 0.98,0.95,0.15\text{,}\\ 0.95,0.75,0.15 \end{array}\right)$	$\left(\begin{array}{c} 0.26,0.83,0.16\text{,}\\ 0.29,0.93,0.86 \end{array}\right)$	$\left(\begin{array}{c} 0.96,0.36,0.13\text{,}\\ 0.85,0.25,0.75 \end{array}\right)$

**Table 2:** Decision matrix $D^{2}$.

**Alternatives**	$J_{1}$	$J_{2}$	$J_{3}$	$J_{4}$
$V_{1}$	$\left(\begin{array}{c} 0.85,0.83,0.27\text{,}\\ 0.35,0.25,0.25 \end{array}\right)$	$\left(\begin{array}{c} 0.24,0.17,0.23\text{,}\\ 0.20,0.94,0.22 \end{array}\right)$	$\left(\begin{array}{c} 0.82,0.83,0.92\text{,}\\ 0.85,0.39,0.80 \end{array}\right)$	$\left(\begin{array}{c} 0.62,0.36,0.35\text{,}\\ 0.75,0.52,0.65 \end{array}\right)$
$V_{2}$	$\left(\begin{array}{c} 0.15,0.32,0.59\text{,}\\ 0.12,0.62,0.92 \end{array}\right)$	$\left(\begin{array}{c} 0.95,0.36,0.82\text{,}\\ 0.76,0.69,0.47 \end{array}\right)$	$\left(\begin{array}{c} 0.29,0.61,0.56\text{,}\\ 0.20,0.86,0.25 \end{array}\right)$	$\left(\begin{array}{c} 0.38,0.65,0.72\text{,}\\ 0.82,0.25,0.32 \end{array}\right)$
$V_{3}$	$\left(\begin{array}{c} 0.66,0.62,0.59\text{,}\\ 0.26,0.35,0.94 \end{array}\right)$	$\left(\begin{array}{c} 0.57,0.73,0.27\text{,}\\ 0.73,0.69,0.22 \end{array}\right)$	$\left(\begin{array}{c} 0.66,0.37,0.35\text{,}\\ 0.26,0.86,0.96 \end{array}\right)$	$\left(\begin{array}{c} 0.79,0.23,0.55\text{,}\\ 0.96,0.86,0.29 \end{array}\right)$
$V_{4}$	$\left(\begin{array}{c} 0.06,0.45,0.99\text{,}\\ 0.56,0.57,0.97 \end{array}\right)$	$\left(\begin{array}{c} 0.27,0.86,0.27\text{,}\\ 0.92,0.26,0.53 \end{array}\right)$	$\left(\begin{array}{c} 0.88,0.65,0.23\text{,}\\ 0.12,0.55,0.49 \end{array}\right)$	$\left(\begin{array}{c} 0.69,0.67,0.59\text{,}\\ 0.82,0.63,0.31 \end{array}\right)$

**Table 3:** Decision matrix $D^{3}$.

**Alternatives**	$J_{1}$	$J_{2}$	$J_{3}$	$J_{4}$
$V_{1}$	$\left(\begin{array}{c} 0.85,0.96,0.08\\ 0.65,0.65,0.19 \end{array}\right)$	$\left(\begin{array}{c} 0.85,0.10,0.56\text{,}\\ 0.48,0.08,0.39 \end{array}\right)$	$\left(\begin{array}{c} 0.54,0.89,0.32\text{,}\\ 0.89,0.56,0.23 \end{array}\right)$	$\left(\begin{array}{c} 0.89,0.30,0.59\text{,}\\ 0.25,0.50,0.20 \end{array}\right)$
$V_{2}$	$\left(\begin{array}{c} 0.78,0.21,0.89\text{,}\\ 0.12,0.98,0.45 \end{array}\right)$	$\left(\begin{array}{c} 0.48,0.29,0.52\text{,}\\ 0.68,0.78,0.35 \end{array}\right)$	$\left(\begin{array}{c} 0.36,0.74,0.56\text{,}\\ 0.23,0.89,0.25 \end{array}\right)$	$\left(\begin{array}{c} 0.65,0.30,0.58\text{,}\\ 0.39,0.51,0.70 \end{array}\right)$
$V_{3}$	$\left(\begin{array}{c} 0.41,0.85,0.78\text{,}\\ 0.01,0.02,0.69 \end{array}\right)$	$\left(\begin{array}{c} 0.28,0.69,0.57\text{,}\\ 0.69,0.85,0.32 \end{array}\right)$	$\left(\begin{array}{c} 0.78,0.63,0.74\text{,}\\ 0.23,0.65,0.25 \end{array}\right)$	$\left(\begin{array}{c} 0.23,0.30,0.90\text{,}\\ 0.39,0.50,0.39 \end{array}\right)$
$V_{4}$	$\left(\begin{array}{c} 0.78,0.30,0.56\text{,}\\ 0.03,0.09,0.11 \end{array}\right)$	$\left(\begin{array}{c} 0.62,0.69,0.58\text{,}\\ 0.96,0.85,0.35 \end{array}\right)$	$\left(\begin{array}{c} 0.32,0.56,0.23\text{,}\\ 0.25,0.58,0.52 \end{array}\right)$	$\left(\begin{array}{c} 0.32,0.30,0.54\text{,}\\ 0.56,0.85,0.85 \end{array}\right)$

**Table 4:** Decision matrix $D^{4}$.

**Alternatives**	$J_{1}$	$J_{2}$	$J_{3}$	$J_{4}$
$V_{1}$	$\left(\begin{array}{c} 0.84,0.91,0.53\text{,}\\ 0.45,0.23,0.35 \end{array}\right)$	$\left(\begin{array}{c} 0.54,0.37,0.53\text{,}\\ 0.39,0.52,0.37 \end{array}\right)$	$\left(\begin{array}{c} 0.57,0.38,0.55\text{,}\\ 0.39,0.53,0.37 \end{array}\right)$	$\left(\begin{array}{c} 0.56,0.31,0.56\text{,}\\ 0.34,0.53,0.34 \end{array}\right)$
$V_{2}$	$\left(\begin{array}{c} 0.91,0.25,0.54\text{,}\\ 0.85,0.72,0.31 \end{array}\right)$	$\left(\begin{array}{c} 0.56,0.36,0.54\text{,}\\ 0.36,0.57,0.36 \end{array}\right)$	$\left(\begin{array}{c} 0.60,0.36,0.56\text{,}\\ 0.80,0.54,0.36 \end{array}\right)$	$\left(\begin{array}{c} 0.56,0.36,0.52\text{,}\\ 0.80,0.54,0.34 \end{array}\right)$
$V_{3}$	$\left(\begin{array}{c} 0.12,0.35,0.57\text{,}\\ 0.44,0.51,0.34 \end{array}\right)$	$\left(\begin{array}{c} 0.52,0.34,0.54\text{,}\\ 0.39,0.57,0.36 \end{array}\right)$	$\left(\begin{array}{c} 0.30,0.36,0.53\text{,}\\ 0.39,0.54,0.34 \end{array}\right)$	$\left(\begin{array}{c} 0.50,0.39,0.55\text{,}\\ 0.39,0.51,0.35 \end{array}\right)$
$V_{4}$	$\left(\begin{array}{c} 0.82,0.36,0.59\text{,}\\ 0.17,0.56,0.33 \end{array}\right)$	$\left(\begin{array}{c} 0.97,0.36,0.57\text{,}\\ 0.66,0.57,0.35 \end{array}\right)$	$\left(\begin{array}{c} 0.54,0.36,0.52\text{,}\\ 0.33,0.57,0.34 \end{array}\right)$	$\left(\begin{array}{c} 0.60,0.34,0.55\text{,}\\ 0.34,0.55,0.34 \end{array}\right)$

Step 1**:** Construct the decision matrices (Table 1, Table 2, Table 3, Table 4).

Step 2**:** Construct the normalized decision matrices (Table 5, Table 6, Table 7, Table 8). The closer the distance, the better. As a result, a shorter distance is regarded as advantageous, converting distance to destination into a cost-type criterion. Also, light traffic is preferable, hence less traffic is regarded as advantageous. Thus, traffic condition is a cost-type criterion.

**Table 5:** Normalized decision matrix $R^{\left(1\right)}$.

**Alternatives**	$J_{1}$	$J_{2}$	$J_{3}$	$J_{4}$
$V_{1}$	$\left(\begin{array}{c} 0.95,0.53,0.56\text{,}\\ 0.16,0.45,0.55 \end{array}\right)$	$\left(\begin{array}{c} 0.65,0.65,0.29\text{,}\\ 0.36,0.08,0.28 \end{array}\right)$	$\left(\begin{array}{c} 0.95,0.16,0.95\text{,}\\ 0.05,0.16,0.65 \end{array}\right)$	$\left(\begin{array}{c} 0.96,0.75,0.79\text{,}\\ 0.32,0.73,0.63 \end{array}\right)$
$V_{2}$	$\left(\begin{array}{c} 0.72,0.89,0.66\text{,}\\ 0.45,0.85,0.95 \end{array}\right)$	$\left(\begin{array}{c} 0.26,0.06,0.05\text{,}\\ 0.98,0.06,0.56 \end{array}\right)$	$\left(\begin{array}{c} 0.95,0.45,0.35\text{,}\\ 0.19,0.45,0.55 \end{array}\right)$	$\left(\begin{array}{c} 0.45,0.66,0.96\text{,}\\ 0.64,0.54,0.23 \end{array}\right)$
$V_{3}$	$\left(\begin{array}{c} 0.73,0.26,0.25\text{,}\\ 0.26,0.95,0.65 \end{array}\right)$	$\left(\begin{array}{c} 0.19,0.68,0.98\text{,}\\ 0.79,0.28,0.68 \end{array}\right)$	$\left(\begin{array}{c} 0.15,0.28,0.23\text{,}\\ 0.31,0.25,0.18 \end{array}\right)$	$\left(\begin{array}{c} 0.75,0.46,0.35\text{,}\\ 0.86,0.35,0.83 \end{array}\right)$
$V_{4}$	$\left(\begin{array}{c} 0.25,0.260.65\text{,}\\ 0.25,0.85,0.95 \end{array}\right)$	$\left(\begin{array}{c} 0.15,0.95,0.98\text{,}\\ 0.15,0.75,0.95 \end{array}\right)$	$\left(\begin{array}{c} 0.26,0.83,0.16\text{,}\\ 0.29,0.93,0.86 \end{array}\right)$	$\left(\begin{array}{c} 0.96,0.36,0.13\text{,}\\ 0.85,0.25,0.75 \end{array}\right)$

**Table 6:** Normalized decision matrix $R^{\left(2\right)}$.

**Alternatives**	$J_{1}$	$J_{2}$	$J_{3}$	$J_{4}$
$V_{1}$	$\left(\begin{array}{c} 0.27,0.83,0.85\text{,}\\ 0.25,0.25,0.35 \end{array}\right)$	$\left(\begin{array}{c} 0.23,0.17,0.24\text{,}\\ 0.22,0.94,0.20 \end{array}\right)$	$\left(\begin{array}{c} 0.82,0.83,0.92\text{,}\\ 0.85,0.39,0.80 \end{array}\right)$	$\left(\begin{array}{c} 0.62,0.36,0.35\text{,}\\ 0.75,0.52,0.65 \end{array}\right)$
$V_{2}$	$\left(\begin{array}{c} 0.59,0.32,0.15\text{,}\\ 0.92,0.62,0.12 \end{array}\right)$	$\left(\begin{array}{c} 0.82,0.36,0.95\text{,}\\ 0.47,0.69,0.76 \end{array}\right)$	$\left(\begin{array}{c} 0.29,0.61,0.56\text{,}\\ 0.20,0.86,0.25 \end{array}\right)$	$\left(\begin{array}{c} 0.38,0.65,0.72\text{,}\\ 0.82,0.25,0.32 \end{array}\right)$
$V_{3}$	$\left(\begin{array}{c} 0.59,0.62,0.66\text{,}\\ 0.94,0.35,0.26 \end{array}\right)$	$\left(\begin{array}{c} 0.27,0.73,0.57\text{,}\\ 0.22,0.69,0.73 \end{array}\right)$	$\left(\begin{array}{c} 0.66,0.37,0.35\text{,}\\ 0.26,0.86,0.96 \end{array}\right)$	$\left(\begin{array}{c} 0.79,0.23,0.55\text{,}\\ 0.96,0.86,0.29 \end{array}\right)$
$V_{4}$	$\left(\begin{array}{c} 0.99,0.45,0.06\text{,}\\ 0.97,0.57,0.56 \end{array}\right)$	$\left(\begin{array}{c} 0.27,0.86,0.27\text{,}\\ 0.53,0.26,0.92 \end{array}\right)$	$\left(\begin{array}{c} 0.88,0.65,0.23\text{,}\\ 0.12,0.55,0.49 \end{array}\right)$	$\left(\begin{array}{c} 0.69,0.67,0.59\text{,}\\ 0.82,0.63,0.31 \end{array}\right)$

**Table 7:** Normalized decision matrix $R^{\left(3\right)}$.

**Alternatives**	$J_{1}$	$J_{2}$	$J_{3}$	$J_{4}$
$V_{1}$	$\left(\begin{array}{c} 0.08,0.96,0.85\\ 0.19,0.65,0.65 \end{array}\right)$	$\left(\begin{array}{c} 0.56,0.10,0.85\text{,}\\ 0.39,0.08,0.48 \end{array}\right)$	$\left(\begin{array}{c} 0.54,0.89,0.32\text{,}\\ 0.89,0.56,0.23 \end{array}\right)$	$\left(\begin{array}{c} 0.89,0.30,0.59\text{,}\\ 0.25,0.50,0.20 \end{array}\right)$
$V_{2}$	$\left(\begin{array}{c} 0.89,0.21,0.78\text{,}\\ 0.45,0.98,0.12 \end{array}\right)$	$\left(\begin{array}{c} 0.52,0.29,0.48\text{,}\\ 0.35,0.78,0.68 \end{array}\right)$	$\left(\begin{array}{c} 0.36,0.74,0.56\text{,}\\ 0.23,0.89,0.25 \end{array}\right)$	$\left(\begin{array}{c} 0.65,0.30,0.58\text{,}\\ 0.39,0.51,0.70 \end{array}\right)$
$V_{3}$	$\left(\begin{array}{c} 0.78,0.85,0.41\text{,}\\ 0.69,0.02,0.0.01 \end{array}\right)$	$\left(\begin{array}{c} 0.57,0.69,0.28\text{,}\\ 0.32,0.85,0.69 \end{array}\right)$	$\left(\begin{array}{c} 0.78,0.63,0.74\text{,}\\ 0.23,0.65,0.25 \end{array}\right)$	$\left(\begin{array}{c} 0.23,0.30,0.90\text{,}\\ 0.39,0.50,0.39 \end{array}\right)$
$V_{4}$	$\left(\begin{array}{c} 0.56,0.30,0.78\text{,}\\ 0.11,0.09,0.03 \end{array}\right)$	$\left(\begin{array}{c} 0.58,0.69,0.62\text{,}\\ 0.35,0.85,0.96 \end{array}\right)$	$\left(\begin{array}{c} 0.32,0.56,0.23\text{,}\\ 0.25,0.58,0.52 \end{array}\right)$	$\left(\begin{array}{c} 0.32,0.30,0.54\text{,}\\ 0.56,0.85,0.85 \end{array}\right)$

**Table 8:** Normalized decision matrix $R^{\left(4\right)}$.

**Alternatives**	$J_{1}$	$J_{2}$	$J_{3}$	$J_{4}$
$V_{1}$	$\left(\begin{array}{c} 0.53,0.91,0.84\text{,}\\ 0.35,0.23,0.45 \end{array}\right)$	$\left(\begin{array}{c} 0.53,0.37,0.54\text{,}\\ 0.37,0.52,0.39 \end{array}\right)$	$\left(\begin{array}{c} 0.57,0.38,0.55\text{,}\\ 0.39,0.53,0.37 \end{array}\right)$	$\left(\begin{array}{c} 0.56,0.31,0.56\text{,}\\ 0.34,0.53,0.34 \end{array}\right)$
$V_{2}$	$\left(\begin{array}{c} 0.54,0.25,0.91\text{,}\\ 0.31,0.72,0.85 \end{array}\right)$	$\left(\begin{array}{c} 0.54,0.36,0.56\text{,}\\ 0.36,0.57,0.36 \end{array}\right)$	$\left(\begin{array}{c} 0.60,0.36,0.56\text{,}\\ 0.80,0.54,0.36 \end{array}\right)$	$\left(\begin{array}{c} 0.56,0.36,0.52\text{,}\\ 0.80,0.54,0.34 \end{array}\right)$
$V_{3}$	$\left(\begin{array}{c} 0.57,0.35,0.12\text{,}\\ 0.34,0.51,0.44 \end{array}\right)$	$\left(\begin{array}{c} 0.54,0.34,0.52\text{,}\\ 0.36,0.57,0.39 \end{array}\right)$	$\left(\begin{array}{c} 0.30,0.36,0.53\text{,}\\ 0.39,0.54,0.34 \end{array}\right)$	$\left(\begin{array}{c} 0.50,0.39,0.55\text{,}\\ 0.39,0.51,0.35 \end{array}\right)$
$V_{4}$	$\left(\begin{array}{c} 0.59,0.36,0.82\text{,}\\ 0.33,0.56,0.17 \end{array}\right)$	$\left(\begin{array}{c} 0.57,0.36,0.97\text{,}\\ 0.35,0.57,0.66 \end{array}\right)$	$\left(\begin{array}{c} 0.54,0.36,0.52\text{,}\\ 0.33,0.57,0.34 \end{array}\right)$	$\left(\begin{array}{c} 0.60,0.34,0.55\text{,}\\ 0.34,0.55,0.34 \end{array}\right)$

Step 3**:** Utilize the q-SFREHWA operator as shown in Table 9, where $\omega ={\left(0.3,0.1,0.4,0.2\right)}^{T}$. Table 9 represents the collective normalized decision matrix R.

**Table 9:** Collective normalized decision matrix R.

**Alternatives**	$J_{1}$	$J_{2}$	$J_{3}$	$J_{4}$
$V_{1}$	$\left(\begin{array}{c} 0.5683,0.6951,0.4984\text{,}\\ 0.3245,0.2582,0.3825 \end{array}\right)$	$\left(\begin{array}{c} 0.5693,0.4767,0.2364\text{,}\\ 0.3925,0.5897,0.2469 \end{array}\right)$	$\left(\begin{array}{c} 0.4257,0.3748,0.4595\text{,}\\ 0.3452,0.5748,0.2467 \end{array}\right)$	$\left(\begin{array}{c} 0.8596,0.2431,0.4556\text{,}\\ 0.3744,0.5493,0.1434 \end{array}\right)$
$V_{2}$	$\left(\begin{array}{c} 0.2534,0.4535,0.4121\text{,}\\ 0.8591,0.7845,0.8746 \end{array}\right)$	$\left(\begin{array}{c} 0.2464,0.7456,0.2796\text{,}\\ 0.7456,0.8467,0.2466 \end{array}\right)$	$\left(\begin{array}{c} 0.6278,0.3485,0.4526\text{,}\\ 0.8289,0.5749,0.2486 \end{array}\right)$	$\left(\begin{array}{c} 0.9656,0.4566,0.562\text{,}\\ 0.4750,0.5256,0.2454 \end{array}\right)$
$V_{3}$	$\left(\begin{array}{c} 0.5364,0.4235,0.1498\text{,}\\ 0.6544,0.5236,0.9464 \end{array}\right)$	$\left(\begin{array}{c} 0.8464,0.4564,0.9862\text{,}\\ 0.3974,0.4967,0.9974 \end{array}\right)$	$\left(\begin{array}{c} 0.2890,0.2466,0.2893\text{,}\\ 0.3127,0.5746,0.3748 \end{array}\right)$	$\left(\begin{array}{c} 0.7455,0.9749,0.4756\text{,}\\ 0.3246,0.7851,0.6589 \end{array}\right)$
$V_{4}$	$\left(\begin{array}{c} 0.2439,0.7456,0.2672\text{,}\\ 0.7453,0.9426,0.4257 \end{array}\right)$	$\left(\begin{array}{c} 0.3467,0.4526,0.3567\text{,}\\ 0.3428,0.3647,0.6256 \end{array}\right)$	$\left(\begin{array}{c} 0.1984,0.4756,0.4522\text{,}\\ 0.2463,0.7467,0.2894 \end{array}\right)$	$\left(\begin{array}{c} 0.4785,0.2563,0.6425\text{,}\\ 0.3254,0.2659,0.4257 \end{array}\right)$

**Step 4.** Utilize ${\bar{A}}_{ij}=\sigma {\;\bar{\lambda }}_{i}A_{ij}$, where $\bar{\lambda }={\left(0.1103,0.3423,0.1322,0.4152\right)}^{T}$.

$${\bar{A}}_{11}=\left(0.6548,0.6245,0.6578,0.2567,0.6954,0.1345\right);\;{\bar{A}}_{12}=\left(0.3259,0.2145,0.9846,0.4175,0.2456,0.2456\right)$$

$${\bar{A}}_{13}=\left(0.2563,0.9864,0.4152,0.7425,0.1245,0.7425\right);\;{\bar{A}}_{14}=\left(0.4257,0.2453,0.9452,0.1856,0.9654,0.3745\right)$$

$${\bar{A}}_{21}=\left(0.2436,0.7485,0.2136,0.7851,0.2986,0.4752\right);\;{\bar{A}}_{22}=\left(0.7843,0.2785,0.2986,0.4726,0.2789,0.3674\right)$$

$${\bar{A}}_{23}=\left(0.7245,0.9875,0.2568,0.4728,0.7496,0.4276\right);\;{\bar{A}}_{24}=\left(0.6987,0.9214,0.2456,0.2789,0.9564,0.2475\right)$$

$${\bar{A}}_{31}=\left(0.2456,02789,0.2456,0.2789,0.2452,0.7895\right);\;{\bar{A}}_{32}=\left(0.4435,0.7452,0.1598,0.7423,0.2456,0.7459\right)$$

$${\bar{A}}_{33}=\left(0.2452,0.2879,0.2475,0.9854,0.2456,0.2452\right);\;{\bar{A}}_{34}=\left(0.9489,0.4257,0.8452,0.8496,0.6954,0.1345\right)$$

$${\bar{A}}_{41}=\left(0.2452,0.7425,0.2563,0.3452,0.2469,0.2856\right);\;{\bar{A}}_{42}=\left(0.4752,0.4156,0.2496,0.4196,0.7425,0.4526\right)$$

$${\bar{A}}_{43}=\left(0.4256,0.9874,0.8123,0.6987,0.2496,0.1965\right);\;{\bar{A}}_{44}=\left(0.45248,0.4278,0.3975,0.9746,0.6452,0.2453\right)$$

**Step 5.** Utilize the q-SFREHWA operator to derive the overall preference values. Table 10 represents the overall preference values.

**Table 10:** Overall preferences values.

**Alternatives**	$J_{1}$	$J_{2}$	$J_{3}$	$J_{4}$
$V_{1}$	$\left(\begin{array}{c} 0.5683,0.6951,0.4984\text{,}\\ 0.3245,0.2582,0.3825 \end{array}\right)$	$\left(\begin{array}{c} 0.5693,0.4767,0.2364\text{,}\\ 0.3925,0.5897,0.2469 \end{array}\right)$	$\left(\begin{array}{c} 0.4257,0.3748,0.4595\text{,}\\ 0.3452,0.5748,0.2467 \end{array}\right)$	$\left(\begin{array}{c} 0.8596,0.2431,0.4556\text{,}\\ 0.3744,0.5493,0.1434 \end{array}\right)$
$V_{2}$	$\left(\begin{array}{c} 0.2534,0.4535,0.4121\text{,}\\ 0.8591,0.7845,0.8746 \end{array}\right)$	$\left(\begin{array}{c} 0.2464,0.7456,0.2796\text{,}\\ 0.7456,0.8467,0.2466 \end{array}\right)$	$\left(\begin{array}{c} 0.6278,0.3485,0.4526\text{,}\\ 0.8289,0.5749,0.2486 \end{array}\right)$	$\left(\begin{array}{c} 0.9656,0.4566,0.562\text{,}\\ 0.4750,0.5256,0.2454 \end{array}\right)$
$V_{3}$	$\left(\begin{array}{c} 0.5364,0.4235,0.1498\text{,}\\ 0.6544,0.5236,0.9464 \end{array}\right)$	$\left(\begin{array}{c} 0.8464,0.4564,0.9862\text{,}\\ 0.3974,0.4967,0.9974 \end{array}\right)$	$\left(\begin{array}{c} 0.2890,0.2466,0.2893\text{,}\\ 0.3127,0.5746,0.3748 \end{array}\right)$	$\left(\begin{array}{c} 0.7455,0.9749,0.4756\text{,}\\ 0.3246,0.7851,0.6589 \end{array}\right)$
$V_{4}$	$\left(\begin{array}{c} 0.2439,0.7456,0.2672\text{,}\\ 0.7453,0.9426,0.4257 \end{array}\right)$	$\left(\begin{array}{c} 0.3467,0.4526,0.3567\text{,}\\ 0.3428,0.3647,0.6256 \end{array}\right)$	$\left(\begin{array}{c} 0.1984,0.4756,0.4522\text{,}\\ 0.2463,0.7467,0.2894 \end{array}\right)$	$\left(\begin{array}{c} 0.4785,0.2563,0.6425\text{,}\\ 0.3254,0.2659,0.4257 \end{array}\right)$

**Step 6.** Utilize the q-SFREHWA operator to derive the overall preference values.

Calculate the overall preference values $B_{i}$ (i=1,2,3,4) for the alternative $V_{i}\;\left(i=1,2,3,4\right)$ using the given data and the q−SFREHWA operator as shown below:

$$B_{1}=\left(\begin{array}{c} 0.2415,0.3642,0.2896,\\ 0.4952,0.8415,0.5289 \end{array}\right),B_{2}=\left(\begin{array}{c} 0.3459,0.2789,0.2452,\\ 0.8975,0.3896,0.9428 \end{array}\right),B_{3}=\left(\begin{array}{c} 0.9456,0.2415,0.9854,\\ 0.2652,0.7858,0.2415 \end{array}\right)\;\text{and}\;B_{4}=\left(\begin{array}{c} 0.4856,0.2459,0.2457,\\ 0.3452,0.6854,0.6985 \end{array}\right)\text{.}$$

By applying Equation (9), we obtain the following score values for the alternatives: $Sco\left(B_{1}\right)=0.4397\text{,}$ $Sco\left(B_{2}\right)=0.6102,Sco\left(B_{3}\right)=0.4646$ and $Sco\left(\;B_{4}\right)=0.4877$. The alternatives are arranged in the following order based on the computed score values: *V* $V_{2}>V_{4}>V_{3}>V_{1}$. This score identifies Route B as the best option. Table 11 displays the score values and ranks obtained with the q-SFREWA, q-SFREOWA, and q-SFRHWA operators. This table serves as a comprehensive reference, describing how each operator influences the ranking of possibilities, assisting in the selection of the optimum route for the autonomous vehicle navigation system.

**Table 11:** The scores are calculated using the q-SFREWA, q-SFREOWA, and q-SFRHWA operators, which represent their relative contributions to alternative rankings.

Operators	Score values				Ranking
	$V_{1}$	$V_{2}$	$V_{3}$	$V_{4}$	
q−SFREWA	0.4546	0.8474	0.6453	0.8285	$V_{2}>V_{4}>V_{3}>V_{1}$
q−SFREOWA	0.4895	0.9529	0.6752	0.8457	$V_{2}>V_{4}>V_{3}>V_{1}$
q−SFRHWA	0.4397	0.6102	0.4646	0.4877	$V_{2}>V_{4}>V_{3}>V_{1}$

**Fig. 9:**
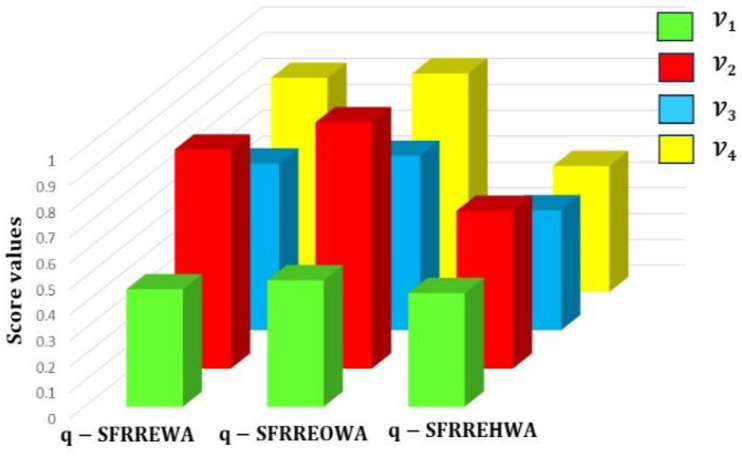
Graphical representation of score values of q-SFREWA, q-SFREOWA, and q-SFRHWA.

The graphical representation of score values is shown in Fig. 9.

This work also explores the practical applications of the proposed q-SFR Einstein operators, particularly when decision-makers seek to tailor their aggregation methods according to human preferences. Table 11 presents the outcomes achieved using these operators, illustrating how decision-makers can refine their assessments by integrating assigned values with expert insights. This discussion underscores that the suggested aggregation operators offer decision-makers a more adaptable framework for identifying optimal choices. Compared to traditional methods, these operators significantly enhance flexibility, allowing for a broader range of decision-making scenarios. Their adaptability enables decision-makers to customize aggregation strategies to specific requirements and preferences, thereby facilitating a more personalized and efficient decision-making process. By integrating both assigned values and expert opinions, the proposed operators provide a comprehensive approach to decision-making. This dual consideration ensures that decisions are well-rounded, incorporating both quantitative data and qualitative perspectives. Designed for versatility, the proposed operators are applicable across diverse decision domains, enhancing their practical utility. Their adaptability contributes to the robustness and reliability of decision-making processes, ensuring consistency and stability in various contexts and under varying conditions. The findings suggest that the proposed aggregation operators empower decision-makers to make informed and adaptive choices. Their flexibility and inclusivity support decision-makers in aligning decisions with specific needs and preferences, while their generalizability enhances effectiveness across different decision-making scenarios. By offering an adaptive and comprehensive framework, the q-SFR Einstein operators enable decision-makers to navigate complex challenges with confidence and precision, ultimately facilitating optimal outcomes in diverse settings.

### 4.2 Consequence of q on ranking order and score values

To justify the restriction requirement $\left(0\leq {{\underline{\zeta }}_{A}}^{q}\left(p\right)+{{\underline{\eta }}_{A}}^{q}\left(p\right)+{{\underline{\xi }}_{A}}^{q}\left(p\right)\leq 1\right)$ and $\left(0\leq {{\bar{\zeta }}_{A}}^{q}\left(p\right)+{{\bar{\eta }}_{A}}^{q}\left(p\right)+{{\bar{\xi }}_{A}}^{q}\left(p\right)\leq 1\right)$, a decision-maker must determine the smallest integer parameter q by examining the attribute values. For instance, when evaluating an alternative with attribute values (0.8,0.7,0.9,0.9,0.8,0.7), q should be chosen as 3 or 4 since both configurations meet the criterion. To thoroughly assess the impact of parameter q on the tentative outcomes, we tested several values of q using the novel approach. Table 12 presents the results of these variations, indicating that $V_{2}$ consistently ranks at the top, followed by ${\;V}_{4}$, $V_{3}\text{,}$ and finally $V_{1}$. This consistent ranking underscores the significance of the best alternative and the stability of the ranking order. When analyzing the effects of different q values, we observed that the ranking of alternatives remained stable. Table 12 specifically illustrates that the parameter q has a significant impact on the ranking order and score values. By ensuring that the chosen q values meet the necessary constraints, decision-makers can maintain the stability and reliability of their rankings. The findings confirm that the method is robust across various q values, with consistent rankings that support the selection of the best alternatives. This robustness emphasizes the method's applicability and reliability in diverse decision-making scenarios. The ranking order of alternatives remains stable across different values of q. The method ensures reliable rankings by selecting appropriate q values that meet necessary constraints. The approach is robust and adaptable to various decision-making scenarios, maintaining consistent and accurate rankings. The method's versatility and reliability extend its applicability across a broad spectrum of decision-making scenarios, thereby enhancing its practical utility. These attributes underscore the effectiveness and dependability of the proposed approach in assessing and prioritizing alternatives based on the parameter q.

**Table 12:** Ranking of alternatives based on their respective q parameter values.

q	$V_{1}$	$V_{2}$	$V_{3}$	$V_{4}$
1	0.4125	0.5923	0.4419	0.4651
2	0.4179	0.5987	0.4464	0.4696
3	0.4397	0.6102	0.4646	0.4877
4	0.4430	0.6175	0.4687	0.4899
**5**	0.4465	0.6323	0.4735	0.4913
**6**	0.4498	0.6366	0.4789	0.4952
**7**	0.4528	0.6434	0.4844	0.5043
**8**	0.4582	0.6476	0.4874	0.5092
**9**	0.4621	0.6540	0.4913	0.5139
**10**	0.4682	0.6595	0.4980	0.5193

**Fig. 10:**
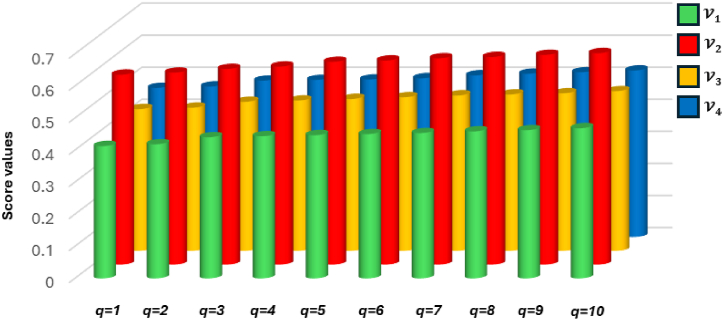
Ranking of alternatives with different q values.

Table 12 provides a detailed ranking of alternatives based on their respective q-parameter values, illustrating the steadfastness and reliability of the ranking order across a spectrum of q-values. The data shows a clear pattern: as the q values increase from 1 to 10, the alternatives values consistently increase, showcasing a positive relationship between the q parameter and the values of these alternatives. Each alternative value increases steadily, reflecting a positive relationship between *q* and the values of these alternatives. The minor fluctuations do not significantly impact the overall increasing trend. The graphical representation of different q values is shown in Fig. 10.

The method's versatility and reliability extend its applicability across a broad spectrum of decision-making scenarios, thereby enhancing its practical utility. These attributes underscore the effectiveness and dependability of the proposed approach in assessing and prioritizing alternatives based on the parameter q.

### 4.3 Test of validity

To demonstrate the flexibility and robustness of the recommended approach in a variety of circumstances, we use the assessment procedures proposed by Wang and Triantaphyllou [57], as follows:

Step 1**:** The initial validation stage involves substituting the rating values of suboptimal alternatives with those of even lower quality. This substitution should not affect the determination of the optimal alternative. Specifically, the choice that appears to be the best should not change, as long as the relative weights of the criterion stay stable. This stage guarantees that the model continues to identify the best alternative, independent of changes in the lower-ranked alternatives.

Step 2**:** The second validation step requires that the procedure follows the principle of transitivity. Transitivity in decision-making means that if alternative A is preferred over alternative B and alternative B is preferred over alternative C, then alternative A should also be preferred over alternative C. This step verifies that the model maintains logical consistency in its ranking process, ensuring that the preference order is coherent and rational.

Step 3**:** The third validation step tests the model's consistency when a given decision-making problem is divided into smaller sub-problems. When applying the same decision-making process to these smaller problems, the initial ranking of the alternatives should remain unchanged. This step ensures that the model's rankings are stable and reliable, even when the problem is broken down into simpler, more manageable parts.

By adhering to these evaluation protocols, the proposed technique demonstrates its validity and reliability in various decision-making scenarios. These steps confirm that the model.

i. Maintains the selection of the highest-rated alternative even when less-than-ideal ratings are replaced.
ii. Follows the transitivity principle to maintain a coherent and rational preference order.
iii. Preserves the initial ranking of alternatives, even when the problem is subdivided into smaller segments.

These tests of validity provide strong evidence that the proposed technique is both adaptable and robust, capable of delivering consistent and reliable results across different settings and scenarios.

Test of validity utilizing criteria 1: The alternatives ranked according to our proposed technique are $V_{2}>V_{4}>V_{3}>V_{1}$. To assess the method's stability under test criterion 1, we replaced the non-optimal alternative $V_{1}$ with the lowest-ranked alternative $V_{1}^{*}$ and compared the rankings.

The rating values used for $F_{2}^{*}$ were (0.59,0.26,0.47,0.36,0.74,0.25), (0.25,0.47,0.25,0.89,0.74,0.69), (0.85,0.25,0.63,0.96,0.25,0.25) and (0.54,0.84,0.36,0.25,0.74,0.96). Using our suggested methodology, the scores for the alternatives are calculated as follows:

$Sco\left(F_{1}^{*}\right)=0.2390\text{,}$$Sco\left(F_{2}\right)=0.9613$, $Sco\left(F_{3}\right)=0.4390$ and $Sco\left(F_{4}\right)=0.7245$. As a result, the ranking order is $V_{2}>V_{4}>V_{3}>V_{1}^{*}$, with the best alternative remaining the same as in the initial suggested approach. Thus, the findings consistently support test criteria 1, demonstrating that replacing a less-than-ideal alternative with an inferior one does not affect the correspondence of the best alternative.

Test of validity employing criteria 2 and 3: To further assess the validity of our decision-making process based on criteria 2 and 3, we examined fragmented decision-making subcases. These subcases included specific sets of alternatives: $\left\{V_{1},V_{2},V_{3}\right\}$, $\left\{V_{2},V_{3},V_{4}\right\}$ and $\left\{V_{1},V_{3},V_{4}\right\}$. Through our suggested procedures, we ranked these subcases as follows:

For the subcase $\left\{V_{1},V_{2},V_{3}\right\}$, the ranking order was $V_{2}>V_{3}>V_{1}$.
For the subcase $\left\{V_{2},V_{3},V_{4}\right\}$, the ranking order was $V_{2}>V_{4}>V_{3}$.
For the subcase $\left\{V_{1},V_{3},V_{4}\right\}$, the ranking order was $V_{4}>V_{3}>V_{1}$.

By joining all these conclusions, the complete position seems as $V_{2}>V_{4}>V_{3}>V_{1}$. This mirrors the results obtained from the initial decision-making method. This consistency demonstrates that our proposed approach satisfies requirements 2 and 3, ensuring logical consistency (transitivity) and stability when the problem is decomposed into smaller components.

## 5 Managerial implications

The novel q-SFR Einstein operators have significant managerial implications, aiding managers and decision-makers in making strategic decisions and achieving robust, reliable outcomes. This paradigm displays great adaptability across several industries and is useful in a variety of decision-making scenarios. Managers in the automotive industry can leverage this model to evaluate and select the most advantageous navigation system for automatic cars. The model assists in considering various factors, ensuring a comprehensive assessment that identifies the best technology suited to their needs. In industrial contexts, the model assists managers in selecting the most suitable maintenance techniques for their equipment or systems. By evaluating various maintenance procedures, managers can enhance operational efficiency and prolong asset lifespan. Furthermore, the approach is useful in evaluating robots employed in industrial situations. It assists managers in evaluating the utility and application of various robotic systems, allowing them to make better decisions that improve production and operational efficiency. The model may also be used to choose material handling equipment. Managers may use the framework to make educated decisions on the most effective and productive equipment for their specific needs, resulting in smooth and efficient material handling operations. While the q-SFR Einstein operators provide a scientific and systematic approach to decision-making, it is essential to recognize that the process within this framework is substantially influenced by the preferences and judgments of the experts and individuals involved. The model offers a structured method, but the final decisions and rankings are shaped by the subjective assessments of the decision-makers. Therefore, the inclusion of experts and stakeholders is crucial to ensuring the accuracy and relevance of the findings. Two critical analyses are performed to validate and strengthen the obtained results.

Comparative analysis is a useful tool for decision-makers to review and compare rankings and results across several options, each evaluated using its own set of criteria. It raises awareness of trade-offs and facilitates better-informed decision-making by stressing the advantages and disadvantages of each option. Sensitivity analysis reveals important information on the stability and sensitivity of the results. Decision-makers can assess the multitude of factors influencing their decisions, thereby enhancing their capacity to make adaptable judgments in evolving contexts. Integrating this study into the decision-making process enables managers to enhance the reliability and confidence in their strategic decisions. The q-SFR Einstein operators, supported by comparative and sensitivity analyses, provide a comprehensive framework that equips managers across various sectors and applications with the tools needed to make informed and resilient choices.

### 5.1 Comparative analysis

Table 13 presents a comparative evaluation of rankings achieved through the utilization of the q-SFR Einstein operators in contrast to four other methods for multi-criteria decision-making (MCDM). A review of recent research publications reveals diverse contributions to fuzzy set theory and decision-making processes. Khan et al. [58] propose a spherical fuzzy rough EDAS method for cache replacement policies that use Einstein aggregation operators, highlighting the necessity of innovative aggregation methodologies in enhancing caching strategies. On the other hand, Ashraf et al. [59] study multi-attribute decision-making settings, highlighting the flexibility and utility of spherical fuzzy sets. Furthermore, Ashraf, Abdullah, and Mahmood [60] widen the study of group decision-making challenges by presenting spherical fuzzy Dombi aggregation operators as a novel method. An examination of recent research articles indicates diverse contributions to fuzzy set theory and decision-making processes. Khan et al. [58] introduce a spherical fuzzy rough EDAS method for cache replacement policies that employ Einstein aggregation operators, emphasizing the need for novel aggregation approaches in improving caching strategies. In contrast, Ashraf et al. [59] examine multi-attribute decision-making scenarios, emphasizing the adaptability and usefulness of spherical fuzzy sets. Furthermore, Ashraf, Abdullah, and Mahmood [60] broadened their investigation into group decision-making issues by using spherical fuzzy Dombi aggregation operators as a new strategy.

**Table 13:** Comparative analysis of rankings across differing methodologies.

Aggregation Operators	References	Score Values	Ranking
SFREWG	[58]	(0.4570,0.6869,0.3054,0.6041)	$V_{2}>V_{4}>V_{3}>V_{1}$
SFREOWG	[58]	(0.5000,0.5192,0.3959,0.5087)	$V_{2}>V_{4}>V_{3}>V_{1}$
SFREHG	[58]	(0.2878,0.5000,0.2218,0.3724)	$V_{2}>V_{4}>V_{3}>V_{1}$
SFWG	[59]	(0.5397,0.6910,0.3538,0.5552)	$V_{2}>V_{4}>V_{3}>V_{1}$
SFOWG	[59]	0.4684,0.5817,0.3959,0.5087)	$V_{2}>V_{4}>V_{3}>V_{1}$
SFHG	[59]	(0.4570,0.5129,0.2218,0.3724)	$V_{2}>V_{4}>V_{3}>V_{1}$
SFDWG	[60]	(0.4570,0.6720,0.3054,0.6041)	$V_{2}>V_{4}>V_{3}>V_{1}$
SFDOWG	[60]	(0.4155,0.4622,0.3183,0.4416)	$V_{2}>V_{4}>V_{3}>V_{1}$
SFDHWG	[60]	(0.1678,0.2679,0.1270,0.2312)	$V_{2}>V_{4}>V_{3}>V_{1}$
q-SFREWA	[This Paper]	(0.6546,0.4574,0.9453,0.8785)	$V_{2}>V_{4}>V_{3}>V_{1}$
q-SFREOWA	[This Paper]	(0.4895,0.3529,0.6752,0.5457)	$V_{2}>V_{4}>V_{3}>V_{1}$
q-SFREHWA	[This Paper]	(0.4397,0.6102,0.4646,0.4877)	$V_{2}>V_{4}>V_{3}>V_{1}$

**Fig. 11:**
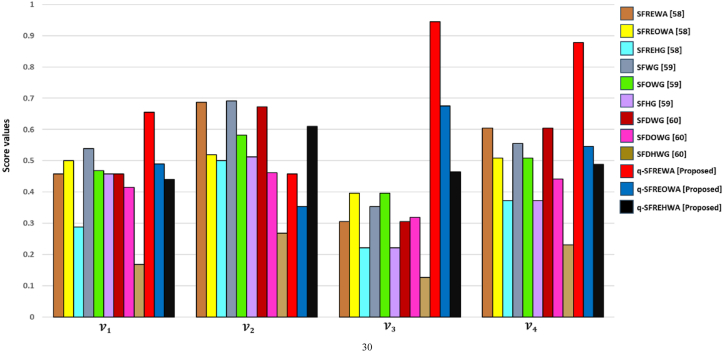
Graphical representation of ranking comparison of different operators with the proposed operators.

Fig. 11 depicts the ranking comparison between various operators and the proposed operators.

Amidst the propositions, calculations, and practical applications outlined above, the unique benefits of adopting q-SFRSs become evident, paving the way for enhanced decision-making.

1. Traditional fuzzy sets and intuitionistic fuzzy sets, while useful, sometimes fail to capture all important information in certain cases. Membership and non-membership degrees may limit decision-makers' ability to explain nuanced perspectives.
2. To address these restrictions, Yager developed Pythagorean fuzzy sets, which expanded the extent of representation and enabled a wider range of applications. However, in the context of uncertain information, such as voting systems, the rigidity of picture fuzzy sets may be limiting, particularly in terms of decision-maker flexibility.
4. The spherical fuzzy sets, offer a solution that gracefully navigates diverse information sets without exceeding the bounds of unity. This adaptability empowers decision-makers to allocate membership values according to their unique preferences.
5. The incorporation of q-spherical fuzzy rough sets, along with associated algorithms, presents a versatile framework with far-reaching implications across various decision-making processes.
6. Furthermore, the proposed aggregation operators excel in handling imprecise information, offering a level of reliability that surpasses existing methodologies.
7. The applicability of q-SFRSs spans a multitude of domains, including stock investment analysis, airline service quality evaluation, investment banking authority selection, and electronic learning factor assessment, underscoring their broad utility and relevance.
8. By embracing the advantages inherent in q-spherical fuzzy rough sets, decision-makers are better equipped to traverse the intricate landscapes of decision-making with heightened confidence and precision.
9. Regarding the specific concerns about the limitations of picture fuzzy rough sets (PFSRS) and spherical fuzzy rough sets, it's important to acknowledge that they are constrained by specific numerical bounds within their approximations. In contrast, q-spherical fuzzy rough sets offer a broader scope of representation, allowing for a more nuanced handling of information sets. This distinction underscores the versatility and potential superiority of q-SFRS in handling complex decision-making scenarios.
10. The q-SFRSs are more general than other algebraic structures because they incorporate lower and upper approximations with membership, neutral, and non-membership degrees. The inclusion of a q-parameter further enhances their robustness compared to PFSs and SFSs. This parameter allows for more flexible and nuanced modeling of uncertainty. Additionally, the merging of Einstein operators strengthens their robustness.

**Fig. 12:**
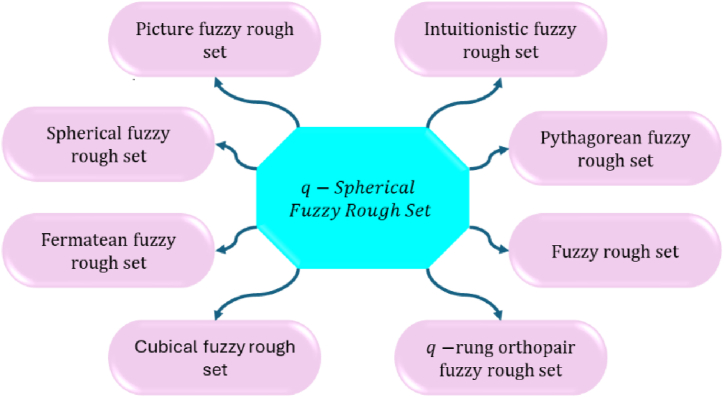
Some particular cases of different algebraic structures compared to q-spherical fuzzy rough sets.

Fig. 12 represents some particular cases of q-SFRSs.

### 5.2 Sensitivity analysis

In this study, the developed model undergoes rigorous testing through two distinct sensitivity analyses, one of which examines variations in criteria and decision-making weights and their impact on final rankings.

1. The criteria weight analysis explores the effect of varying the weights of different criteria on the overall ranking of alternatives. It ensures that the model's outcomes are robust and not overly sensitive to changes in the criteria's importance.
2. The decision-maker weight analysis examines the impact of different weight distributions assigned to decision-makers on the final rankings. By evaluating various scenarios, it ensures that the model remains consistent and reliable across different decision-weighting configurations.

**Fig. 13:**
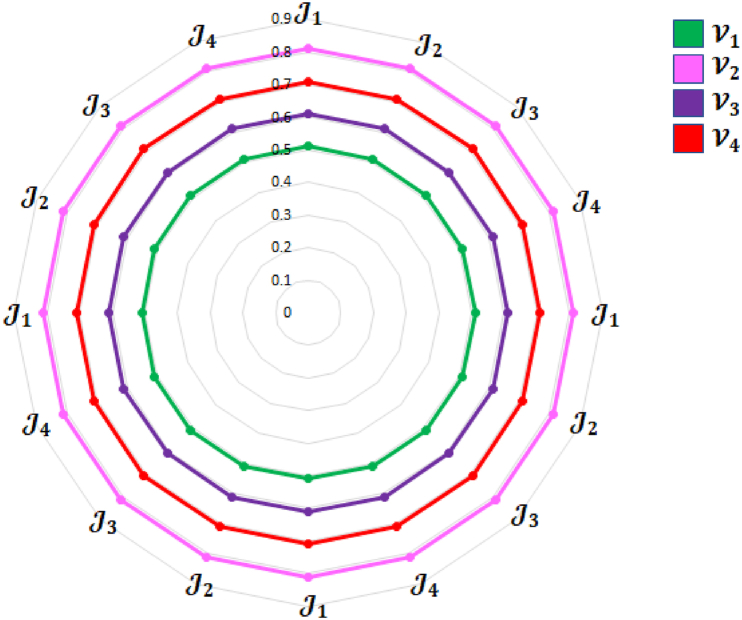
Alternative rankings based on different criteria weight variations.

**Fig. 14:**
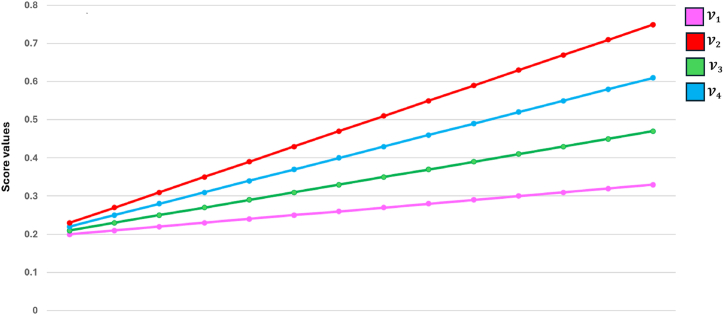
Alternative rankings based on changes in decision-maker weights.

By incorporating these analyses, managers can have greater confidence in the robustness and reliability of the decision-making process facilitated by the q-SFR Einstein operators, making it a valuable tool in strategic decision-making across various sectors. In conclusion, the q-SFR Einstein operators provide a flexible and reliable decision-making framework that can be applied across numerous industries, from automotive to industrial environments. Their ability to incorporate expert judgments and adapt to varying criteria and decision-maker preferences ensures that the results are both credible and applicable to real-world scenarios. This innovative approach helps managers make informed, strategic decisions that enhance operational efficiency, productivity, and overall success. The initial sensitivity analysis conducted a temporal examination to assess how varying priority levels assigned to reference criteria—high, equal, and low—affect overall ranking outcomes. This strategy requires running the model for each criteria individually and applying appropriate reference weights. Fig. 13 displays the results from twenty different scenarios. Surprisingly, in every example, option $V_{2}$ ranks first, whereas option $V_{1}$ always ranks last. Despite major changes in criteria weights, the model stays somewhat sensitive to these changes, demonstrating its ability to maintain consistent ranks across priority levels. The second sensitivity analysis adjusts the weights assigned to decision-makers, resulting in four distinct scenarios with varying weight distributions. Fig. 14 illustrates the outcomes of these scenarios. Throughout all configurations, alternative $V_{2}$ consistently emerges as the most preferred option, while alternative $V_{1}$ remains the least favored. Although the rankings of other alternatives fluctuate with different decision-maker weights, the proposed model demonstrates robustness and consistency across a broad spectrum of weight distributions. These sensitivity analyses underscore the model's reliability and robustness. The first analysis confirms that the model is largely insensitive to variations in criteria weights, consistently identifying the top and bottom alternatives. The second analysis demonstrates that the model remains stable and reliable even when the weights assigned to decision-makers are adjusted, reaffirming the preferred and least favored alternatives. This comprehensive verification process ensures that the model can be confidently applied in various decision-making scenarios, providing consistent and dependable results.

### 5.3 Advantages

The suggested approach has several key advantages.

1. By incorporating the parameter q into the aggregation operators, decision-makers benefit from enhanced flexibility. This adaptability enables customization of settings to suit the specific demands and preferences of each decision-making scenario. The technique's flexibility accommodates varying degrees of membership, neutral membership, and non-membership with lower and upper set approximations, making it suitable for diverse real-world applications. This customization ensures that the decision-making process accurately reflects the intricacies and complexities of different scenarios.
2. The parametric structure of the proposed operators enables decision-makers to adjust the influence of membership, neutral membership, and non-membership levels along with lower and upper set approximations. This level of control is critical as it allows decision-makers to tailor the aggregation process to their preferences and the specific details of the scenario at hand. By fine-tuning these parameters, decision-makers can ensure that the aggregation process aligns perfectly with their preferred decision-making approach, leading to more precise and dependable results.
3. The symmetry of the proposed aggregation operators with respect to the parameter ensures that the ranking orders of alternatives remain consistently stable across different parameter values. This consistency is a significant advantage in decision-making, as it mitigates the potential impact of decision-makers' inherent biases, whether pessimism or optimism. The stability of ranking orders across various parameter settings enhances the reliability of the decision-making process, instilling confidence that the results are robust and dependable, independent of subjective inclinations of decision-makers.

Overall, the proposed technique significantly improves the decision-making process by offering enhanced flexibility, precise control, and consistent reliability. By incorporating the parameter q, decision-makers can adapt the process to a wide variety of scenarios, ensuring accurate and trustworthy outcomes. This adaptability and robustness make the technique a valuable tool for effective decision-making across diverse applications.

### 5.4 Limitations

Every research project has inherent limitations, and the approach detailed in this study is no different. The following points outline these constraints.

1. The proposed technique may be restricted in its applicability to specific domains or decision contexts. While it offers significant flexibility and precision, its effectiveness and relevance might be limited outside the contexts for which it was designed. Understanding these boundaries is crucial for determining when and where the recommended strategy can be optimally utilized. Decision-makers should assess the suitability of this approach to the particularities of their domain to ensure it is the best fit for their needs.
2. Like any research method, the proposed approach is built upon certain assumptions and simplifications that facilitate analysis. These assumptions are necessary for creating a manageable and understandable model, but they may not perfectly align with real-world conditions. This misalignment could potentially limit the broader or practical applicability of the results. Decision-makers should be aware of these assumptions and consider their potential impact on the applicability of the findings in more complex or varied real-world scenarios.
3. The validation of the proposed framework is based on a case study involving four alternatives and four criteria. While this case study provides initial evidence of the method's effectiveness, it is important to acknowledge that the framework's scalability and robustness need further testing. Future research should aim to expand the framework to incorporate more alternatives and criteria, thereby evaluating its performance and adaptability in more complex and diverse decision-making environments.
4. The current study explores alternative ranking orders for several values of the parameter q. However, the investigation is not exhaustive, and additional research is necessary to fully understand how varying this parameter impacts the hierarchical order of alternatives. Further studies should focus on exploring a wider range of parameter values to provide a more comprehensive understanding of their effects. This would help in identifying the conditions under which the proposed method performs optimally and ensuring its robustness across different parameter settings.

## 6 Conclusion and recommendation for future work

In this paper, we explored aggregation operators, focusing on new q-SFREWA, q-SFREOWA, and q-SFREHWA operators for q-SFRSs. Clear operational laws are crucial in decision-making, and Einstein operators effectively incorporate experts' preferences. Our goal was to enhance decision-making using these operators, resulting in smoother and more effective processes. We presented and investigated the Einstein sum and Einstein product for q-SFRNs, resulting in numerous q-SFREWA, q-SFREOWA, and q-SFRHWA operators. These operators establish a framework that incorporates decision-makers' preferences. We also explored the relationships between different aggregation operators to gain a deeper understanding of their interactions. To show its practical utility, we suggested a new approach for multiple attribute group decision-making (MAGDM) that successfully solves MCDM. We evaluated our approach using a practical example of selecting a navigation system for automatic cars, performing a comparative study with existing methods, and a sensitivity analysis to confirm its effectiveness. Looking ahead, we plan to extend our framework to address fuzziness and ambiguity in various decision-making parameters, such as design choices, construction options, site selection, and decision-making in a soft-set environment. We will also explore other extensions of fuzzy sets, like quasirung fuzzy sets, which handle uncertainty and ambiguity effectively. Seikh and Mandal [61,62] work on MAGDM with quasirung orthopair fuzzy sets, particularly for electric vehicle charging station site selection, highlighting their potential in complex decisions. Integrating quasirung fuzzy sets into our framework is a promising direction for future research. Additionally, enhancing our literature review, historical background, and future work sections by incorporating recent advancements and seminal works will provide a more comprehensive context for our research. Hussain et al. [63,64] research on decision algorithms for picture fuzzy sets and Aczel Alsina aggregation operators, along with their innovative approach using Schweizer-Sklar aggregation operators for picture fuzzy sets with unknown weights, will significantly expand our study's scope. This integration ensures our work is well-grounded in existing knowledge while pushing the boundaries of current decision-making frameworks.

## Funding

This project is supported by funding from 10.13039/501100002383King Saud University, Riyadh, Saudi Arabia.

## Consent for publication

This manuscript has not been previously published and is not currently under consideration for publication elsewhere.

## Data availability statement

The manuscript does not include any associated data. It exclusively presents written text and does not contain additional data supporting the claims and conclusions presented in the manuscript.

## CRediT authorship contribution statement

**Ahmad Bin Azim:** Conceptualization. **Asad Ali:** Supervision. **Abdul Samad Khan:** Data curation. **Fuad A. Awwad:** Funding acquisition. **Sumbal Ali:** Software. **Emad A.A. Ismail:** Investigation.

## Declaration of competing interest

The authors declare that they have no known competing financial interests or personal relationships that could have appeared to influence the work reported in this paper.

## References

[1] Zadeh L.A. (1965). Fuzzy sets. Inf. Control.

[2] Zadeh L.A. (1975). The concept of a linguistic variable and its application to approximate reasoning—I. Inf. Sci..

[3] Atanassov K.T., Stoeva S. (1986). Intuitionistic fuzzy sets. Fuzzy Set Syst..

[4] Atanassov K.T. (1994). Operators over interval-valued intuitionistic fuzzy sets. Fuzzy Set Syst..

[5] Zadeh L.A. (1973). Outline of a new approach to the analysis of complex systems and decision processes. IEEE Transactions on Systems, Man, and Cybernetics.

[6] Bustince H., Burillo P. (1996). Structures on intuitionistic fuzzy relations. Fuzzy Set Syst..

[7] Deschrijver G., Kerre E.E. (2003). On the relationship between some extensions of fuzzy set theory. Fuzzy Set Syst..

[8] Turksen I.B. (1986). Interval-valued fuzzy sets based on normal forms. Fuzzy Set Syst..

[9] Xu Z., Cai X. (2010). Recent advances in intuitionistic fuzzy information aggregation. Fuzzy Optim. Decis. Making.

[10] Xu Z. (2007). Intuitionistic fuzzy aggregation operators. IEEE Trans. Fuzzy Syst..

[11] Xu Z., Yager R.R. (2006). Some geometric aggregation operators based on intuitionistic fuzzy sets. Int. J. Gen. Syst..

[12] Zeng S., Su W. (2011). Intuitionistic fuzzy ordered weighted distance operator. Knowl. Base Syst..

[13] Yager R.R. (2013). 2013 Joint IFSA World Congress and NAFIPS Annual Meeting (IFSA/NAFIPS).

[14] Yager R.R., Abbasov A.M. (2013). Pythagorean membership grades, complex numbers, and decision making. Int. J. Intell. Syst..

[15] Jun Y.B., Smarandache F., Kim C.S. (2017). Neutrosophic cubic sets. New Math. Nat. Comput..

[16] Smarandache F. (1999). Philosophy.

[17] Ali M., Deli I., Smarandache F. (2016). The theory of neutrosophic cubic sets and their applications in pattern recognition. J. Intell. Fuzzy Syst..

[18] Ye J. (2018). Operations and aggregation method of neutrosophic cubic numbers for multiple attribute decision-making. Soft Comput..

[19] Ajay D., Broumi S., Aldring J. (2020). An MCDM method under neutrosophic cubic fuzzy sets with geometric Bonferroni mean operator. Neutrosophic Sets and Systems.

[20] Cuong B.C., Kreinovich V. (2013). 2013 Third World Congress on Information and Communication Technologies (WICT 2013).

[21] Atta R., Ghanbari M., Elnahry I. (2021). Advanced image steganography based on exploiting modification direction and neutrosophic set. Multimed. Tool. Appl..

[22] Kutlu Gündoğdu F., Kahraman C. (2019). Spherical fuzzy sets and spherical fuzzy TOPSIS method. J. Intell. Fuzzy Syst..

[23] Ashraf S., Abdullah S. (2019). Spherical aggregation operators and their application in multiattribute group decision‐making. Int. J. Intell. Syst..

[24] Ashraf S., Abdullah S., Mahmood T. (2018). GRA method based on spherical linguistic fuzzy Choquet integral environment and its application in multi-attribute decision-making problems. Mathematical Sciences.

[25] Jin Y., Ashraf S., Abdullah S. (2019). Spherical fuzzy logarithmic aggregation operators based on entropy and their application in decision support systems. Entropy.

[26] Rafiq M. (2019). The cosine similarity measures of spherical fuzzy sets and their applications in decision making. J. Intell. Fuzzy Syst..

[27] Ashraf S., Abdullah S., Mahmood T. (2020). Spherical fuzzy Dombi aggregation operators and their application in group decision-making problems. J. Ambient Intell. Hum. Comput..

[28] Kutlu Gündoğdu F., Kahraman C., Karaşan A. (2020). Proceedings of the INFUS 2019 Conference.

[29] Acharjya D., Rathi R. (2022). An integrated fuzzy rough set and real coded genetic algorithm approach for crop identification in smart agriculture. Multimed. Tool. Appl..

[30] Sharaff A., Khaire A.S., Sharma D. (2019). The *2019 International Conference On Intelligent Computing and Control Systems (ICCS)*.

[31] Sharaff A., Verma A., Shrawgi H. (2018). Proceedings of the International Conference on Computing and Communication Systems: I3CS 2016, NEHU.

[32] Gou X., Xu Z., Liao H. (2016). Exponential operations of interval-valued intuitionistic fuzzy numbers. International Journal of Machine Learning and Cybernetics.

[33] Seikh M.R., Mandal U. (2023). Interval-valued Fermatean fuzzy Dombi aggregation operators and SWARA based PROMETHEE II method to bio-medical waste management. Expert Syst. Appl..

[34] Seikh M.R., Chatterjee P. (2024). Determination of best renewable energy sources in India using SWARA-ARAS in confidence level based interval-valued Fermatean fuzzy environment. Appl. Soft Comput..

[35] Mahmood T. (2019). An approach toward decision-making and medical diagnosis problems using the concept of spherical fuzzy sets. Neural Comput. Appl..

[36] Wang H. (2021). T-spherical fuzzy rough interactive power Heronian mean aggregation operators for multiple attribute group decision-making. Symmetry.

[37] Wang H. (2024). A novel CODAS approach based on Heronian Minkowski distance operator for T-spherical fuzzy multiple attribute group decision-making. Expert Syst. Appl..

[38] Ali A. (2022). Heronian mean operators based multi-attribute decision making algorithm using T-spherical fuzzy information. Journal of Innovative Research in Mathematical and Computational Sciences.

[39] Jaleel A. (2022). WASPAS technique utilized for agricultural robotics system based on Dombi aggregation operators under bipolar complex fuzzy soft information. Journal of Innovative Research in Mathematical and Computational Sciences.

[40] Ali S. (2023). Averaging aggregation operators under the environment of q-rung orthopair picture fuzzy soft sets and their applications in MADM problems. AIMS Mathematics.

[41] Azim A.B. (2023). Industry 4.0 project prioritization by using q-spherical fuzzy rough analytic hierarchy process. AIMS Mathematics.

[42] Azim A.B. (2024). Utilizing sine trigonometric q-spherical fuzzy rough aggregation operators for group decision-making and their role in digital transformation. Heliyon.

[43] Azim A.B. (2024). Assessing indoor positioning system: a q-spherical fuzzy rough TOPSIS analysis. Heliyon.

[44] Mandal U., Seikh M.R. (2023). Interval-valued spherical fuzzy MABAC method based on Dombi aggregation operators with unknown attribute weights to select plastic waste management process. Appl. Soft Comput..

[45] Seikh M.R., Mandal U. (2023). q-Rung orthopair fuzzy Archimedean aggregation operators: application in the site selection for software operating units. Symmetry.

[46] Mandal U., Seikh M.R. (2023). Fuzzy Optimization, Decision-Making and Operations Research: Theory and Applications.

[47] Kahraman C. (2020). Proceedings of the 14th International FLINS Conference (FLINS 2020).

[48] Pawlak Z. (1982). Rough sets. Int. J. Comput. Inf. Sci..

[49] Pawlak Z. (1998). Rough set theory and its applications to data analysis. Cybern. Syst..

[50] Yao Y. (1998). Constructive and algebraic methods of the theory of rough sets. Inf. Sci..

[51] Dai J., Gao S., Zheng G. (2018). Generalized rough set models determined by multiple neighborhoods generated from a similarity relation. Soft Comput..

[52] Zhan J., Sun B. (2020). Covering-based intuitionistic fuzzy rough sets and applications in multi-attribute decision-making. Artif. Intell. Rev..

[53] Sun B. (2022). An approach to MCGDM based on multi-granulation Pythagorean fuzzy rough set over two universes and its application to the medical decision problem. Artif. Intell. Rev..

[54] Garg H., Atef M. (2022). Cq-ROFRS: covering q-rung orthopair fuzzy rough sets and its application to the multi-attribute decision-making process. Complex & Intelligent Systems.

[55] Ashraf S. (2022). A decision-making framework using q-rung orthopair probabilistic hesitant fuzzy rough aggregation information for the drug selection to treat COVID-19. Complexity.

[56] Azim A.B. (2023). q-Spherical fuzzy rough sets and their usage in multi-attribute decision-making problems. AIMS Mathematics.

[57] Wang X., Triantaphyllou E. (2008). Ranking irregularities when evaluating alternatives by using some ELECTRE methods. Omega.

[58] Khan M.A., Khan F., Abdullah S. (2023). Spherical fuzzy rough EDAS method under Einstein aggregation operators applications in cache replacement policy. IEEE Access.

[59] Ashraf S. (2019). Spherical fuzzy sets and their applications in multi-attribute decision-making problems. J. Intell. Fuzzy Syst..

[60] Ashraf S., Abdullah S., Mahmood T. (2020). Spherical fuzzy Dombi aggregation operators and their application in group decision-making problems. J. Ambient Intell. Hum. Comput..

[61] Seikh M.R., Mandal U. (2022). Multiple attribute group decision making based on quasirung orthopair fuzzy sets: application to electric vehicle charging station site selection problem. Eng. Appl. Artif. Intell..

[62] Seikh M.R., Mandal U. (2022). Multiple attribute decision-making based on 3, 4-quasirung fuzzy sets. Granular Computing.

[63] Hussain A. (2024). Decision algorithm for picture fuzzy sets and Aczel Alsina aggregation operators based on unknown degree of wights. Heliyon.

[64] Hussain A., Latif S., Ullah K. (2022). A novel approach of picture fuzzy sets with unknown degree of weights based on schweizer-sklar aggregation operators. Journal of Innovative Research in Mathematical and Computational Sciences.

